# Integrating Genome-Wide Association Analysis With Transcriptome Sequencing to Identify Candidate Genes Related to Blooming Time in *Prunus mume*

**DOI:** 10.3389/fpls.2021.690841

**Published:** 2021-07-15

**Authors:** Man Zhang, Qingqing Yang, Xi Yuan, Xiaolan Yan, Jia Wang, Tangren Cheng, Qixiang Zhang

**Affiliations:** ^1^National Engineering Research Center for Floriculture, Beijing Key Laboratory of Ornamental Plants Germplasm Innovation & Molecular Breeding, Beijing Laboratory of Urban and Rural Ecological Environment, Key Laboratory of Genetics and Breeding in Forest Trees and Ornamental Plants of Ministry of Education, School of Landscape Architecture, Beijing Forestry University, Beijing, China; ^2^Mei Germplasm Research Center, Wuhan, China

**Keywords:** genome-wide association study, gene-based association anaysis, transcriptome sequencing, co-expression network, bloom date, floral bud, *Prunus mume*

## Abstract

*Prunus mume* is one of the most important woody perennials for edible and ornamental use. Despite a substantial variation in the flowering phenology among the *P. mume* germplasm resources, the genetic control for flowering time remains to be elucidated. In this study, we examined five blooming time-related traits of 235 *P. mume* landraces for 2 years. Based on the phenotypic data, we performed genome-wide association studies, which included a combination of marker- and gene-based association tests, and identified 1,445 candidate genes that are consistently linked with flowering time across multiple years. Furthermore, we assessed the global transcriptome change of floral buds from the two *P. mume* cultivars exhibiting contrasting bloom dates and detected 617 associated genes that were differentially expressed during the flowering process. By integrating a co-expression network analysis, we screened out 191 gene candidates of conserved transcriptional pattern during blooming across cultivars. Finally, we validated the temporal expression profiles of these candidates and highlighted their putative roles in regulating floral bud break and blooming time in *P. mume*. Our findings are important to expand the understanding of flowering time control in woody perennials and will boost the molecular breeding of novel varieties in *P. mume*.

## Introduction

Flowering time is one of the most important adaptive traits, which is critical to the fitness and survival of many plant species (Gaudinier and Blackman, 2019). Plants have evolved to decide when to flower by utilizing endogenous signals and environmental cues including day length, temperature, and moisture to maximize reproductive success (Andrés and Coupland, 2012; Sasaki et al., 2018). Most annual or biennial plants flower once in their life cycle and then die (Andrés and Coupland, 2012; Sasaki et al., 2018). Unlike annual plants, perennial species undergo repeated cycles of vegetative and reproductive growth (Singh et al., 2017). As a result, temperate trees synchronize their seasonal development with target environments to avoid harsh climate (Singh et al., 2017; Gaudinier and Blackman, 2019). Premature bud break may have a risk exposure of delicate vegetative meristem or floral primordia to frost damage (Townsend et al., 2018; Gaudinier and Blackman, 2019), while late flowering may lead to a shortened vegetative growth period and mismatched pollination (Ågren et al., 2017; Gaudinier and Blackman, 2019). Within the context of climate change, many phenological events in deciduous trees, including timing of bud set, timing of bud burst, and blooming time were disrupted by warm winters and unpredicted extreme weathers (Aitken et al., 2008; Luedeling, 2012; Fadón et al., 2020a). The elevated temperatures can cause insufficient winter chill that leads to low burst rate, erratic flowering, and poor fruit set in many temperate fruit crops (Celton et al., 2011; Dirlewanger et al., 2012; Abbott et al., 2015). Therefore, it is essential to obtain a comprehensive understanding of the mechanism for the control of timing of bud break and blooming in perennial trees and to breed new varieties adapted to future climate scenarios (Aitken et al., 2008; Luedeling, 2012; Fadón et al., 2020a).

The regulation of flowering time has been extensively studied in model plant species (Srikanth and Schmid, 2011; Song et al., 2013; Cho et al., 2017). With forward and reverse genetic screens, a complex regulation network in *Arabidopsis* was revealed, which consisted of six major pathways, including photoperiod, ambient temperature, gibberellin (GA), vernalization, aging, and autonomous pathways (Srikanth and Schmid, 2011; Khan et al., 2014). These genetic pathways are cross-talked and integrated by a set of integrators, including FLOWERING LOCUS T (FT), LEAFY (LFY), and SUPPRESSOR OF OVEREXPRESSION OF CO1 (SOC1), to regulate floral meristem identity genes such as *APETALA1* (*AP1*), *FRUITFUL* (*FUL*), and *CAULIFLOWER* (*CAL*) (Simpson, 2002; Posé et al., 2012; Khan et al., 2014; Ó'Maoiléidigh et al., 2014). In annual or biennial plants, flowering time is determined by an irreversible switch from vegetative to reproductive growth (Albani and Coupland, 2010). In contrast, flowering in temperate tree species is usually interrupted by a period of dormancy. Basically, floral induction and organ initiation occur in the 1st year, while the flower bud fully develops and blooms in the next year (Albani and Coupland, 2010; Kurokura et al., 2013). Dormant flower buds require a certain period of chilling to break dormancy and become competent to bloom in spring (Fadón et al., 2015, 2018). Thus, the flowering time in temperate trees requires a more complex regulatory network to incorporate environmental cues and mediate the proper timing of flowering (Albani and Coupland, 2010).

In the past decades, the mechanisms regulating bud break and blooming date have been partially uncovered in perennial trees (Yamane et al., 2011). Previous investigations highlighted the conserved role of *FT* gene on flowering and dormancy control in trees (Bohlenius, 2006; Hsu et al., 2011; Wickland Daniel and Hanzawa, 2015). In poplar, two *FT* paralogs displayed multifaceted functions: *FT1* regulates reproductive onset and is hyperinduced by chilling whereas *FT2* is involved in regulating vegetative growth and dormancy cycling (Hsu et al., 2011). Similarly, the constitutive expression of *FT1* in apple was able to induce flowering during *in vitro* cultivation (Kotoda et al., 2010). *CENL1*, an ortholog of *TERMINAL FLOWER 1* (*TFL1*), was also reported to regulate flowering onset and dormancy release in poplar (Mohamed et al., 2010). Another important regulator EARLY BUD BREAK1 (EBB1) is an APETALA2/ethylene responsive factor (AP2/ERF) transcription factor that is found to be associated with bud burst across different tree species (Yordanov et al., 2014; Busov et al., 2015; Anh Tuan et al., 2016). The overexpression of *EBB1* resulted in a precocious bud break in both poplar and Japanese pear (Busov et al., 2015; Anh Tuan et al., 2016). During the bud dormancy release, the expression of *EBB1* increased to be accompanied with the increasing level of cyclin genes and active histone modifications, indicating that cell division mechanism is activated for the flower bud break and enlargement (Anh Tuan et al., 2016). *DORMANCYASSOCIATED MADS-BOX* (*DAM*) genes are also well-known dormancy regulators that were firstly discovered in the dormancy-incapable *evg* (*evergrowing*) peach mutant (Bielenberg et al., 2008; Sasaki et al., 2011; Yamane and Tao, 2015; Niu et al., 2016). The subsequent genetic studies in other tree species have illustrated the importance of *DAM* gene members in regulating floral bud dormancy (Yamane and Tao, 2015; Niu et al., 2016; Wu et al., 2017; Zhao et al., 2018; Balogh et al., 2019). A recent study on peach suggested that chilling can induce the production of small RNAs (sRNAs) and their associated histone methylation (H3K27me3) of *DAM1, DAM3, DAM4*, and *DAM5* in the dormant floral bud, thereby repressing the *DAM* gene expression and promoting dormancy release (Zhu et al., 2020). Chilling temperatures can also inhibit abscisic acid (ABA) accumulation and induce the level of GA and *FT* to promote dormancy break in *Populus* (Rinne et al., 2011; Singh et al., 2018). In spite of the significant advances, the genetic network mediating floral bud break in perennial plants is far from complete (RÃos et al., 2014; Abbott et al., 2015; Fadón et al., 2015; Cattani et al., 2018).

*Prunus mume* Sieb. Et Zucc., also known as Mei or Japanese apricot, is one of the important deciduous fruit crops in East Asia (Zhang et al., 2012; Quast et al., 2013). *P. mume* is native to southern China and was later introduced to Japan, Korea, etc. (Zhang et al., 2012). Similar to many *Prunus* species, the fruit of *P. mume* can be used for edible or culinary purposes (Shi et al., 2019; Bailly, 2020). *P. mume* is also widely used for ornamental and landscaping designs due to its varied flower color, rich fragrance, and early flowering features (Zhang et al., 2012; Shi et al., 2019). *P. mume* trees often initiate and develop reproductive bud from August to October, go through the dormancy period, and start to bloom from late December to March in southern China (Fadón et al., 2015; Zhang et al., 2021). The blooming date highly varies among genotypes and external environments (Zhuang et al., 2013; Shi et al., 2019). To obtain a comprehensive understanding of the genetic determinants of blooming time in *P. mume*, we recorded the flowering phenological traits of 235 Mei landraces for 2 years. We conducted marker-based genome-wide association studies (GWAS) with linear mixed model approach, and the results were integrated by using gene-based association tests to underpin the associated candidate genes for 2017 and 2019. To characterize the functional role of these genes, we performed transcriptome sequencing on floral buds of two *P. mume* cultivars and used co-expression network analyses to identify the associated genes displaying consistent expression profiles during floral bud break across different cultivars. Furthermore, we validated the expression pattern of these candidates using quantitative real-time- (qRT-) PCR assays. Taken together, our study provided new insights into the genetic basis of blooming time variation among the *P. mume* germplasm resources. These findings contribute to the knowledge of the control of floral bud break and flowering time in perennial species and will enable marker-assisted breeding for cultivars suitable for rapidly changing environments.

## Materials and Methods

### Plant Material and Genotyping

Leaves were collected from 235 *P. mume* landraces, and their genomic DNA was extracted by following a cetyl-trimethyl ammonium bromide (CTAB) protocol. Genomic DNA was fragmented, ligated with sequencing adapters at 3'-end, and size selected to construct the sequencing libraries with an insert size of 500 bp. Libraries were then sequenced on the Illumina Hiseq 2000 platform. The detailed procedures in library preparation, sequencing, and raw read data cleaning were described by Zhang et al. (2018). The corresponding raw re-sequencing data are available at National Center for Biotechnology Information (NCBI) sequence read archive (SRA) archive as BioProject PRJNA352648 (SRA accession: SRP093801). All clean reads were aligned to the reference genome of *P. mume* (http://prunusmumegenome.bjfu.edu.cn) by using the Burrows–Wheeler Aligner (BWA) software (Li and Durbin, 2010) and single nucleotide polymorphisms (SNPs) were called following the GATK v3.1 Best Practice pipeline (McKenna et al., 2010). Low-quality SNPs failing the variant filtering criteria (QD < 2.0 || FS > 60.0 || MQ < 40.0 || HaplotypeScore > 13.0) were discarded. We further filtered out SNPs with minor allele frequency (MAF) ≤ 0.05 and a missing genotype rate ≥10%. Finally, the remaining SNPs were imputed with BEAGLE v4.0.

### Measurement of Blooming Time-Related Traits

We measured the blooming dates and leafing dates for 235 accessions over 2 years (from December 1, 2016 to March 31, 2017, and from December 1, 2018 to March 31, 2019). To capture the dynamic progression of flowering, blooming time was described as five sub-traits including timing of the first flower (the date when the first flower was observed on the tree), timing of first 10 flowers (the date when more than 10 flowers were observed), timing of 5% flowering (the date when more than 5% of the floral bud was flushed), timing of 25% flowering (the date when more than 25% of the floral bud was flushed), and timing of 75% flowering (the date when more than 75% of the floral bud was flushed). The timing of leafing was recorded as the date when the first leaf expanded from the leaf bud. All dates were converted to Julian days (days elapsed since January 1 of the recording year) and were normalized to be comparable across years (Calle et al., 2020). Moreover, we estimated a pairwise correlation among days to different stages of flowering and leafing with custom *R* scripts to assess the phenotypic stability across different years. All *P. mume* accessions were planted in a randomized order in the Mei Germplasm Garden, Wuhan, China, and were maintained by a uniform standard without the application of supplemental irrigation during the observational period.

### Population Structure Control

To estimate the hidden population structure, we first removed the SNPs that are in linkage disequilibrium (LD) (*r*^2^ ≥ 0.5) with PLINK 1.9, yielding 1,117,100 pruned SNPs (Purcell et al., 2007). We then assessed the population structure with a principal component analysis (PCA) using “*prcomp”* function in *R* and the fastSTRUCTURE software (Raj et al., 2014). The fastSTRUCTURE analysis was tested on a different number of subpopulations (*K*) ranging from 2 to 10. Due to a weak stratification among the samples, we selected *K* being equal to 10 for the fastSTRUCTURE visualization. Furthermore, we incorporated the scores of leading principal components as covariates (as *Q* matrix) to adjust for the population structure and estimated kinship matrix (as *K* matrix) with TASSEL (Bradbury et al., 2007) to account for the familial relatedness. To find the optimal structured association model, we evaluated the fit of models adjusting for cryptic relatedness (*K* matrix), population substructure (*Q* matrix), or a combined effect (*Q* + *K*) (Zhang et al., 2019). We then chose the optimal model by assessing the Quantile–Quantile plot and selecting the models with genomic inflation factor approximately equal to 1.0 (Supplementary Table 1 and Supplementary Figure 1) (Zhang et al., 2019).

### Gene-Based Association Test

The traditional marker-trait GWAS analysis was firstly performed by using the optimal structured model with a mixed-effect linear model (MLM) implemented in TASSEL version 5.2 (Bradbury et al., 2007). The detailed description for the inbuilt MLM model in TASSEL was described by Bradbury et al. (2007). The raw values of *p* were generated for markers from the association test for each trait and year. We annotated the SNPs with an ANNOtate VARiation (ANNOVAR) pipeline and we considered SNPs with top 0.1% *p*-values as associated SNPs (Wang et al., 2010). The SNP-wise *p*-values were visualized with Manhattan plot by using R package “qqman” (Yin et al., 2021). The LD structure within associated regions was visualized with “LDheatmap” R package (Shin et al., 2006). Furthermore, we estimated haplotype blocks for the highly associated regions based on SNPs within 2 Kb upstream or downstream the border genes using the software PLINK 1.7 (Gabriel, 2002). Haplotype blocks were transformed into multiallelic markers by estimating allele frequencies among 235 accessions. We then regressed the days to the first flower to the haplotype allele frequencies and identified a few most strongly associated blocks.

To further distinguish the association signals from background noises, we performed gene-based analyses with Versatile Gene-based Association Study (VEGAS2) pipeline based on the SNP-wise association results (Mishra and Macgregor, 2014). VEGAS is designed to incorporate combined information from a set of markers within a gene and the pairwise correlation among them (Liu et al., 2010; Mishra and Macgregor, 2014). The VEGAS algorithm first constructs the null distribution of gene-wise test statistics by simulating the sum of squared *Z*-statistics converted from SNP-wise *p*-values and then tests each gene-wise test statistic against the null distribution (Liu et al., 2010; Chung et al., 2019). In this analysis, we included SNPs located within the range of 2.5 Kb upstream to the downstream 2.5 Kb region of 27,819 genes to compute the gene-wise *p*-values. We considered genes with top 5% *p*-values as associated candidates for each trait in 2017 and 2019. The level of overlap among associated SNPs or genes between the traits and across years were tested with a hypergeometric test by using the *dhyper* function in *R*. Candidate genes were annotated with the Pfam protein database (http://pfam.xfam.org) and TAIR database (https://www.arabidopsis.org).

### Transcriptome Sequencing and Differential Expression Gene Detection

To identify important genes involved in flowering process, floral bud tissues with three biological replicates were harvested from an early flowering *P. mume* cultivar “Fenhongzhusha” (“FZ”) and a late flowering cultivar “Subaitaige” (“ST”). Both trees were grafted on uniform rootstocks and were grown in Mei Germplasm Garden, Wuhan, China. Bud samples were collected approximately every 3 weeks from December 21, 2018 till both trees reach full bloom in the subsequent year. The total RNA was isolated, and the RNA integrity was assessed with Agilent 2100 bioanalyzer and gel electrophoresis. Briefly, 27 sequencing libraries were constructed by using Illumina UltraTM RNA Library Prep Kit (NEB, USA) and were sequenced by using paired-end (2 ×150 bp) sequencing on Illumnia Hiseq^TM^ 2000 platform in Novogene Bioinformatics Technology Co., Ltd., Beijing, China. A total of 1.2 billion raw reads were generated and were deposited under the NCBI BioProject PRJNA714446 (SRA accession: SRR13961798-SRR13961824).

Raw reads were cleaned and aligned to the *P. mume* reference genome (http://prunusmumegenome.bjfu.edu.cn) by using HISAT2 v2.05 (Kim et al., 2015). Transcript level was quantified by using the featureCounts tool implemented in Subread (http://subread.sourceforge.net) (Liao et al., 2013) and was converted to fragment per kilo bases per million (FPKM) (Trapnell et al., 2010). PCA was performed based on the FPKM value of all genes by using “*prcomp”* function in *R*. Differential expression analysis was performed with the *R* package “DEseq2” by comparing the gene FPKM between two adjacent individual stages. The *p*-values were corrected by using Benjamini and Hochberg (1995) false discovery rate (FDR) method. Differentially expressed genes (DEGs) were defined as the genes that were differentially expressed with |log_2_(Fold Change)| ≥ 1.5 and FDR < 0.05 in at least one comparison for each cultivar. The gene ontology (GO) enrichment analysis of DEGs was performed by using the “clusterProfiler” R package. GO terms with the corrected *p*-values < 0.05 were considered to be significantly enriched. To validate the accuracy of the RNA-seq analysis, we analyzed the relative expression of a few DEGs with qRT-PCR assays following the protocol listed in the last section “Method.” The primers of selected DEGs for qRT-PCR were provided (Supplementary Table 7).

### Gene Co-expression Network Analysis

To infer the co-expressed gene modules related to flower bud flushing, we extracted the FPKM expression matrix of genes with a variance larger than 0.01 in both cultivars and constructed a co-expression network with the weighted gene correlation network analysis (WGCNA) package (Langfelder and Horvath, 2008) separately for each *P. mume* cultivar. The adjacency matrix was calculated based on Pearson correlations between gene pairs across samples and was converted to a topological overlap matrix (TOM). The corresponding dissimilarity measure (1-TOM) was used for the hieratical clustering of genes with similar expression profiles. We have chosen the soft threshold power *β* = 9 based on the criterion of approximate scale-free topology with *R*^2^ > 0.85. Gene modules were identified by using an automatic network construction function “*blockwiseModule”* with default parameters (minimum module size = 30; mergeCut Height = 0.25) and were labeled with different colors. Module eigen-gene was referred to as the first principal component based on the standardized expression of genes within each module. The expression pattern of module eigen-genes was compared between the two cultivars to identify gene sets with similar expression patterns in different cultivars during floral bud blooming.

### Expression Analysis of Key Candidate Genes Using qRT-PCR

To further validate the candidate gene expression during the blooming progression, we analyzed the expression profile of key candidates among the four *P. mume* cultivars, two early flowering cultivars “Fentaichuizhi” (“FT”) and “Longyou” (“LY”), and two late flowering cultivars “Fenghou” (“FH”) and “Songchun” (“SC”). Floral buds from these trees were sampled every 2–3 weeks from December 2018 to March 2019 until all trees bloom. Total RNA was isolated by using the E.Z.N.A.^®^ Plant RNA Kit (Omega Bio-tek, Norcross, GA, USA) according to the manufacturer's protocol and was reverse transcribed into cDNA by using the PrimeScript RT Reagent Kit (Takara, Japan). Real-time PCR was performed on PikoReal real-time PCR platform (Thermo Fisher Scientific, Dreieich, Germany) by using the SYBR Premix Ex TaqII (Takara, Dalian, China). Reactions were incubated at temperatures set as: 95°C for 30 s; 40 cycles of 95°C for 5 s, 60°C for 30 s, 60°C for 30 s; ending 20°C. Protein phosphatase 2A (PP2A) was used as an internal reference to calculate the relative expression level of target genes using 2^−ΔΔCt^ approach (Livak and Schmittgen, 2001). The relative expression of candidate genes identified in the integrated analysis was compared across different cultivars and developmental stages. Primers for the selected candidate genes used in the qRT-PCR experiments were listed (Supplementary Table 7).

## Results

### Phenotypic Variation in Flowering Phenological Traits

**Figure 1:**
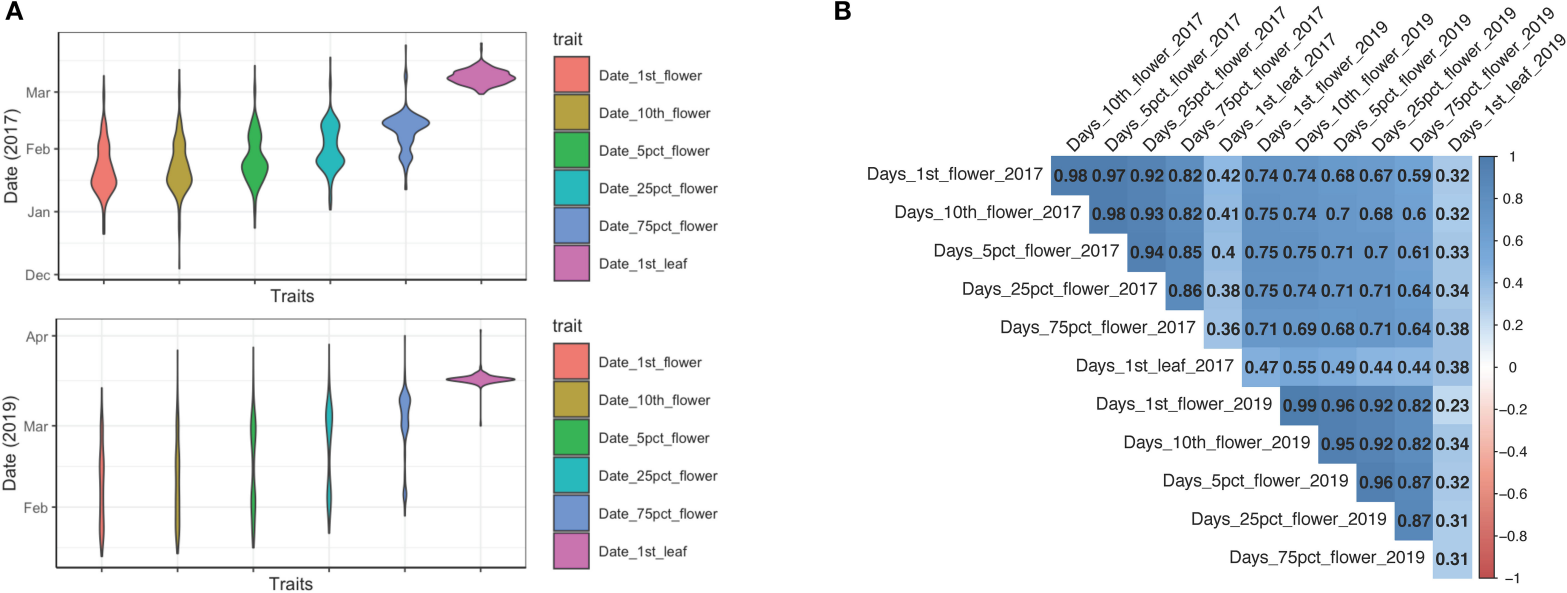
The phenotypic analysis of six phenological traits over 2 years. **(A)** The violin plot of six traits (date of the first flower, date of the first 10 flowers, date of 5% flowering, date of 25% flowering, date of 75% flowering, and date of leaf flushing) collected in 2017 and 2019. **(B)** Pearson correlation coefficients among traits in 2017 and 2019. The pair-wise correlations were colored according to the scale bar.

The timing of blooming and leaf bud breaking are important spring phenological events for temperate fruit perennials. In this study, we measured five flowering phenological traits, including the date of the first flower appearance, the first 10 flowers, 5% flower blooming, 25% flower blooming, 75% flower blooming, and the timing of leaf flushing for 2 years. We observed a high degree of phenotypic variation among 235 Mei accessions over years (Figure 1A). The blooming periods extended from January to late March, then followed by leaf bud flushing and vegetative growth in April (Figure 1A). All investigated individuals displayed a strong consistency in flowering time-related traits across 2 different years (all correlation coefficients *r*^2^ ≥ 0.64; all values of *p* < 0.001) (Figure 1B; Supplementary Table 2). The timing of leafing showed the least consistency across years with a correlation coefficient *r*^2^ = 0.38 (Figure 1B; Supplementary Table 2). A strong pair-wise correlation was also observed among the five flowering time-related sub-traits within each year (2017: correlation coefficients between 0.82 and 0.98; 2019: correlation coefficients between 0.82 and 0.99) (Figure 1B; Supplementary Table 2). However, the timing of leafing showed a weak correlation with the five flowering time-related traits (2017: correlation coefficient: 0.36–0.42; 2019: correlation coefficient: 0.23–0.34) (Figure 1B; Supplementary Table 2). Therefore, we only considered the five flowering time-related traits in the subsequent association analysis. Based on the flower onset time in 2017, we classified 235 cultivars into early flowering, middle flowering, late flowering, and very late flowering cultivars.

### Marker-Based Genome-Wide Association Analysis

**Figure 2:**
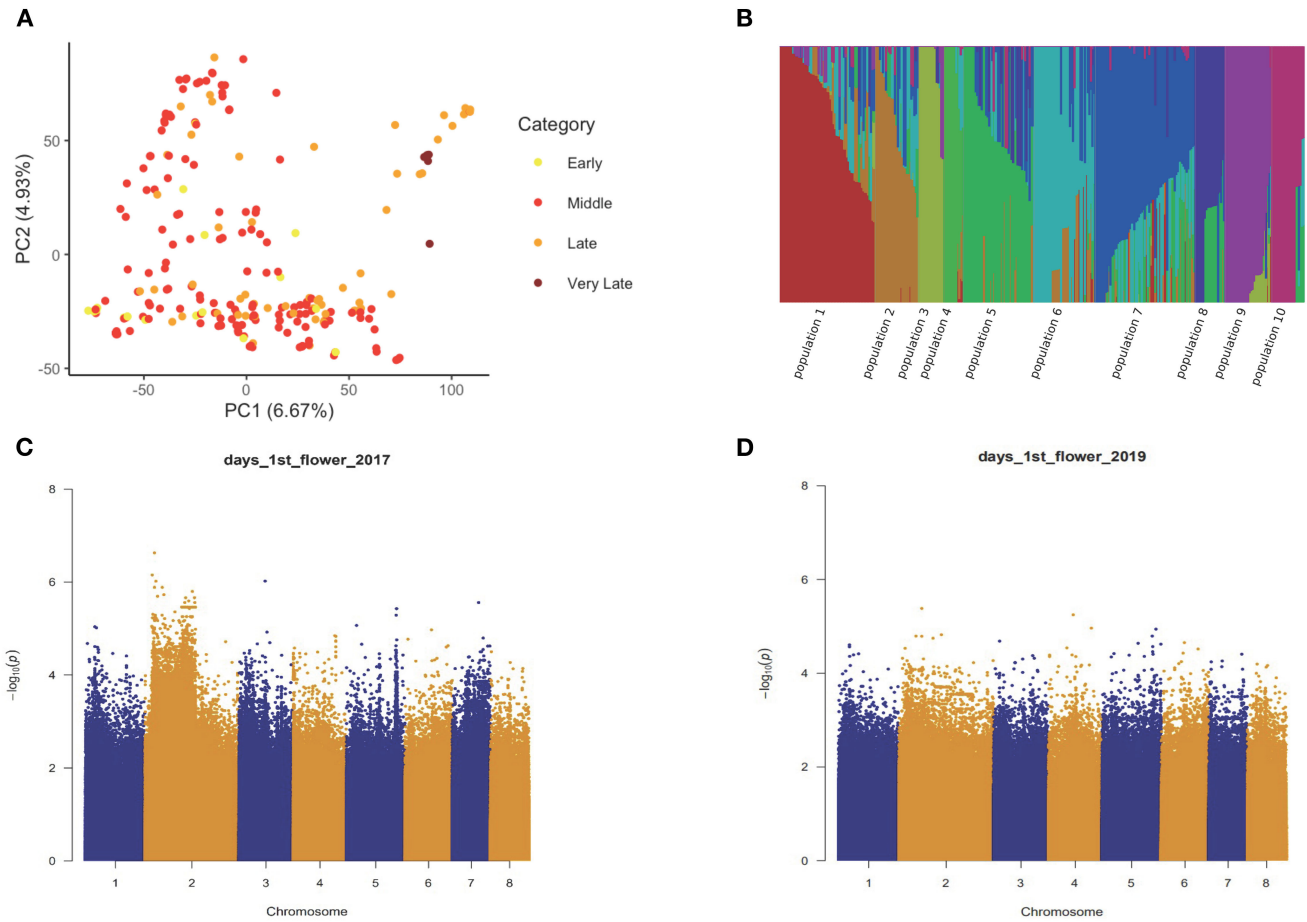
The population structure analysis and single nucleotide polymorphism- (SNP-) based association tests of flowering time-related traits. **(A)** principal component analysis (PCA) biplot of 235 *Prunus mume* accessions. **(B)** fastSTRUCTURE analysis of 235 *P. mume* accessions based on *k* = 10. **(C,D)** Manhattan plot of –log_10_ (value of *p*) for SNP-wise associations with timing of the first flower in 2017 **(C)** and 2019 **(D)**.

We obtained a total of 4,046,973 SNP markers with MAF higher than 0.05 covering eight chromosomes for the 235 accessions. PCA on the SNP set revealed no obvious population stratification among early flowering, middle flowering, late flowering, and very late flowering accessions (Figure 2A). The fastSTRUCTURE analysis confirmed that the sampled individuals are highly admixed with a weak substructure (Figure 2B). By accounting for the effect of possible population structure and familial relatedness, we performed SNP-based association tests on the five traits separately and detected a large number of associated SNP for each trait in both years (Table 1; Figures 2C,D; Supplementary Figure 2). By comparing the significant marker-trait associations across years, we detected a few SNPs repeatedly associated with the five phenological traits in 2017 and 2019 (hypergeometric test: the value of *p* < 1.0 e^−6^) (Table 1). In total, we identified 108 overlapping SNPs, 46 overlapping SNPs, 85 overlapping SNPs, 27 and 28 overlapping SNPs associated with timing of the first flower in 2017 and 2019, timing of the first 10 flowers, timing of 5% flowering, timing of 25% and 75% flowering, respectively (Table 1). We also observed a significant overlap among the SNPs that are significantly associated with multiple traits in each year (2017: 130 SNPs associated with all five sub-traits; 2019: 113 SNPs associated with all five sub-traits).

**Table 1:** The number of associated single nucleotide polymorphisms (SNPs) and candidate genes identified from marker- and gene-based association tests and their overlap between 2017 and 2019.

**Traits**	**2017**		**2019**		**Overlap**	
	**SNP-based**	**Gene-based**	**SNP-based**	**Gene-based**	**SNP-based**	**Gene-based**
	**Top 0.1%**	**Top 5%**	**Top 0.1%**	**Top 5%**	**Top 0.1%**	**Top 5%**
Days to 1st flower	565	1,383	3,306	1,381	108^***^	472^***^
Days to 10th flower	441	1,393	3,359	1,382	46^***^	568^***^
Days to 5pct flower	3,020	1,383	3,386	1,381	85^***^	459^***^
Days to 25pct flower	787	1,382	3,539	1,395	27^***^	284^***^
Days to 75pct flower	3617	1,381	3,620	1,381	28^***^	182^***^

*The significance level of overlap was tested with a hypergeometric test, with*

*** *indicating the value of p < 1e^−6^*.

By comparing associated markers across all studies, we obtained a total of 496 SNPs displaying significant association signals with blooming dates in 2017 and 2019 (Supplementary Table 3). These include 289 intergenic SNPs, 130 intronic SNPs, and 77 exonic SNPs consisting of 38 non-synonymous and 39 synonymous SNPs (Supplementary Table 3). The shared associated SNPs were scattered on eight chromosomes but were mostly discovered on Chromosome 2. These SNPs were further annotated to 360 candidate genes, which were mainly involved in biological processes such as reproduction (GO:0000003), organ development (GO:0048513), and the regulation of cell size (GO:0008361). Among the associated SNPs, one non-synonymous SNP (Chr2_4811832) was located within the 7th exon of *DAM6* (Pm004415) and can cause the amino acid change from 203D to 203G. This SNP was found to be associated with timing of the first flower in 2017 and 2019. We also detected a few nonsynonymous SNPs located within genes such as Pm005060 (UDP-glycosyltransferase superfamily protein), Pm005284 (Unknown protein kinase), and Pm005349 (Cyclin) (Supplementary Table 3). One SNP located upstream of Pm004575 (putative cytokinin-O-glucosyltransferase 2) was associated with timing of the first flower and timing of 10 flowers in 2017 and 2019 (Supplementary Table 3). These genetic variants are promising targets for functional validations.

### Gene-Based Association Analysis Identified Blooming Time-Related Genes

**Figure 3:**
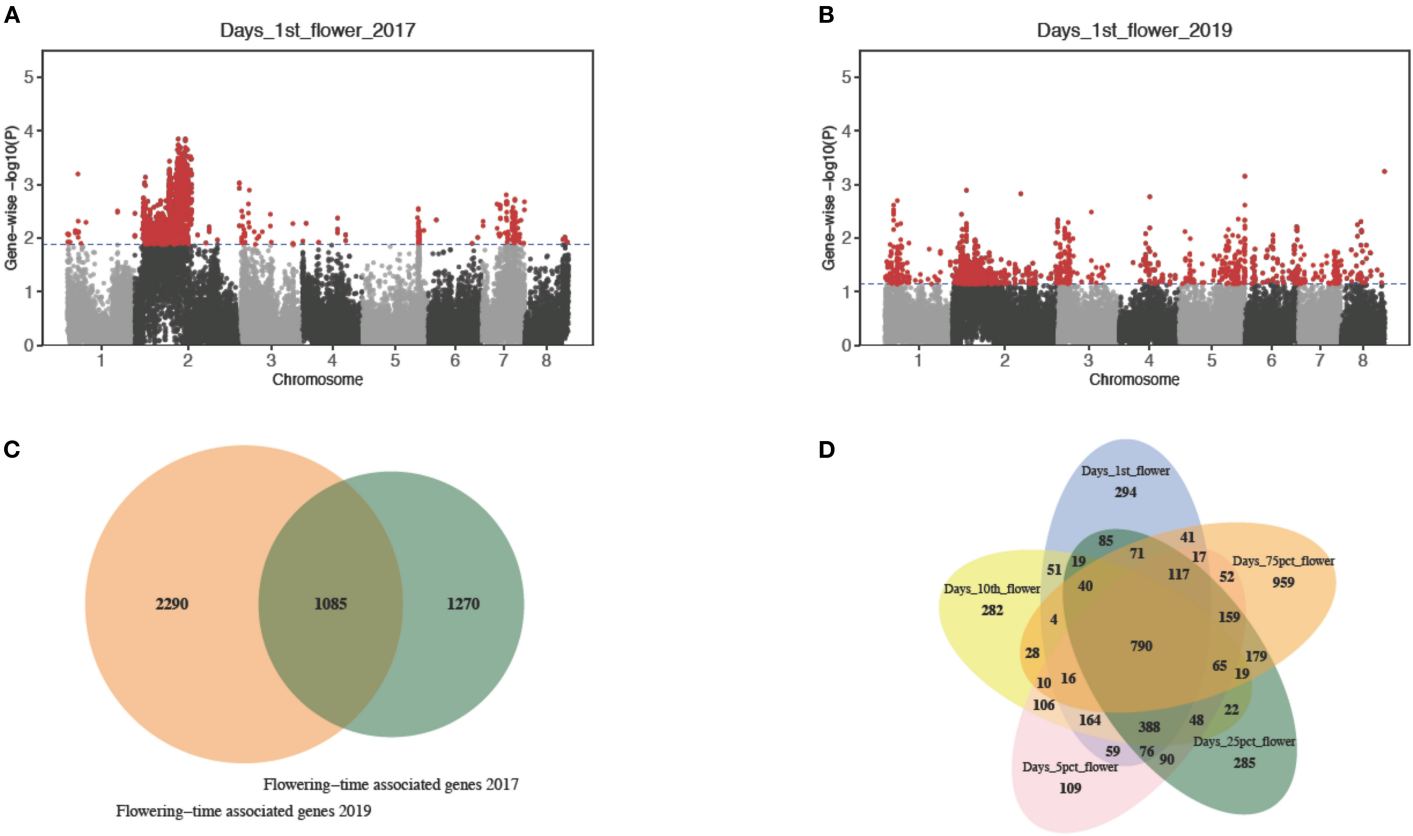
Gene-based association analysis of bloom dates. **(A)** Manhattan plot of gene-wise associations for days to the first flower in 2017. **(B)** Manhattan plot of gene-wise associations for days to the first flower in 2019. **(C)** The Venn diagram comparing flowering time-associated genes detected in 2017 and 2019. **(D)** The Venn diagram comparing candidate genes associated with the five sub-traits, respectively.

Based on the summary statistics of the marker-based test results, we computed the gene-level *p*-values using VEGAS2 (Mishra and Macgregor, 2014) and selected genes with the top 5% gene-wise *p*-values as putative candidates (Table 1; Figures 3A,B). Gene-based test has proved to have more power in identifying functional genetic variants and allow a direct comparison across different studies or mapping populations (McCarthy et al., 2011). With a gene-based association analysis, we obtained 1,383, 1,393, 1,383, 1,382, and 1,381 candidate genes associated with timing of the first flower, timing of the first 10 flowers, timing to 5% flowering, timing of 25% flowering, and timing of 75% flowering, respectively, in 2017 (Table 1). Similarly, we identified 1,381 candidate genes associated with days to the first flower, 1,382 with days to the first 10 flowers, 1,381 with days to 5% flowering, 1,395 with 25% flowering, and 1,381 with 75% flowering in 2019 (Table 1). We observed an extensive level of overlap among the candidate genes associated with five flowering traits across 2 years (all hypergeometric test: value of *p* < 1.9 e^−34^; Table 1). By summing up all candidates, we detected 1,085 flowering time-related genes in both years and 790 candidate genes associated with all five sub-traits in both years (Figures 3C,D). We also observed 294, 282, 109, 285, and 959 associated genes that were specific to timing of the first flower, timing of the first 10 flowers, timing of 5% flowering, timing of 25% flowering, and timing of 75% flowering, respectively (Figure 3D).

Among the top candidates, several genes were previously reported in other perennial trees (Supplementary Table 4). For example, we identified a putative ortholog of *AINTEGUMENTA* (*ANT*; Pm005440) associated with timing of the first flower and timing of the first 10 flowers in 2017 and 2019 (Supplementary Table 4). *ANT* encodes an AP2-like ethylene-responsive transcription factor required for cell proliferation and regulates the floral organ initiation, growth, and patterning in *Arabidopsis* (Krizek, 2015). ANT-like 1 was also involved in the short-day-mediated growth cessation in hybrid aspen (Azeez et al., 2014). D-type CYCLINs including CYCLIN D2 (Pm004529), CYCLIN D3 (Pm005326), and a few A-type CYCLINs, such as CYCLIN A3 (Pm005349) and CYCLIN A1 (Pm004741) were found to be associated with multiple traits in both years (Supplementary Table 4). In poplar, ANT-LIKE 1 gene can regulate the cell cycle through CYCLIN D3.1 to control seasonal growth cessation and resumption (Azeez et al., 2014). It is likely that *ANT-CYCLIN* regulon plays a similar role in the control of floral bud break. We also detected a number of hormonal regulators, for instance, GA20OX5 (GA 20-OXIDASE 5; Pm004371), GA2OX2 (Pm010412), GA3OX1 (Pm004966), and GA20OX3 (Pm004376), which are known to participate in GA biosynthesis and are significantly associated with timing of the first flower and 5% flowering (Supplementary Table 4). Another candidate Pm005288, encoding a bZIP transcription factor ABA RESPONSIVE ELEMENTS-BINDING FACTOR 2 (ABF2), is consisted of significant SNPs associated with timing of the first flower, the first 10 flowers, and 5% flowering in 2017 and timing of the first flower in 2019 (Supplementary Table 4). ABF2 was recognized as a key regulator for bud endodormancy in peach and can interact with TEOSINTE BRANCHED1/CYCLOIDEA/PROLIFERATING CELL FACTOR 20 (TCP20) during the flower bud dormancy release (Leubner et al., 2020).

**Figure 4:**
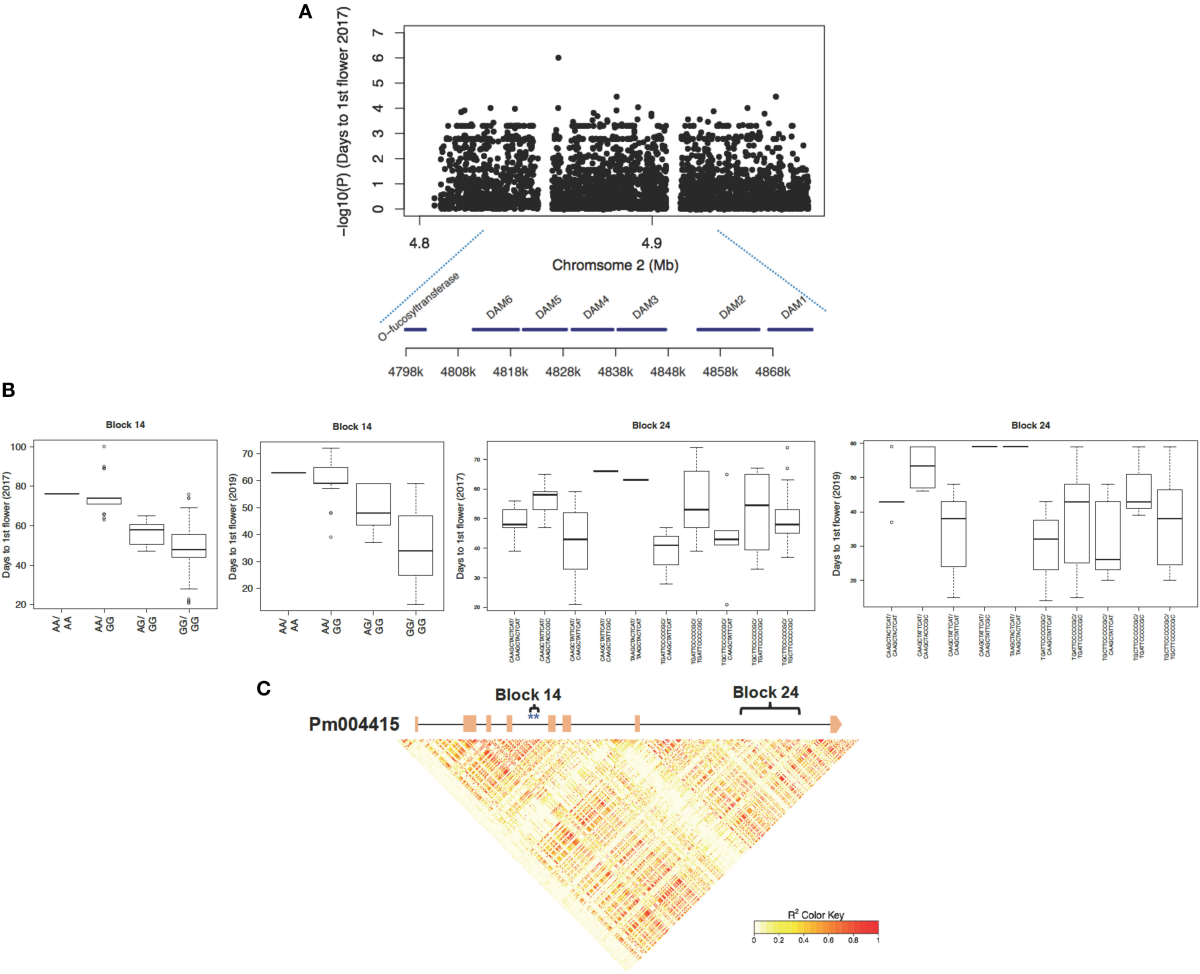
The *DORMANCY-ASSOCIATED MADS-BOX* (*DAM*) gene containing a region associated with timing of blooming in 2017 and 2019. **(A)** Snapshot of SNP-trait associations with timing of the first flower in 2017. **(B)** Haplotypes within Pm004415 (*DAM6*) exhibiting a correlation with timing of the first flower in 2017 and 2019. **(C)** The local linkage structure among SNPs within the 2 Kb upstream/downstream region of Pm004415. Exons of gene Pm004415 were symbolized with orange squares and associated SNPs were labeled with blue stars.

On Chromosome 2, we detected a 77.4 Kb region containing a cluster of genes from Pm004414 to Pm004420, which possessed an excessive number of SNPs strongly associated with timing of flowering in both years (Figures 4A,B). Among candidate genes within this region, Pm004415–Pm004420 encoding six tandem duplicated *DAM* genes, were key dormancy cycling regulators in temperate fruit crops, such as peach, apple, and pear (Bielenberg et al., 2008; Niu et al., 2016; Wu et al., 2017). Among the six *DAM* genes, *DAM5, DAM6*, and *DAM3* exhibited a stronger phenotypic association than the other *DAM* homologs (Figure 4; Supplementary Figure 3). To further distinguish functional variants within this region of high LD, we conducted the haplotype block analysis based on 1,984 SNPs spanning from 4.79 to 4.88 Mb region on Chromosome 2 and obtained 250 haplotypes. By associating the haplotypes with days to the first flower, we identified a few haplotypes within *DAM6, DAM5*, and *DAM3* exhibiting a strong correlation (Figure 4B; Supplementary Figure 3). For example, haplotype block 14 consists of two associated SNPs (Chr2:4812697; Chr2:4812732) located within the fourth intron of *DAM6* (Figure 4C). We observed that the flower onset time of individuals with haplotype GG/GG in block 14 (located within the fourth intron of *DAM6*) is much earlier than that of individuals possessing A allele (AA/AA or A-/GG) in the tests of 2017 and 2019 (Figure 4B).

Moreover, we discovered a few candidate genes consisting of significantly associated SNPs that were not previously characterized to regulate the flowering time in tree species. For example, we detected a few ERF/AP2 transcription factors, including Pm004272 [ETHYLENE RESPONSE FACTOR22 (ERF22)], Pm004616 (SHN2, SHINE2), and Pm004870 [DEHYDRATION RESPONSE ELEMENT-BINDING PROTEIN 26 (DREB26)], that were associated with multiple blooming time-related traits in 2017 and 2019 (Supplementary Table 4). In addition to *DAM* genes, a few MADS-box family genes were also found to be associated with timing of blooming in 2017 and 2019 (Supplementary Table 4). Pm004349 and Pm004350 both encode AGAMOUS-like 16 (AGL16), which form a regulatory module with miR824 to regulate the flowering time in *Arabidopsis* (Hu et al., 2014). Another floral homeotic gene, Pm006266 (APETALA3) also specifies the identity of petal and stamen by interacting with PISTILLATA in *Arabidopsis* (Wang et al., 2019). Pm018089, which encoded an ortholog of *Arabidopsis* SOC1, was shown to be associated with timing of 75% flowering in 2017 and 2019 (Supplementary Table 4).

### Transcriptome Analysis and Co-expression Network Construction

To further explore the functional role of trait-associated genes, we sampled the flower buds from an early flowering cultivar “FZ” and a late flowering cultivar “ST” from late December 2018 to March 15, 2019. The phenological stages of floral buds were characterized as the dormancy phase, bud breaking, bud swelling, and full bloom based on morphological characters (Supplementary Figure 4). We performed transcriptome sequencing on 27 samples (12 “FZ” samples and 15 “ST” samples) and generated a total of 1,414,016,106 raw reads (Supplementary Table 5). After filtering low-quality reads, we obtained 42.45–62.59 million clean reads per sample, and a unique mapping rate of clean reads was 86.78% on average across samples (Supplementary Table 5). In the PCA analysis, the first PC dimension, which explained 47.56% of the total variation, distinguishes the floral buds of different sampling stages, while the second PC splits the samples by cultivar (Supplementary Figure 4). With the differential expression analysis, we identified 5,492 DEGs that were differentially expressed between the adjacent stages for the cultivar “FZ” and 7,163 DEGs for the cultivar “ST.” Among them, 3,630 DEGs were common to both cultivars. We validated the expression profiles of six DEGs using qRT-PCR and we observed that the relative expression pattern is consistent with the RNA-seq results of cultivars “FZ” and “ST,” suggesting the reliability of transcriptome data (Supplementary Figure 5).

**Figure 5:**
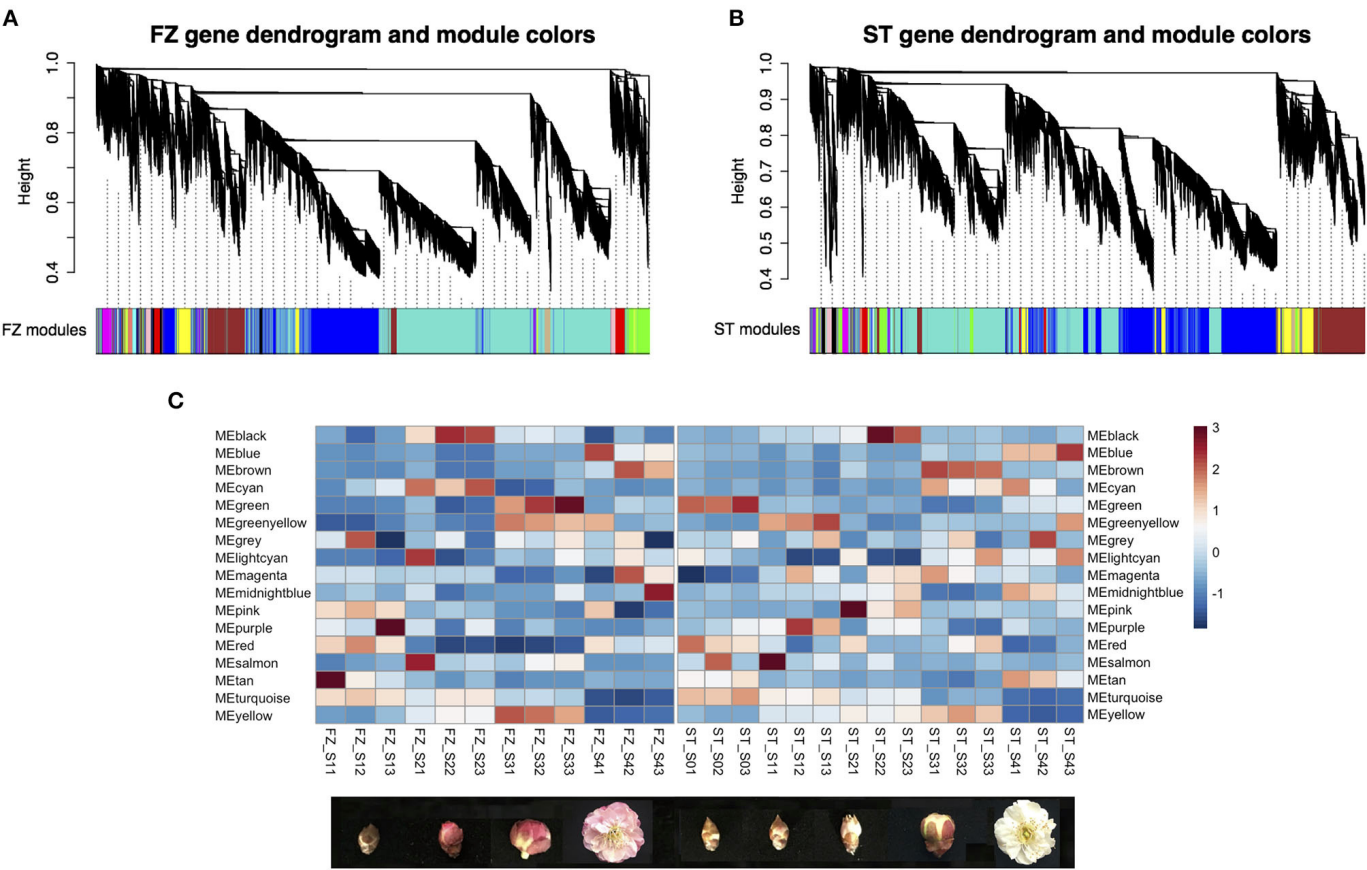
Weighted co-expression network analysis for *P. mume* cultivar “Fenhongzhusha” (“FZ”) and “Subaitaige” (“ST”) **(A)** Gene modules identified for cultivar “FZ,” **(B)** Gene modules identified for cultivar “ST,” **(C)** Expression pattern of eigen-genes for 16 modules during flowering progression in two cultivars.

A weighted gene co-expression network analysis was performed on 19,375 genes separately for two cultivars following the standard WGCNA procedures. We found 16 distinct modules in each network with a module size ranging from 45 (module lightcyan) to 9,157 genes (module turquoise) for the cultivar “FZ” and from 40 (module lightcyan) to 7,086 genes (module turquoise) for the cultivar “ST” (Figures 5A,B). Four (module turquoise, blue, brown, and yellow) of the 16 network modules contained the most blooming time-associated genes in both cultivars, while only module tan in the network of “ST” was significantly enriched for associated candidates (OR = 1.69, Fisher's exact test value of *p* = 0.0278) (Table 2). To identify functionally conserved gene clusters in two cultivars, we compared the eigen-gene expression and observed a consistent expression trend among genes within module turquoise, yellow, blue, and brown across the two networks (Figure 5C). The turquoise module eigen-genes in both networks showed a constantly decreasing pattern, while the eigen-gene in module blue showed the opposite trend with the highest expression in blooming flower tissues (Figure 5C). Since genes can be either positively or negatively correlated with module eigen-genes, there is an abundant gene overlap between module blue and module turquoise across the two networks (i.e., 2,474 overlapping genes between “FZ” MEturquoise and “ST” MEblue and 1,430 genes shared by “FZ” MEblue and “ST” MEturquoise). The eigen-gene of module yellow was constantly increasing before floral buds fully open in both networks (Figure 5C). Additionally, module brown in the network of “FZ” displayed a similar transcription pattern as that of module blue in network “ST” (Figure 5C). The remaining modules that are highly specific to certain genotypes or samples were neglected.

**Table 2:** The number of genome-wide association study (GWAS) candidates within the modules identified from co-expression network analyses for *Prunus mume* cultivar “Fenhongzhusha” (“FZ”) and “Subaitaige” (“ST”).

**Cultivar**	**Module**	**Module Size**	**GWAS-candidates**	**GWAS-candidate (%)**	**Odds ratio**	**Fisher *P*-value**
“FZ”	MEblack	360	34	9.44%	0.744	0.105
“ST”	MEblack	301	27	8.97%	0.703	0.092
“FZ”	MEblue	4,163	519	12.47%	1.027	0.612
“ST”	MEblue	6,096	729	11.96%	0.963	0.436
“FZ”	MEbrown	1,869	221	11.82%	0.958	0.603
“ST”	MEbrown	2,179	258	11.84%	0.959	0.579
“FZ”	MEcyan	137	18	13.14%	1.085	0.696
“ST”	MEcyan	56	5	8.93%	0.702	0.545
“FZ”	MEgreen	723	80	11.07%	0.889	0.355
“ST”	MEgreen	767	93	12.13%	0.989	0.955
“FZ”	MEgreenyellow	249	31	12.45%	1.020	0.922
“ST”	MEgreenyellow	172	17	9.88%	0.785	0.413
“FZ”	MElightcyan	45	3	6.67%	0.512	0.361
“ST”	MElightcyan	40	8	20.00%	1.796	0.144
“FZ”	MEmagenta	316	27	8.54%	0.666	0.046
“ST”	MEmagenta	207	25	12.08%	0.985	1.000
“FZ”	MEmidnightblue	86	9	10.47%	0.838	0.742
“ST”	MEmidnightblue	52	8	15.38%	1.305	0.522
“FZ”	MEpink	323	44	13.62%	1.133	0.441
“ST”	MEpink	251	25	9.96%	0.791	0.332
“FZ”	MEpurple	262	37	14.12%	1.182	0.343
“ST”	MEpurple	177	20	11.30%	0.913	0.818
“FZ”	MEred	527	67	12.71%	1.046	0.736
“ST”	MEred	346	40	11.56%	0.936	0.804
“FZ”	MEsalmon	162	12	7.41%	0.572	0.070
“ST”	MEsalmon	81	9	11.11%	0.896	0.866
“FZ”	MEtan	209	29	13.88%	1.157	0.458
“ST”	MEtan	126	24	19.05%	1.694	0.0278*
“FZ”	MEturquoise	9,157	1,142	12.47%	1.042	0.357
“ST”	MEturquoise	7,086	897	12.66%	1.063	0.179
“FZ”	MEyellow	773	96	12.42%	1.018	0.867
“ST”	MEyellow	1,419	183	12.90%	1.067	0.424

Gene modules enriched with GWAS candidate genes was marked with ^*^ (value of p < 0.05 in the Fisher's exact test).

### Combined Analysis of Gene-Based Association Tests and Co-expression Network

**Figure 6:**
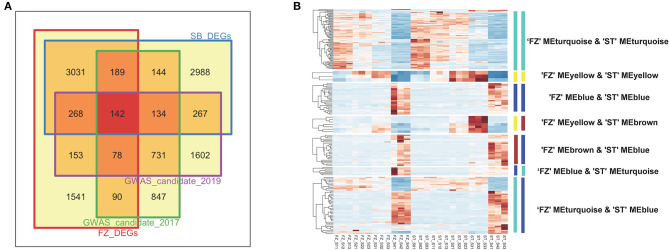
Integration of GWAS analysis with transcriptome sequencing. **(A)** Venn diagram comparing GWAS-identified candidate genes and differential expression genes (DEGs) detected for cultivar “FZ” and “ST,” **(B)** Heatmap of 191 candidate genes displaying consistent expression pattern during blooming process in two cultivars. The gene sets were color-labeled corresponding to each module in the network of “FZ” and “ST,” respectively.

To further discriminate blooming time-related genes, we first integrated the associated genes from a gene-based analysis using the DEGs identified from a transcriptome analysis (Figure 6). We obtained a total of 2,355 and 3,375 candidate genes for blooming time-related traits collected in 2017 and 2019, respectively. Among them, 599 genes were differentially expressed in both cultivars “FZ” and “ST” during the flowering process, and 142 of them were within the intersection set of four candidate lists (Figure 6A). As a complementary analysis to the gene-based association test, we used DEGs to intersect the 360 candidate genes identified from the maker-based association analysis and identified 51 associated genes differentially expressed in both cultivars “FZ” and “ST” (Supplementary Figure 6). After summing up all intersecting DEGs using the gene- and SNP-based analysis, we mapped the 617 candidates to the co-expression networks and considered only 191 candidate genes with a consistently expressing pattern during blooming in both cultivars as the most promising candidates (Figure 6B). These include 83 genes from a turquoise module, 26 genes from blue modules, three genes from yellow modules, and 79 intersection genes from the different modules of two networks (Supplementary Table 8). The overall median correlations |*r|* among the 191 candidate genes were 0.601, and the median correlation among genes within the same module was more than 0.730, indicating that most candidate genes were co-expressed and highly interconnected.

Functional enrichment analysis indicated that these candidate genes are mainly involved in the biological process including carboxylic acid metabolic processes (GO:0019752), response to hormone (GO:0009725), and response to stress (GO:0006950) (Supplementary Table 6). Among the hormonal regulators, genes related to an ABA signaling pathway include CBL-INTERACTING PROTEIN KINASE 20 (CIPK20; Pm010242) required for ABA-mediated seed germination (Gong et al., 2002), cytochrome P450 CYP707A (Pm017952) encoding ABA 8′-hydroxylase (Kushiro et al., 2004), ABA-IMPORTING TRANSPORTER 1 (AIT1; Pm006576), and NINE-CIS-EPOXYCAROTENOID DIOXYGENASE 5 (NCED5; Pm010425), a key enzyme in ABA biosynthesis (Frey et al., 2012) (Supplementary Table 8). Genes that are involved in GA signaling include GA20OX1 (GA 20-oxidase; Pm018083) that is required for GA biosynthesis, GRAS family transcription factor (Pm018822), and a GA-regulated GASA family protein (Pm006215). The expression of Pm018083 increased during flowering but decreased rapidly in fully developed flowers, while the level of Pm018822 maintained a constantly decreasing pattern in both cultivars (Supplementary Figure 7). Genes related to cytokinin metabolism include cytokinin-O-glucosyltransferase 2 (Pm004574, Pm004575, and Pm006589), cytokinin-N-glucosyltransferase 1 (Pm018857), and cytokinin oxidase 5 (Pm006287) (Supplementary Table 8). We observed a constant downregulation of Pm018857 and upregulation of Pm004574 during blooming in cultivars “FZ” and “ST” (Supplementary Figure 7). A number of auxin-responsive genes, including auxin response factor 5 (Pm006237), auxin response factor 3 (Pm010363), and auxin-responsive SAUR family genes (Pm021881 and Pm021894), displayed consistent expression patterns in both cultivars (Supplementary Figure 7). We also detected a few epigenetic regulators, such as VARIANT IN METHYLATION 1 (VIM1; Pm007095) that regulates global CpG methylation (Kakutani et al., 2008), regulator of chromosome condensation (RCC1) (Pm006520 and Pm005158), core and linker histone proteins (Pm014341 and Pm000832), and histone deacetylase (Pm004995). Most of these genes (Pm007095, Pm006520, Pm014341, Pm000832, and Pm004995) showed a constantly decreasing expression pattern during the blooming process in cultivars “FZ” and “ST” (Supplementary Figure 7).

### Expression Analysis of Key Candidate Genes

**Figure 7:**
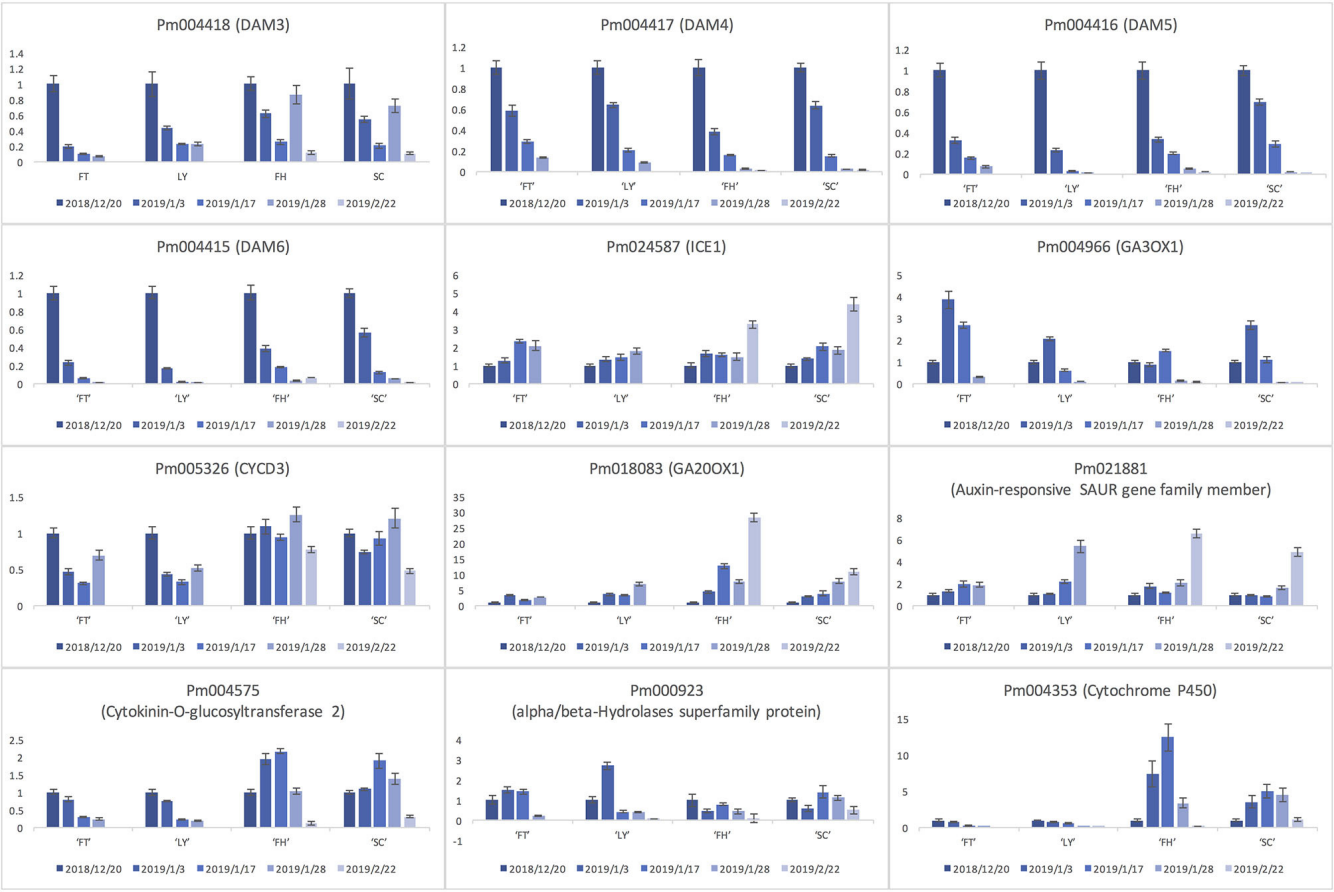
The expression profiles of selected gene candidates during flowering process in four *P. mume* cultivars including “Fentaichuizhi” (“FT”), “Longyou” (“LY”), “Fenghou” (“FH”), and “Songchun” (“SC”).

To verify the candidate gene expression pattern, we assessed the relative expression level of a few candidates in the four *P. mume* cultivars of divergent blooming dates (Figure 7). Among the examined genes, the expression of *DAM4, DAM5*, and *DAM6* significantly decreased among all cultivars as floral buds exit dormancy and bloom (Figure 7). Similarly, the level of *DAM3* in cultivars “FT” and “LY” decreased significantly during blooming, while it first decreased after floral buds exit dormancy, then increased as floral organ develops and decreased again before blooming in late blooming cultivars “FH” and “SC” (Figure 7). We observed a continual increase in the expression of a few genes including a SAUR-like auxin-responsive gene (Pm021881), a GA oxidase (GA20OX1; Pm018083), and a cold responsive protein INDUCER OF CBF EXPRESSION 1 (ICE1; Pm024587) across all four cultivars (Figure 7). On the other hand, Pm004966, which encoded another GA oxidase GA3OX1, displayed slightly different expression profile. Pm004966 first increased during bud break but then decreased as the bud develops into flowers (Figure 7). We also observed a few genes displaying differential expression pattern between early and late flowering cultivars (Figure 7). For example, Pm004575 (Cytokinin-O-glucosyltransferase 2) and Pm004353 (Cytochrome P450) significantly increased as floral bud exits dormancy, peaked in the bud breaking stage, and then decreased as floral organ continues to develop and bloom in cultivars “FH” and “SC.” However, Pm004575 and Pm004353 were continually downregulated during the blooming process in early flowering cultivars “FT” and “LY,” which is consistent with the advanced phenological stage of floral bud in early blooming cultivars (Figure 7). Similarly, the expression of Pm000923 (alpha/beta-hydrolase superfamily protein) in “FT” and “LY” was first increased during bud break, but then decreased continually until the flowers bloom. In late flowering cultivars “SC” and “FH,” Pm000923 first decreased during dormancy release, then followed the same expression pattern as that in early flowering cultivars (Figure 7). In general, the expression pattern of all investigated genes is highly correlated with the progression of floral bud development and blooming across all cultivars.

## Discussion

Flowering time is a key adaptive trait for many temperate plant species. Proper timing of reproductive initiation is essential for plants to avoid unfavorable climatic conditions and achieve pollination success (Aitken et al., 2008; Ågren et al., 2017; Zhang et al., 2019). Flowering time is also a major agronomic trait determining grain yield for many cereal crops (Mathan et al., 2016). In temperate trees, flowering occurs only when the floral buds accumulate sufficient chilling in winter to overcome endodormancy, experience warm temperatures, and finally open in spring (Fadón et al., 2015). Global climate change has shown to affect the flowering phenology of tree crops by disrupting the chilling accumulation rate, damaging floral primordium, and impairing pollination as a consequence of elevated winter temperatures and an increased frost risk (Guo et al., 2013; Allard et al., 2016). Thus, knowing the genetic mechanism to control the flowering phenology is crucial for plant breeders and growers to select the adapted varieties and anticipate crop performance under future climate (Fadón et al., 2020b).

Flowering in temperate tree species is a complex biological process consisting of a series of highly coordinated seasonal events, including dormancy release, floral organ growth, sporogenous tissue development, and floral bud burst (Balogh et al., 2019). The blooming time varies significantly among species and cultivars, and is usually determined by the interaction between chilling requirement, heat requirement, and environmental stimuli (Dirlewanger et al., 2012; Fadón et al., 2020b). Due to the polygenic nature of phenological traits, quantitative genetic approaches were used to reveal the molecular basis for blooming time, chilling requirement, and other phenological traits in temperate trees, including *Populus* (McKown et al., 2018; Zhang et al., 2019), peach (*P. persica*) (Fan et al., 2010), almond (*P. dulcis*) (Sánchez-Pérez et al., 2011), apricot (*P. armeniaca* L.) (Olukolu et al., 2009), sweet cherry (*P. avium*) (Castède et al., 2014), sour cherry (*P. cerasus*) (Cai et al., 2018), and apple (*Malus domestica*) (Celton et al., 2011). With a quantitative trait locus (QTL) analysis, Kitamura et al. (2018) was able to localize the QTLs controlling timing of leaf bud break, chilling and heat requirements of floral bud to a region on linkage group 4 (LG4) that contains *DAM6* gene. By examining the transcript level of *PmDAM6* in the mapping population, they proposed that DAM6 may act as a repressor for bud break in a dose-dependent manner in *P. mume* (Kitamura et al., 2018). A similar QTL study on apple identified a major QTL related to timing of bud break on LG9 that contained orthologs of *ICE1, FLOWERING LOCUS C (FLC)*, and *PACLOBUTRAZOL RESISTANCE 1* (*PRE1*) that are possibly involved in mediating bud break in apple trees (Miotto et al., 2019). With the development of next-generation sequencing, genome-wide association mapping has become an alternative for genetic linkage mapping (Jackson et al., 2011). A recent GWAS for chilling requirement in 480 peach accessions revealed seven association peaks, the strongest among which is located on Linkage Group 1 (LG1) co-localizing with the known *evg* locus (*DAM* gene cluster) in *P. persica* (Li et al., 2019). Till now, the molecular basis underlying the flowering phenology in temperate trees still remains unclear.

*Prunus mume* is one of the earliest spring-flowering tree species in *Rosaceae* family and possesses an extensive phenotypic variation with respect to bloom time among the germplasm collection (Zhang et al., 2012). We investigated the blooming dates of 235 *P. mume* accessions for 2 years. We described the flowering time with five sub-traits, namely the date of the first flower, the date of the first 10 flowers, the date of 5% flowering, the date of 25% flowering, and the date of 75% flowering, to capture the dynamic blooming process. We observed a significant within-population variation in flowering time among individuals, and we evaluated the trait stability from year to year. Despite a slight postpone of blooming dates in 2019 comparing with those in 2017, the floral onset dates were highly correlated, indicating a strong consistency in flowering phenology among individuals despite different environmental factors across years. These findings suggested that blooming time is highly inheritable but can be affected by environmental conditions (Calle et al., 2020). We also detected significant intercorrelations among the five sub-traits, which imply the method's reliability in evaluating blooming time variation for *P. mume* accessions.

Based on the phenotypic traits, we performed GWAS analyses and identified a number of SNP markers associated with blooming time-related traits. Among these loci, some were commonly shared by the five sub-traits and across 2 years in the studied population. GWAS has proven to be a successful approach in identifying the genetic cause of many complex traits in plant species (Tibbs Cortes et al., 2021). However, the traditional GWAS analysis examines marker-based associations and has shown difficulty in assessing rare variants or common variants of small effect size (Eichler et al., 2010). A gene-based GWAS approach was proposed as a complementary strategy by summarizing all SNP associations within a certain gene to estimate the gene-level significance (Liu et al., 2010). Comparing with the traditional GWAS, the gene-based association analysis has increased power in detecting true signals by incorporating both rare and common variants, employing a less stringent significance threshold, and reducing the problem of allelic heterogeneity (Wang et al., 2017). Given these advantages, we undertook gene-based approach to compute the gene-level values of *p* for each trait and year. By examining the commonly associated SNPs and genes between 2 years, we were able to reduce the false positives generated due to different climatic conditions across years. We identified more shared associated candidates between gene-based association test of 2017 and 2019 than that of traditional SNP-based GWAS of 2017 and 2019. The extensive degree of gene overlap suggested that the gene-based association test has shown more power in identifying the candidate genes that may be possibly missed due to a small effect size or masked by background noise in the marker-based analysis. These results also confirmed a stable genetic effect of these loci across years and suggested the superior performance of a gene-based approach over the traditional GWAS in comparing two independent studies conducted at different years.

We observed scattering-associated signals within the genomic regions dispersed to the eight *P. mume* chromosomes, which is likely to result from a polygenic basis for quantitative traits. Among all associated candidate genes or genomic regions, some were co-localized with flowering time-related QTLs reported in the previous studies. For example, we identified a 31.5 Kb region on Chromosome 2 containing *DAM4* (Pm004417), *DAM5* (Pm004416), and *DAM6* (Pm004415), which overlaps with major QTLs for the chilling requirement and bloom date detected in *P. persica, P. armeniaca*, and *P. dulcis* (Olukolu et al., 2009; Fan et al., 2010; Sánchez-Pérez et al., 2011; Romeu et al., 2014). In a recent QTL study, Kitamura et al. also localized one major QTL controlling chilling requirement, bloom date, and leafing date to the *DAM* gene region in *P. mume* (Kitamura et al., 2018). *DAM* genes have been characterized to promote seasonal dormancy in many temperate tree species, including apple (Porto et al., 2016; Wu et al., 2017), pear (Niu et al., 2016), peach (Li et al., 2009), and sweet cherry (Rothkegel et al., 2017). Among six *DAMs, DAM1, 2, 4, 5*, and *6* were found to be responsive to a photoperiod change, while *DAM5–6* were repressed by chilling temperatures (Li et al., 2009; Jiménez et al., 2010). The ectopic expression of *Prunus DAM6* in apple and poplar leads to inhibited growth, early bud set, and delayed bud break (Sasaki et al., 2011; Yang et al., 2019). In a recent study, Zhu et al. reported that *DAM1* and *DAM3–6* were repressed in dormant floral buds during chilling, which are associated with the increasing level of sRNAs and repressive epigenetic marks such as histone H3 lysine 27 (H3K27me3) and CHH methylation in *P. persica* (Zhu et al., 2020). In our study, we observed that *DAM3, DAM5*, and *DAM6* contained the most SNPs associated with timing of the first flower and 5% flowering in 2017 and 2019. Among the associated SNPs, one nonsynonymous SNP in *DAM6* (Pm004415) is predicted to cause an amino acid change within the C-terminal region of DAM6 protein. MADS-box transcription factors normally contained four functional domains, namely the MADS-box domain for DNA binding, the K-domain important for protein–protein interactions, and the I-domain and C-terminal domain with a relatively low sequence conservation (Smaczniak et al., 2012). Though the amino acid conversion may not be necessarily causal, further analysis is required to investigate the function of this variant by using reverse genetics approaches. Due to the high linkage among SNP markers within the genomic region containing *DAMs*, we performed a haplotype analysis and discriminated a few blocks segregating among early and late flowering accessions. These associated haplotypes can be used in the marker-assisted breeding for cultivars with desired flowering time in *P. mume*.

Another blooming time-associated region on Chromosome 2 contained candidate genes including Pm005708, Pm005288, Pm005134, and Pm004913, which are orthologous to the peach genes ppa000228m, ppa006503m, ppa000318m, and ppa013757m within the major QTL for flower date on LG1 in peach (Romeu et al., 2014). These four genes were found to be associated with timing of the first flower, timing of the first 10 flowers, timing of 5% and 25% flowering in 2 years' studies and were located within the genomic region syntenic to the 33.9–37.6 Mb region where major QTLs were located on LG1 (corresponding to peach Chromosome 1). Among these four genes, Pm005708 [PICKLE (PKL)] encodes a SWI (SWItch) nuclear-localized chromatin remodeling factor that can mediate the trimethylation of histone H3 on lysine 4 (H3K4me3) at *FT* locus and can also interact with CONSTANS to promote flowering in *Arabidopsis* (Jing et al., 2019). PKL is also involved in ABA-mediated dormancy induction in *Populus* (Tylewicz et al., 2018). Pm005288 encodes a homolog of ABF2, which was reported to regulate peach bud endodormancy by interacting with TCP20 (Leubner et al., 2020). Pm005134 encodes an ortholog of EMBRYONIC FLOWER 1 (EMF1), which is required to maintain indeterminate growth in *Arabidopsis*. The alternate expression of *EMF1* can lead to transgenic plants with different flowering times (Aubert et al., 2001). Pm004913 encodes protein FLOWERING PROMOTING FACTOR 1 (FPF1), which can interact with the floral organ identity genes to modulate the flowering time in *Arabidopsis* (Melzer et al., 1999). These candidate genes may play a similar role in the control of reproductive development and flowering time in *P. mume*.

We also detected a few candidate genes related to GA biosynthesis, including *GA20OX3* (Pm004376 and Pm005214), *GA20OX5* (Pm004371), and *GA20OX1* (Pm018083), that were found to be overlapped with the candidate genes within the QTLs for both flowering date and chilling requirement on LG4 in *P. avium* (Castède et al., 2014). In *Arabidopsis*, GAs can activate flowering time promoting genes, such as *FT, SOC1*, and *SQUAMOSA PROMOTER BINDING PROTEIN-LIKE* (*SPL*) to promote flowering (Porri et al., 2012). In trees, GA biosynthesis was strongly induced in dormant flower bud or leaf bud during chilling-mediated endodormancy break (Rinne et al., 2011; Barros et al., 2012). These candidate genes may play a role in modulating dormancy release and flowering time through GA signaling in *P. mume*. Additionally, Pm011391 [Ethylene-responsive element binding factor 4 (ERF4)] and Pm000994 [Auxin response factor 4 (ARF4)] identified in our study co-localized with their peach orthologs located within the bloom time-related QTLs on LG4b and LG6a, respectively (Romeu et al., 2014). These overlapping candidate genes discovered across the studies and among *Prunus* species suggested a common transcriptional gene network regulating floral bud break and flowering in *Prunus* species. On the other hand, our study provided a list of novel blooming time-related candidate genes that were mostly involved in flower development, response to abiotic stimulus, and hormonal responses. Whether these genes confer flowering time variation is worthy of further examinations.

To prioritize functionally important associated genes, we performed transcriptome sequencing on floral bud samples of four developmental phases and identified differentially expressed genes between the adjacent stages in the two *P. mume* cultivars. By intersecting the GWAS candidates with DEGs, we found a total of 1,693 and 2,159 GWAS-identified genes among the DEG set of cultivars “FZ” and “ST,” respectively. Since the developmental phase of floral bud in the cultivar “FZ” generally precedes that of “ST” about 2–3 weeks, we expected the DEGs with a consistently expressing trend across sampling stages may contribute to the flowering time variation among cultivars. Therefore, we employed the WGCNA analysis to cluster genes into co-expressed gene modules and mapped the associated loci to the co-expressed gene modules. Finally, we screened out 191 blooming time-associated genes within modules of a consistently temporal expression pattern during the blooming progression in two cultivars (Supplementary Table 8). A large number of gene candidates indicated that a complex polygenic regulatory mechanism is required to mediate the sequential floral development and blooming time in *P. mume* (Penso et al., 2020).

Among the list of candidate genes, Pm006237 (ARF5) and Pm010363 (ARF3) were highly expressed in bursting floral bud and were continuously downregulated until the flower blooms. In *Arabidopsis*, ARF5 was found to be critical in mediating embryo vascular development, and ARF3 has been reported to control the formation of stamens and anther and affect perianth organ number during early flower development (Zheng et al., 2018; Galstyan and Nemhauser, 2019). These two ARFs are putatively involved in the floral organ development and vascular bundle formation in *P. mume*. Cytokinin-O-glucosyltransferase 2 (Pm004574, Pm004575, and Pm006589) and cytokinin-N-glucosyltransferase 1 (Pm018857) are the two types of enzymes catalyzing the glucosylation of cytokinins to maintain the cytokinin homeostasis. During the flower opening process, the expression of Pm004575, Pm006589, and Pm018857 peaked during bud breaking and then decreased, indicating that the cytokinin metabolism is possibly required for the ovule formation and gametogenesis during floral bud development (Bartrina et al., 2011). GAs are important phytohormones that regulate seed germination, floral development, and dormancy cycling (Hedden and Sponsel, 2015). The level of GAs was downregulated during the dormancy induction but was induced after chilling to promote dormancy release and bud break in many deciduous tree species (Liu and Sherif, 2019). On the other hand, GA can promote flowering and is essential for stamen and petal development in *Arabidopsis* (Sun, 2008). In our study, the GA biosynthetic gene *GA20OX1* (Pm018083) was constantly upregulated, while *GA3OX1* (Pm004966) was first induced during bud breaking but was downregulated when flowers continue to expand and bloom. The differential expression pattern of these two genes indicates that they may play divergent roles during flowering in *P. mume*. On the other hand, ABA is known to antagonize GAs by promoting the dormancy establishment and delaying bud break in woody tree species (Liu and Sherif, 2019). The expression of ABA biosynthesis gene *NCED5* (Pm010425) was significantly decreased during bud breaking, suggesting that the reduced endogenous ABA is required for floral bud flush in *P. mume* (Li et al., 2018). In general, the strong activation of genes responsive to growth-promoting hormones (auxin, cytokinin, and GA) and the repression of ABA biosynthesis may lead to fast cell expansion and organ growth in floral bud after dormancy release, and further leads to the early flower opening in *P. mume*.

We also identified a number of chromatin remodeling gene candidates (Supplementary Table 8). For example, *CHLOROPLAST VESICULATION* (Pm004995) encodes a histone deacetylase-like protein that is induced by senescence and abiotic stresses in *Arabidopsis* (Wang and Blumwald, 2014). Pm000832, a histone H2A.4 protein, and Pm014341 that is annotated to linker histone H1 and H5 family, are both chromatin structural proteins important for chromatin organization and posttranscriptional gene silencing (Wang et al., 2012). All three genes were downregulated after floral bud exits dormancy and develops into flowers, which may imply the decreased level of chromatin remodeling during flowering. Additional epigenetic regulators include VIM1 that encodes a set of SRA domain methylcytosine-binding proteins (Kakutani et al., 2008), and RCC1 family proteins (Pm006520 and Pm005158). Previous studies have provided evidence of epigenetic mechanisms, including DNA methylation and chromatin modification, in the regulation of seasonal dormancy cycling in perennial trees (RÃos et al., 2014). Considering hybrid aspen, for example, putative histone deacetylases and histone lysin methyltransferase were upregulated during dormancy induction suggesting the repression of some unknown target genes by chromatin compaction, while some histone deacetylases were upregulated upon chilling during dormancy release (Karlberg et al., 2010). In our study, we identified a few chromatin modification regulators that were putatively implicated in regulating floral bud break. However, their specific target genes or genomic regions should be investigated in future studies.

To validate the functional relevance of candidate genes with the blooming time variation, we assessed the expression profiles of 12 genes in four *P. mume* cultivars. Most genes exhibited highly conserved transcriptional pattern across the four developmental stages among cultivars. For example, three *DAM* genes (*DAM4, DAM5*, and *DAM6*) were all significantly repressed for dormancy release and floral bud flushing. The transcript level of *DAM5* and *DAM6* particularly decreased in a slower manner in late flowering cultivars than early flowering cultivars until reaching the minimum in blooming flowers. This decreasing pattern of *DAM4, DAM5*, and *DAM6* during bud break was also observed among apricot cultivars of different chilling requirements (Yu et al., 2020). Previously, DAM5 and DAM6 are characterized as the main regulators for the chilling requirement in floral bud and leaf bud (Zhu et al., 2020). Therefore, the transcript level of *DAM4–DAM6* possibly reflects the progression of the dormancy release toward flowering among different cultivars in *Prunus* species (Jiménez et al., 2010; Falavigna et al., 2019). Being an early flowering tree species, *P. mume* can bloom even under low temperatures in spring (Zhang et al., 2012). It is likely that *P. mume* requires relatively low chilling units to suppress the expression of *DAM5* and *DAM6*, and to promote bud break, which results in its early blooming feature. *DAM3* showed a similar decreasing pattern in early flowering cultivars “FT” and “LY.” However, in late flowering cultivars, *DAM3* expression level first decreased during bud break, then significantly increased before blooming and dropped again in blooming flowers. One possible reason for the differential expression profile of *DAM3* across cultivars could be the missed sampling time points when *DAM3* increased in “FT” and “LY” due to a rapid bud development in early blooming cultivars. A previous study on peach revealed that the expression of *DAM3* is downregulated after exposure to cold temperatures, and is recovered to a relatively high level in growing seasons (Li et al., 2009). The potential role of DAM3 in regulating floral bud post-dormancy development requires further explorations (Li et al., 2009).

Additionally, the general expression trend of auxin-responsive SAUR family gene Pm021881, *ICE1* (Pm024587), and two GA oxidase genes (Pm018083 and Pm004966) was consistent across four cultivars, indicating their conserved functional role in a certain developmental phase. Furthermore, the expression peak of Pm004575 (cytokinin-O-glucosyltransferase 2), Pm000923 (alpha/beta-Hydrolase superfamily protein), and Pm004353 (Cytochrome P450) occurred ~1 month earlier in cultivars “FT” and “LY” than that of late blooming cultivars “FH” and “SC,” suggesting that the expression level and pattern of these candidate genes may reflect the advanced phenological status of floral buds in early flowering cultivars comparing to late blooming cultivars. These results highlighted the possibility that the genetic differences lead to the differential transcriptional state of a few key regulators and eventually cause the variation in flowering time among the *P. mume* germplasm collection. The molecular mechanism connecting the genetic variation with transcriptional variation is still unclear. On the other hand, we observed that the expression of these genes is highly correlated with the developmental progression and timing of flowering among different *P. mume* cultivars. However, we could not directly attribute the flowering time variation to the transcription level or pattern of these genes since some of them may function in other biological processes confounded with flowering progression. In future studies, it will be necessary to examine the casual sequence or structural variation of the candidate genes that possibly lead to the transcriptional differentiation and varying flowering time among the mapping individuals and to develop blooming time-related markers for the rapid selection of new cultivars at the seedling stage.

## Conclusions

In this study, we have investigated the blooming time-related traits in 235 accessions of *P. mume* for 2 years. A significant correlation among traits and across years revealed the stable and consistent flowering phenology among *P. mume* accessions. With the marker- and gene-based association analysis, we identified 1,445 genes associated with more than one phenological traits in 2017 and 2019. To screen for functionally relevant candidates, we performed transcriptome sequencing floral buds of two *P. mume* cultivars and obtained 191 candidate genes with consistently expression profiles during blooming by integrating the co-expression network analysis. Furthermore, we validated the expression profile of these candidates using qRT-PCR analysis and confirmed that their expression is highly correlated with the progression of flowering among cultivars. Our findings provide new insights into the genetic architecture underlying blooming time in *P. mume* and will facilitate marker-assisted breeding for adapted cultivars to the new climate scenarios.

## Data Availability Statement

The original contributions presented in the study are publicly available. This data can be found at: The transcriptome sequencing data generated in our study is available at NCBI Sequence Read Archive under BioProject PRJNA714446 (accession number: SRR13961798- SRR13961824).

## Author Contributions

MZ and QZ conceived and designed the study. MZ performed most of the experiment, analyzed the data, and wrote the manuscript. QY and XYu contributed to the qRT-PCR analysis. XYa assisted in phenotyping and collecting floral bud materials. TC and JW provided help with the experiments. All authors contributed to the article and approved the submitted version.

## Conflict of Interest

The authors declare that the research was conducted in the absence of any commercial or financial relationships that could be construed as a potential conflict of interest.
