# Supplementary material for: Integrating Genome-Wide Association Analysis With Transcriptome Sequencing to Identify Candidate Genes Related to Blooming Time in Prunus mume
Source: Front Plant Sci. 2021 Jul 15;12:690841. doi: 10.3389/fpls.2021.690841 (PMC8319914; doi:10.3389/fpls.2021.690841)
Supplement: Supplementary file 1 [file Data_Sheet_1.docx]

Supplementary Material

# Supplementary Figures and Tables

**Figure S1**. The Quantile-Quantile plot of SNP-associations from the optimal structured models for five phenological traits in 2017 and 2019.

**Figure S2**. Manhattan plot of -log_10_(P-value) for SNP-wise associations with timing of first flower, timing of first ten flower, timing of five percent flowering, timing of twenty-five percent flower, timing of seventy-five flowering in 2017 and 2019.

**Figure S3**. Haplotype blocks within *DAM* genes on Chromosome 2 that associated with timing of flowering in 2017.

**Figure S4**. Principal component analysis (PCA) of twenty-seven floral bud samples based on the gene expression data.

**Figure S5**. The validation of DEGs with qRT-PCR analysis. The blue bar indicates the FPKM value of each sample derived from RNA-seq analysis and the redline indicates the relative expression value in the qRT-PCR analysis.

**Figure S6**. The venn diagram comparing 360 candidate genes identified from the marker-based association analysis with DEGs detected for cultivar ‘FZ’ and ‘ST’.

**Figure S7**. The heatmap of 191 GWAS-identified candidates displaying consistent expression pattern during blooming in *P. mume* cultivar ‘FZ’ and ‘ST’.

**Table S1**. The genomic inflation factor (λ_GC_) assessed for all structured association models.

**Table S2**. Pearson correlation coefficients among the flowering-time related traits collected in 2017 and 2019. The significance level of correlation test is labeled as * indicating p-value <0.001, ** indicating p-value <0.000001.

**Table S3**. The list of SNPs significantly associated with flowering time-related traits in both 2017 and 2019.

**Table S4**. Selected top candidates associated with blooming-time related traits from gene-based association tests. Some candidate genes were found related to multiple traits.

**Table S5**. Summary statistics of transcriptome sequencing on twenty-seven floral bud samples from two *P. mume* cultivars.

**Table S6**. The GO enrichment analysis of the 191 associated candidate genes.

**Table S7**. Primers used in qRT-PCR analysis.

**Table S8.** The 191 candidate genes displaying consistent expression pattern during blooming across different *P. mume* cultivars.

## Supplementary Figures

**Figure S1.** The Quantile-Quantile plot of SNP-associations from the optimal structured models for five phenological traits in 2017 and 2019.

**Figure S2**. Manhattan plot of -log_10_(P-value) for SNP-wise associations with timing of first flower, timing of first ten flower, timing of five percent flowering, timing of twenty-five percent flower, timing of seventy-five flowering in 2017 and 2019.

**Figure S3.** Haplotype blocks within *DAM* genes on Chromosome 2 that associated with timing of flowering in 2017. (a) Haplotypes within Pm004416 (*DAM5*) exhibiting correlation with timing of first flower in 2017. The heatmap displays the linkage structure among SNPs within the 2kb upstream/downstream region of Pm004416. (b) Haplotypes within Pm004418 (*DAM3*) exhibiting correlation with timing of first flower in 2017. The heatmap displays the LD structure among SNPs within the 2kb upstream/downstream region of Pm004418. Gene exons were symbolized with orange squares and SNP outliers were labeled with blue stars.

**Figure S4.** Principal component analysis (PCA) of twenty-seven floral bud samples based on the gene expression data.

**Figure S5.** The validation of DEGs with qRT-PCR analysis. The blue bar indicates the FPKM value of each sample derived from RNA-seq analysis and the redline indicates the relative expression value in the qRT-PCR analysis.

**Figure S6.** The venn diagram comparing 360 candidate genes identified from the marker-based association analysis with DEGs detected for cultivar ‘FZ’ and ‘ST’.

**Figure S7.** The heatmap of 191 GWAS-identified candidates displaying consistent expression pattern during blooming in *P. mume* cultivar ‘FZ’ and ‘ST’.

## Supplementary Tables

**Table S1**. The genomic inflation factor (λ_GC_) assessed for all structured association models.

| Trait | Year | Model | λ_GC_ | optimal model | Year | Model | λ_GC_ | optimal model |
| --- | --- | --- | --- | --- | --- | --- | --- | --- |
| 1st flower | 2017 | 1PC | 2.999 |  | 2019 | 1PC | 3.677 |  |
| 10st flower | 2017 | 1PC | 2.974 |  | 2019 | 1PC | 3.692 |  |
| 5pct flowering | 2017 | 1PC | 2.703 |  | 2019 | 1PC | 3.243 |  |
| 25pct flowering | 2017 | 1PC | 2.436 |  | 2019 | 1PC | 3.025 |  |
| 75pct flowering | 2017 | 1PC | 2.536 |  | 2019 | 1PC | 2.958 |  |
| 1st flower | 2017 | 5PC | 1.870 |  | 2019 | 5PC | 2.112 |  |
| 10st flower | 2017 | 5PC | 1.985 |  | 2019 | 5PC | 2.007 |  |
| 5pct flowering | 2017 | 5PC | 1.945 |  | 2019 | 5PC | 1.785 |  |
| 25pct flowering | 2017 | 5PC | 2.013 |  | 2019 | 5PC | 1.682 |  |
| 75pct flowering | 2017 | 5PC | 1.943 |  | 2019 | 5PC | 1.548 |  |
| 1st flower | 2017 | 7PC | 1.538 |  | 2019 | 7PC | 2.107 |  |
| 10st flower | 2017 | 7PC | 1.582 |  | 2019 | 7PC | 2.044 |  |
| 5pct flowering | 2017 | 7PC | 1.654 |  | 2019 | 7PC | 1.821 |  |
| 25pct flowering | 2017 | 7PC | 1.703 |  | 2019 | 7PC | 1.740 |  |
| 75pct flowering | 2017 | 7PC | 1.739 |  | 2019 | 7PC | 1.543 |  |
| 1st flower | 2017 | K | 1.007 | √ | 2019 | K | 1.091 |  |
| 10st flower | 2017 | K | 1.036 | √ | 2019 | K | 1.036 |  |
| 5pct flowering | 2017 | K | 1.006 | √ | 2019 | K | 1.010 |  |
| 25pct flowering | 2017 | K | 1.064 | √ | 2019 | K | 1.003 |  |
| 75pct flowering | 2017 | K | 1.018 | √ | 2019 | K | 0.849 |  |
| 1st flower | 2017 | 1PC+K | 0.920 |  | 2019 | 1PC+K | 1.053 | √ |
| 10st flower | 2017 | 1PC+K | 0.955 |  | 2019 | 1PC+K | 1.108 | √ |
| 5pct flowering | 2017 | 1PC+K | 0.918 |  | 2019 | 1PC+K | 0.964 | √ |
| 25pct flowering | 2017 | 1PC+K | 0.975 |  | 2019 | 1PC+K | 0.865 | √ |
| 75pct flowering | 2017 | 1PC+K | 0.967 |  | 2019 | 1PC+K | 0.685 | √ |
| 1st flower | 2017 | 5PC+K | 0.912 |  | 2019 | 5PC+K | 1.031 |  |
| 10st flower | 2017 | 5PC+K | 0.945 |  | 2019 | 5PC+K | 1.171 |  |
| 5pct flowering | 2017 | 5PC+K | 0.894 |  | 2019 | 5PC+K | 0.981 |  |
| 25pct flowering | 2017 | 5PC+K | 0.936 |  | 2019 | 5PC+K | 0.884 |  |
| 75pct flowering | 2017 | 5PC+K | 0.915 |  | 2019 | 5PC+K | 0.732 |  |
| 1st flower | 2017 | 7PC+K | 0.921 |  | 2019 | 7PC+K | 1.034 |  |
| 10st flower | 2017 | 7PC+K | 0.951 |  | 2019 | 7PC+K | 1.188 |  |
| 5pct flowering | 2017 | 7PC+K | 0.899 |  | 2019 | 7PC+K | 0.979 |  |
| 25pct flowering | 2017 | 7PC+K | 0.976 |  | 2019 | 7PC+K | 0.888 |  |
| 75pct flowering | 2017 | 7PC+K | 0.953 |  | 2019 | 7PC+K | 0.739 |  |

**Table S2**. Pearson correlation coefficients among the flowering-time related traits collected in 2017 and 2019. The significance level of correlation test is labeled as * indicating p-value <0.001, ** indicating p-value <0.000001.

|  | 1st_flower_2017 | 10st_flower_2017 | 5pct_flower_2017 | 25pct_flower_2017 | 75pct_flower_2017 | 1st_leaf_2017 | 1st_flower_2019 | 10st_flower_2019 | 5pct_flower_2019 | 25pct_flower_2019 | 75pct_flower_2019 | 1st_leaf_2019 |
| --- | --- | --- | --- | --- | --- | --- | --- | --- | --- | --- | --- | --- |
| 1st_flower_2017 |  | 0.98 | 0.97 | 0.92 | 0.82 | 0.42 | 0.74 | 0.74 | 0.68 | 0.67 | 0.59 | 0.32 |
| 10st_flower_2017 | ** |  | 0.98 | 0.93 | 0.82 | 0.41 | 0.75 | 0.74 | 0.70 | 0.68 | 0.60 | 0.32 |
| 5pct_flower_2017 | ** | ** |  | 0.94 | 0.85 | 0.40 | 0.75 | 0.75 | 0.71 | 0.70 | 0.61 | 0.33 |
| 25pct_flower_2017 | ** | ** | ** |  | 0.86 | 0.38 | 0.75 | 0.74 | 0.71 | 0.71 | 0.64 | 0.34 |
| 75pct_flower_2017 | ** | ** | ** | ** |  | 0.36 | 0.71 | 0.69 | 0.68 | 0.71 | 0.64 | 0.38 |
| 1st_leaf_2017 | ** | ** | ** | ** | ** |  | 0.47 | 0.55 | 0.49 | 0.44 | 0.44 | 0.38 |
| 1st_flower_2019 | ** | ** | ** | ** | ** | ** |  | 0.99 | 0.96 | 0.92 | 0.82 | 0.23 |
| 10st_flower_2019 | ** | ** | ** | ** | ** | ** | ** |  | 0.95 | 0.92 | 0.82 | 0.34 |
| 5pct_flower_2019 | ** | ** | ** | ** | ** | ** | ** | ** |  | 0.96 | 0.87 | 0.32 |
| 25pct_flower_2019 | ** | ** | ** | ** | ** | ** | ** | ** | ** |  | 0.87 | 0.31 |
| 75pct_flower_2019 | ** | ** | ** | ** | ** | ** | ** | ** | ** | ** |  | 0.31 |
| 1st_leaf_2019 | ** | * | ** | ** | ** | ** | * | ** | ** | * | * |  |

**Table S3**. The list of SNPs significantly associated with flowering time-related traits in both 2017 and 2019.

| Marker | Chr | GeneID | Associated_traits_2017 | Associated_traits_2019 | Function | Type | AA_change | Gene description |
| --- | --- | --- | --- | --- | --- | --- | --- | --- |
| Chr1_1091223 | 1 | Pm000166 | days_5pct&days_25pct&days_75pct | days_5pct&days_25pct&days_75pct | intergenic | . | . | PF00999:Sodium/hydrogen exchanger family |
| Chr1_1091230 | 1 | Pm000166 | days_1st_flower&days_10st_flower&days_5pct&days_25pct&days_75pct | days_1st_flower&days_10st_flower&days_5pct&days_25pct&days_75pct | intergenic | . | . | PF00999:Sodium/hydrogen exchanger family |
| Chr1_12315527 | 1 |  | days_25pct | days_25pct | intergenic | . | . |  |
| Chr1_1503488 | 1 | Pm000239 | days_75pct | days_75pct | downstream | . | . | PF07765:KIP1-like protein |
| Chr1_20987509 | 1 | Pm002697 | days_10st_flower | days_10st_flower | intergenic | . | . | PF00646:F-box domain\|PF08268:F-box associated domain |
| Chr1_22589626 | 1 | Pm002993 | days_1st_flower | days_1st_flower | intergenic | . | . | PF08263:Leucine rich repeat N-terminal domain\|PF00069:Protein kinase domain |
| Chr1_3242536 | 1 | Pm000522 | days_10st_flower | days_10st_flower | intronic | . | . | - |
| Chr1_4209283 | 1 | Pm000691 | days_75pct | days_75pct | intergenic | . | . | - |
| Chr1_4209297 | 1 | Pm000691 | days_75pct | days_75pct | intergenic | . | . | - |
| Chr1_4828944 | 1 | Pm000789 | days_1st_flower&days_10st_flower&days_5pct&days_25pct&days_75pct | days_1st_flower&days_10st_flower&days_5pct&days_25pct&days_75pct | intergenic | . | . | PF03081:Exo70 exocyst complex subunit |
| Chr1_4828981 | 1 | Pm000789 | days_1st_flower&days_10st_flower&days_5pct&days_25pct&days_75pct | days_1st_flower&days_10st_flower&days_5pct&days_25pct&days_75pct | intergenic | . | . | PF03081:Exo70 exocyst complex subunit |
| Chr1_4847479 | 1 | Pm000792 | days_1st_flower&days_5pct&days_25pct | days_1st_flower&days_5pct&days_25pct | upstream | . | . | PF00403:Heavy-metal-associated domain |
| Chr1_4851921 | 1 | Pm000792 | days_1st_flower&days_5pct&days_25pct&days_75pct | days_1st_flower&days_5pct&days_25pct&days_75pct | intergenic | . | . | PF00403:Heavy-metal-associated domain |
| Chr1_4903112 | 1 | Pm000802 | days_5pct&days_25pct | days_5pct&days_25pct | upstream | . | . | PF00926:3,4-dihydroxy-2-butanone 4-phosphate synthase\|PF00925:GTP cyclohydrolase II |
| Chr1_5692481 | 1 | Pm000933 | days_25pct | days_25pct | upstream | . | . | - |
| Chr1_7375183 | 1 | Pm001135 | days_1st_flower | days_1st_flower | intergenic | . | . | PF07011:Protein of unknown function (DUF1313) |
| Chr1_9633193 | 1 | Pm001376 | days_5pct&days_25pct&days_75pct | days_5pct&days_25pct&days_75pct | exonic | synonymous | Pm001376:Pm001376.1:exon1:c.C228T:p.G76G | - |
| Chr2_10093008 | 2 | Pm005260 | days_1st_flower&days_10st_flower&days_5pct | days_1st_flower&days_10st_flower&days_5pct | intergenic | . | . | PF08235:LNS2 (Lipin/Ned1/Smp2)\|PF04571:lipin, N-terminal conserved region |
| Chr2_10093455 | 2 | Pm005260 | days_1st_flower | days_1st_flower | intergenic | . | . | PF08235:LNS2 (Lipin/Ned1/Smp2)\|PF04571:lipin, N-terminal conserved region |
| Chr2_10170314 | 2 | Pm005280 | days_5pct | days_5pct | intronic | . | . | PF00067:Cytochrome P450 |
| Chr2_10170384 | 2 | Pm005280 | days_10st_flower | days_10st_flower | intronic | . | . | PF00067:Cytochrome P450 |
| Chr2_10186696 | 2 | Pm005282 | days_1st_flower&days_10st_flower | days_1st_flower&days_10st_flower | intronic | . | . | - |
| Chr2_10192778 | 2 | Pm005284 | days_1st_flower&days_10st_flower&days_5pct&days_25pct&days_75pct | days_1st_flower&days_10st_flower&days_5pct&days_25pct&days_75pct | intronic | . | . | PF00069:Protein kinase domain |
| Chr2_10192906 | 2 | Pm005284 | days_1st_flower&days_10st_flower&days_5pct&days_25pct&days_75pct | days_1st_flower&days_10st_flower&days_5pct&days_25pct&days_75pct | exonic | nonsynonymous | Pm005284:Pm005284.1:exon2:c.G264A:p.M88I | PF00069:Protein kinase domain |
| Chr2_10193226 | 2 | Pm005284 | days_1st_flower&days_10st_flower&days_5pct&days_25pct&days_75pct | days_1st_flower&days_10st_flower&days_5pct&days_25pct&days_75pct | intronic | . | . | PF00069:Protein kinase domain |
| Chr2_10329497 | 2 | Pm005307 | days_1st_flower | days_1st_flower | exonic | nonsynonymous | Pm005307:Pm005307.1:exon3:c.G1271A:p.G424D | PF02458:Transferase family |
| Chr2_10453807 | 2 | Pm005326 | days_1st_flower&days_10st_flower&days_5pct | days_1st_flower&days_10st_flower&days_5pct | intergenic | . | . | PF02984:Cyclin, C-terminal domain\|PF00134:Cyclin, N-terminal domain |
| Chr2_10459564 | 2 | Pm005327 | days_1st_flower | days_1st_flower | intergenic | . | . | PF01112:Asparaginase |
| Chr2_10502367 | 2 | Pm005335 | days_1st_flower | days_1st_flower | intergenic | . | . | PF00069:Protein kinase domain\|PF01453:D-mannose binding lectin\|PF00954:S-locus glycoprotein domain |
| Chr2_10527249 | 2 | Pm005340 | days_1st_flower&days_10st_flower | days_1st_flower&days_10st_flower | upstream | . | . | PF00646:F-box domain |
| Chr2_10537347 | 2 | Pm005343 | days_1st_flower&days_10st_flower | days_1st_flower&days_10st_flower | exonic | nonsynonymous | Pm005343:Pm005343.1:exon1:c.C434T:p.A145V | PF13943:WPP domain |
| Chr2_10549917 | 2 | Pm005347 | days_1st_flower&days_10st_flower&days_5pct | days_1st_flower&days_10st_flower&days_5pct | downstream | . | . | PF00067:Cytochrome P450 |
| Chr2_10561984 | 2 | Pm005349 | days_1st_flower | days_1st_flower | exonic | nonsynonymous | Pm005349:Pm005349.1:exon1:c.G143C:p.G48A | PF00134:Cyclin, N-terminal domain\|PF02984:Cyclin, C-terminal domain |
| Chr2_10659986 | 2 | Pm005363 | days_1st_flower | days_1st_flower | intergenic | . | . | - |
| Chr2_10745029 | 2 | Pm005387 | days_1st_flower | days_1st_flower | intronic | . | . | - |
| Chr2_10816594 | 2 | Pm005397 | days_1st_flower | days_1st_flower | upstream;downstream | . | . | PF00122:E1-E2 ATPase\|PF12710:haloacid dehalogenase-like hydrolase\|PF12515:Ca2+-ATPase N terminal autoinhibitory domain\|PF00689:Cation transporting ATPase, C-terminus\|PF00690:Cation transporter/ATPase, N-terminus |
| Chr2_10834043 | 2 | Pm005401 | days_1st_flower | days_1st_flower | upstream | . | . | PF01459:Eukaryotic porin |
| Chr2_10870728 | 2 | Pm005409 | days_1st_flower&days_10st_flower&days_5pct | days_1st_flower&days_10st_flower&days_5pct | upstream | . | . | - |
| Chr2_10927934 | 2 | Pm005419 | days_1st_flower&days_10st_flower&days_5pct | days_1st_flower&days_10st_flower&days_5pct | intronic | . | . | PF00689:Cation transporting ATPase, C-terminus\|PF00690:Cation transporter/ATPase, N-terminus\|PF00702:haloacid dehalogenase-like hydrolase\|PF00122:E1-E2 ATPase |
| Chr2_11076929 | 2 | Pm005447 | days_1st_flower | days_1st_flower | exonic | synonymous | Pm005447:Pm005447.1:exon3:c.C195T:p.I65I | - |
| Chr2_11106201 | 2 | Pm005451 | days_1st_flower&days_10st_flower&days_5pct&days_75pct | days_1st_flower&days_10st_flower&days_5pct&days_75pct | exonic | nonsynonymous | Pm005451:Pm005451.1:exon2:c.C540A:p.H180Q | PF00249:Myb-like DNA-binding domain |
| Chr2_11140308 | 2 | Pm005455 | days_1st_flower | days_1st_flower | intergenic | . | . | PF02183:Homeobox associated leucine zipper\|PF00046:Homeobox domain |
| Chr2_11151802 | 2 | Pm005457 | days_1st_flower&days_10st_flower | days_1st_flower&days_10st_flower | intergenic | . | . | PF00331:Glycosyl hydrolase family 10\|PF02018:Carbohydrate binding domain |
| Chr2_11167729 | 2 | Pm005459 | days_1st_flower&days_10st_flower&days_5pct | days_1st_flower&days_10st_flower&days_5pct | downstream | . | . | - |
| Chr2_11184209 | 2 | Pm005462 | days_10st_flower | days_10st_flower | upstream | . | . | PF01417:ENTH domain |
| Chr2_11184364 | 2 | Pm005462 | days_1st_flower | days_1st_flower | upstream | . | . | PF01417:ENTH domain |
| Chr2_11356206 | 2 | Pm005500 | days_1st_flower | days_1st_flower | exonic | nonsynonymous | Pm005500:Pm005500.1:exon11:c.T2750C:p.V917A | PF07714:Protein tyrosine kinase\|PF06799:Protein of unknown function (DUF1230)\|PF14381:Ethylene-responsive protein kinase Le-CTR1 |
| Chr2_11356226 | 2 | Pm005500 | days_1st_flower | days_1st_flower | exonic | synonymous | Pm005500:Pm005500.1:exon11:c.T2730G:p.P910P | PF07714:Protein tyrosine kinase\|PF06799:Protein of unknown function (DUF1230)\|PF14381:Ethylene-responsive protein kinase Le-CTR1 |
| Chr2_11356328 | 2 | Pm005500 | days_1st_flower&days_10st_flower | days_1st_flower&days_10st_flower | exonic | synonymous | Pm005500:Pm005500.1:exon11:c.T2628C:p.L876L | PF07714:Protein tyrosine kinase\|PF06799:Protein of unknown function (DUF1230)\|PF14381:Ethylene-responsive protein kinase Le-CTR1 |
| Chr2_11394238 | 2 | Pm005506 | days_1st_flower | days_1st_flower | intronic | . | . | PF01582:TIR domain\|PF00931:NB-ARC domain |
| Chr2_11443218 | 2 | Pm005517 | days_1st_flower | days_1st_flower | intronic | . | . | - |
| Chr2_11522033 | 2 | Pm005531 | days_1st_flower | days_1st_flower | intergenic | . | . | - |
| Chr2_11522111 | 2 | Pm005531 | days_1st_flower | days_1st_flower | intergenic | . | . | - |
| Chr2_11566026 | 2 | Pm005541 | days_1st_flower | days_1st_flower | intergenic | . | . | PF00153:Mitochondrial carrier protein\|PF00069:Protein kinase domain |
| Chr2_11647304 | 2 | Pm005558 | days_1st_flower | days_1st_flower | upstream | . | . | PF02042:RWP-RK domain |
| Chr2_11647751 | 2 | Pm005559 | days_1st_flower&days_10st_flower | days_1st_flower&days_10st_flower | exonic | nonsynonymous | Pm005559:Pm005559.1:exon1:c.G244C:p.A82P | PF00795:Carbon-nitrogen hydrolase |
| Chr2_11702348 | 2 | Pm005571 | days_1st_flower | days_1st_flower | intergenic | . | . | PF00249:Myb-like DNA-binding domain |
| Chr2_11749657 | 2 | Pm005580 | days_1st_flower | days_1st_flower | upstream | . | . | PF01565:FAD binding domain\|PF09265:Cytokinin dehydrogenase 1, FAD and cytokinin binding |
| Chr2_11782200 | 2 | Pm005586 | days_1st_flower | days_1st_flower | intergenic | . | . | PF00403:Heavy-metal-associated domain\|PF00702:haloacid dehalogenase-like hydrolase\|PF00122:E1-E2 ATPase |
| Chr2_11782209 | 2 | Pm005586 | days_1st_flower | days_1st_flower | intergenic | . | . | PF00403:Heavy-metal-associated domain\|PF00702:haloacid dehalogenase-like hydrolase\|PF00122:E1-E2 ATPase |
| Chr2_11835895 | 2 | Pm005593 | days_1st_flower | days_1st_flower | exonic | synonymous | Pm005593:Pm005593.1:exon1:c.T336G:p.A112A | PF00561:alpha/beta hydrolase fold |
| Chr2_11924559 | 2 | Pm005612 | days_1st_flower | days_1st_flower | upstream;downstream | . | . | PF08541:3-Oxoacyl-[acyl-carrier-protein (ACP)] synthase III C terminal\|PF08392:FAE1/Type III polyketide synthase-like protein |
| Chr2_12110156 | 2 | Pm005637 | days_1st_flower | days_1st_flower | intergenic | . | . | PF01722:BolA-like protein |
| Chr2_12498159 | 2 | Pm005706 | days_1st_flower | days_1st_flower | intergenic | . | . | PF04857:CAF1 family ribonuclease |
| Chr2_12541186 | 2 | Pm005712 | days_1st_flower&days_5pct | days_1st_flower&days_5pct | intergenic | . | . | - |
| Chr2_12594310 | 2 | Pm005725 | days_1st_flower&days_10st_flower | days_1st_flower&days_10st_flower | intronic | . | . | PF04130:Spc97 / Spc98 family |
| Chr2_12594311 | 2 | Pm005725 | days_1st_flower&days_10st_flower | days_1st_flower&days_10st_flower | intronic | . | . | PF04130:Spc97 / Spc98 family |
| Chr2_12594312 | 2 | Pm005725 | days_1st_flower&days_10st_flower | days_1st_flower&days_10st_flower | intronic | . | . | PF04130:Spc97 / Spc98 family |
| Chr2_12594313 | 2 | Pm005725 | days_1st_flower&days_10st_flower | days_1st_flower&days_10st_flower | intronic | . | . | PF04130:Spc97 / Spc98 family |
| Chr2_12670861 | 2 | Pm005741 | days_1st_flower | days_1st_flower | intronic | . | . | PF02701:Dof domain, zinc finger |
| Chr2_12670873 | 2 | Pm005741 | days_1st_flower | days_1st_flower | intronic | . | . | PF02701:Dof domain, zinc finger |
| Chr2_12690293 | 2 | Pm005743 | days_1st_flower&days_10st_flower | days_1st_flower&days_10st_flower | intergenic | . | . | - |
| Chr2_12822172 | 2 | Pm005765 | days_1st_flower | days_1st_flower | intronic | . | . | PF09763:Exocyst complex component Sec3\|PF15277:Exocyst complex component SEC3 N-terminal PIP2 binding PH |
| Chr2_12826141 | 2 | Pm005765 | days_1st_flower | days_1st_flower | exonic | synonymous | Pm005765:Pm005765.1:exon7:c.G522A:p.E174E | PF09763:Exocyst complex component Sec3\|PF15277:Exocyst complex component SEC3 N-terminal PIP2 binding PH |
| Chr2_12863230 | 2 |  | days_1st_flower&days_10st_flower | days_1st_flower&days_10st_flower | intergenic | . | . |  |
| Chr2_12926711 | 2 | Pm005773 | days_1st_flower | days_1st_flower | intergenic | . | . | PF13499:EF-hand domain pair\|PF13833:EF-hand domain pair |
| Chr2_12930614 | 2 | Pm005774 | days_1st_flower&days_10st_flower | days_1st_flower&days_10st_flower | exonic | synonymous | Pm005774:Pm005774.1:exon1:c.C315T:p.G105G | PF01501:Glycosyl transferase family 8 |
| Chr2_12930695 | 2 | Pm005774 | days_1st_flower | days_1st_flower | exonic | synonymous | Pm005774:Pm005774.1:exon1:c.G234A:p.E78E | PF01501:Glycosyl transferase family 8 |
| Chr2_12988342 | 2 | Pm005783 | days_1st_flower | days_1st_flower | downstream | . | . | PF02365:No apical meristem (NAM) protein |
| Chr2_13009441 | 2 | Pm005787 | days_10st_flower | days_10st_flower | exonic | synonymous | Pm005787:Pm005787.1:exon1:c.T102C:p.L34L | PF00201:UDP-glucoronosyl and UDP-glucosyl transferase |
| Chr2_13270205 | 2 | Pm005842 | days_1st_flower | days_1st_flower | intergenic | . | . | - |
| Chr2_13290101 | 2 | Pm005846 | days_1st_flower | days_1st_flower | intergenic | . | . | PF00227:Proteasome subunit\|PF10584:Proteasome subunit A N-terminal signature |
| Chr2_13313463 | 2 | Pm005849 | days_10st_flower | days_10st_flower | intergenic | . | . | PF01370:NAD dependent epimerase/dehydratase family |
| Chr2_13334994 | 2 | Pm005852 | days_25pct | days_25pct | upstream | . | . | PF01197:Ribosomal protein L31 |
| Chr2_13346670 | 2 | Pm005855 | days_1st_flower&days_10st_flower | days_1st_flower&days_10st_flower | intronic | . | . | PF00067:Cytochrome P450\|PF03492:SAM dependent carboxyl methyltransferase |
| Chr2_13580457 | 2 | Pm005897 | days_1st_flower | days_1st_flower | exonic | synonymous | Pm005897:Pm005897.1:exon1:c.G78C:p.L26L | PF01453:D-mannose binding lectin\|PF00954:S-locus glycoprotein domain\|PF00069:Protein kinase domain |
| Chr2_13619580 | 2 | Pm005902 | days_10st_flower&days_5pct | days_10st_flower&days_5pct | intergenic | . | . | - |
| Chr2_13637360 | 2 | Pm005905 | days_10st_flower&days_5pct | days_10st_flower&days_5pct | upstream | . | . | PF03188:Eukaryotic cytochrome b561 |
| Chr2_13739313 | 2 | Pm005926 | days_1st_flower | days_1st_flower | intergenic | . | . | - |
| Chr2_14054888 | 2 | Pm005978 | days_1st_flower | days_1st_flower | upstream | . | . | PF00610:Domain found in Dishevelled, Egl-10, and Pleckstrin (DEP)\|PF00462:Glutaredoxin\|PF04784:Protein of unknown function, DUF547 |
| Chr2_14664580 | 2 | Pm006075 | days_1st_flower&days_10st_flower | days_1st_flower&days_10st_flower | exonic | nonsynonymous | Pm006075:Pm006075.1:exon1:c.T30G:p.I10M | PF05623:Protein of unknown function (DUF789) |
| Chr2_14672411 | 2 | Pm006075 | days_1st_flower | days_1st_flower | intronic | . | . | PF05623:Protein of unknown function (DUF789) |
| Chr2_14800725 | 2 | Pm006092 | days_1st_flower | days_1st_flower | exonic | synonymous | Pm006092:Pm006092.1:exon2:c.A450G:p.K150K | PF03171:2OG-Fe(II) oxygenase superfamily\|PF14226:non-haem dioxygenase in morphine synthesis N-terminal |
| Chr2_15075821 | 2 | Pm006135 | days_75pct | days_75pct | intergenic | . | . | PF03478:Protein of unknown function (DUF295) |
| Chr2_15148117 | 2 | Pm006154 | days_1st_flower&days_10st_flower | days_1st_flower&days_10st_flower | intergenic | . | . | PF13499:EF-hand domain pair\|PF00069:Protein kinase domain |
| Chr2_15211353 | 2 | Pm006167 | days_1st_flower&days_10st_flower&days_5pct | days_1st_flower&days_10st_flower&days_5pct | exonic | nonsynonymous | Pm006167:Pm006167.1:exon2:c.T388G:p.Y130D | PF02225:PA domain\|PF05922:Peptidase inhibitor I9\|PF00082:Subtilase family |
| Chr2_15284019 | 2 | Pm006180 | days_1st_flower&days_10st_flower&days_5pct | days_1st_flower&days_10st_flower&days_5pct | intronic | . | . | PF03635:Vacuolar protein sorting-associated protein 35 |
| Chr2_15329415 | 2 | Pm006187 | days_1st_flower | days_1st_flower | intergenic | . | . | PF00225:Kinesin motor domain |
| Chr2_15443239 | 2 | Pm006208 | days_1st_flower&days_10st_flower&days_5pct | days_1st_flower&days_10st_flower&days_5pct | upstream | . | . | PF12899:Alkaline and neutral invertase |
| Chr2_15473055 | 2 | Pm006214 | days_1st_flower | days_1st_flower | intronic | . | . | - |
| Chr2_15473056 | 2 | Pm006214 | days_1st_flower | days_1st_flower | intronic | . | . | - |
| Chr2_15532231 | 2 | Pm006225 | days_1st_flower | days_1st_flower | intronic | . | . | PF02319:E2F/DP family winged-helix DNA-binding domain\|PF16421:E2F transcription factor CC-MB domain |
| Chr2_15532233 | 2 | Pm006225 | days_1st_flower | days_1st_flower | intronic | . | . | PF02319:E2F/DP family winged-helix DNA-binding domain\|PF16421:E2F transcription factor CC-MB domain |
| Chr2_15535804 | 2 | Pm006226 | days_1st_flower | days_1st_flower | intergenic | . | . | PF03134:TB2/DP1, HVA22 family |
| Chr2_15535877 | 2 | Pm006226 | days_1st_flower&days_10st_flower | days_1st_flower&days_10st_flower | intergenic | . | . | PF03134:TB2/DP1, HVA22 family |
| Chr2_15535878 | 2 | Pm006226 | days_1st_flower&days_10st_flower | days_1st_flower&days_10st_flower | intergenic | . | . | PF03134:TB2/DP1, HVA22 family |
| Chr2_15540219 | 2 | Pm006227 | days_1st_flower | days_1st_flower | exonic | nonsynonymous | Pm006227:Pm006227.1:exon1:c.T79A:p.S27T | - |
| Chr2_15558843 | 2 | Pm006231 | days_1st_flower&days_10st_flower&days_5pct | days_1st_flower&days_10st_flower&days_5pct | intergenic | . | . | PF03641:Possible lysine decarboxylase |
| Chr2_15580255 | 2 | Pm006233 | days_1st_flower | days_1st_flower | upstream | . | . | PF01747:ATP-sulfurylase\|PF14306:PUA-like domain |
| Chr2_15665239 | 2 | Pm006244 | days_1st_flower&days_10st_flower | days_1st_flower&days_10st_flower | upstream | . | . | PF00415:Regulator of chromosome condensation (RCC1) repeat |
| Chr2_15686278 | 2 | Pm006249 | days_1st_flower | days_1st_flower | exonic | synonymous | Pm006249:Pm006249.1:exon1:c.A324C:p.T108T | PF07250:Glyoxal oxidase N-terminus\|PF09118:Domain of unknown function (DUF1929) |
| Chr2_15702629 | 2 | Pm006252 | days_1st_flower | days_1st_flower | intergenic | . | . | PF07250:Glyoxal oxidase N-terminus\|PF09118:Domain of unknown function (DUF1929) |
| Chr2_15786760 | 2 | Pm006264 | days_10st_flower | days_10st_flower | intergenic | . | . | - |
| Chr2_15863229 | 2 | Pm006280 | days_1st_flower | days_1st_flower | upstream | . | . | PF00085:Thioredoxin |
| Chr2_15908519 | 2 | Pm006288 | days_1st_flower&days_10st_flower&days_5pct | days_1st_flower&days_10st_flower&days_5pct | intronic | . | . | PF00590:Tetrapyrrole (Corrin/Porphyrin) Methylases |
| Chr2_16198178 | 2 | Pm006331 | days_1st_flower | days_1st_flower | intronic | . | . | PF12755:Vacuolar 14 Fab1-binding region\|PF03177:Non-repetitive/WGA-negative nucleoporin C-terminal\|PF11916:Vacuolar protein 14 C-terminal Fig4p binding\|PF08801:Nup133 N terminal like |
| Chr2_16272624 | 2 | Pm006339 | days_10st_flower | days_10st_flower | intergenic | . | . | PF00293:NUDIX domain |
| Chr2_16272630 | 2 | Pm006340 | days_10st_flower | days_10st_flower | intergenic | . | . | - |
| Chr2_16476351 | 2 | Pm006368 | days_10st_flower | days_10st_flower | intergenic | . | . | PF03478:Protein of unknown function (DUF295) |
| Chr2_16715397 | 2 | Pm006407 | days_1st_flower&days_10st_flower | days_1st_flower&days_10st_flower | intronic | . | . | PF00343:Carbohydrate phosphorylase |
| Chr2_16862494 | 2 | Pm006442 | days_1st_flower&days_10st_flower | days_1st_flower&days_10st_flower | upstream | . | . | - |
| Chr2_16890077 | 2 | Pm006446 | days_1st_flower&days_10st_flower | days_1st_flower&days_10st_flower | intronic | . | . | PF02714:Calcium-dependent channel, 7TM region, putative phosphate\|PF13967:Late exocytosis, associated with Golgi transport\|PF14703:Cytosolic domain of 10TM putative phosphate transporter |
| Chr2_16915931 | 2 | Pm006455 | days_1st_flower&days_10st_flower | days_1st_flower&days_10st_flower | intronic | . | . | PF10250:GDP-fucose protein O-fucosyltransferase |
| Chr2_17157810 | 2 | Pm006496 | days_1st_flower | days_1st_flower | intergenic | . | . | - |
| Chr2_17178778 | 2 | Pm006501 | days_1st_flower&days_10st_flower | days_1st_flower&days_10st_flower | intergenic | . | . | PF12906:RING-variant domain\|PF12428:Protein of unknown function (DUF3675) |
| Chr2_17374293 | 2 | Pm006532 | days_1st_flower&days_10st_flower | days_1st_flower&days_10st_flower | intergenic | . | . | PF13921:Myb-like DNA-binding domain |
| Chr2_17668266 | 2 | Pm006575 | days_1st_flower | days_1st_flower | exonic | synonymous | Pm006575:Pm006575.1:exon1:c.T1131G:p.A377A | PF07250:Glyoxal oxidase N-terminus |
| Chr2_17856598 | 2 | Pm006602 | days_10st_flower | days_10st_flower | intergenic | . | . | PF13041:PPR repeat family |
| Chr2_18095493 | 2 | Pm006639 | days_1st_flower&days_10st_flower | days_1st_flower&days_10st_flower | downstream | . | . | PF02358:Trehalose-phosphatase\|PF00982:Glycosyltransferase family 20 |
| Chr2_18216553 | 2 | Pm006657 | days_1st_flower&days_10st_flower | days_1st_flower&days_10st_flower | intergenic | . | . | - |
| Chr2_18557507 | 2 | Pm006705 | days_1st_flower | days_1st_flower | exonic | nonsynonymous | Pm006705:Pm006705.1:exon1:c.G83A:p.R28K | - |
| Chr2_18637274 | 2 | Pm006717 | days_10st_flower | days_10st_flower | intergenic | . | . | PF03790:KNOX1 domain\|PF03789:ELK domain\|PF03791:KNOX2 domain\|PF05920:Homeobox KN domain |
| Chr2_18899794 | 2 | Pm006760 | days_1st_flower&days_10st_flower | days_1st_flower&days_10st_flower | downstream | . | . | PF00248:Aldo/keto reductase family |
| Chr2_18899806 | 2 | Pm006760 | days_1st_flower&days_10st_flower | days_1st_flower&days_10st_flower | downstream | . | . | PF00248:Aldo/keto reductase family |
| Chr2_19255324 | 2 | Pm006804 | days_1st_flower&days_10st_flower | days_1st_flower&days_10st_flower | intronic | . | . | PF00005:ABC transporter |
| Chr2_19330971 | 2 | Pm006812 | days_1st_flower&days_10st_flower | days_1st_flower&days_10st_flower | downstream | . | . | PF00022:Actin |
| Chr2_19455526 | 2 | Pm006834 | days_1st_flower&days_10st_flower&days_5pct | days_1st_flower&days_10st_flower&days_5pct | downstream | . | . | - |
| Chr2_19460526 | 2 | Pm006835 | days_10st_flower | days_10st_flower | exonic | nonsynonymous | Pm006835:Pm006835.1:exon6:c.A1376C:p.D459A | PF12819:Carbohydrate-binding protein of the ER\|PF07714:Protein tyrosine kinase |
| Chr2_19464285 | 2 | Pm006835 | days_10st_flower&days_5pct | days_10st_flower&days_5pct | intergenic | . | . | PF12819:Carbohydrate-binding protein of the ER\|PF07714:Protein tyrosine kinase |
| Chr2_19829163 | 2 | Pm006889 | days_10st_flower&days_5pct | days_10st_flower&days_5pct | intergenic | . | . | PF08492:SRP72 RNA-binding domain\|PF17004:Putative TPR-like repeat |
| Chr2_19952824 | 2 | Pm006907 | days_1st_flower&days_10st_flower | days_1st_flower&days_10st_flower | exonic | synonymous | Pm006907:Pm006907.1:exon1:c.C393A:p.T131T | PF03763:Remorin, C-terminal region |
| Chr2_20037474 | 2 | Pm006915 | days_1st_flower | days_1st_flower | intergenic | . | . | PF12796:Ankyrin repeats (3 copies) |
| Chr2_20239657 | 2 | Pm006936 | days_1st_flower | days_1st_flower | intergenic | . | . | PF00067:Cytochrome P450 |
| Chr2_20499774 | 2 | Pm006974 | days_75pct | days_75pct | upstream | . | . | - |
| Chr2_20804425 | 2 | Pm007011 | days_10st_flower | days_10st_flower | intergenic | . | . | PF01753:MYND finger |
| Chr2_21038800 | 2 | Pm007045 | days_10st_flower | days_10st_flower | upstream | . | . | PF00282:Pyridoxal-dependent decarboxylase conserved domain |
| Chr2_21038992 | 2 | Pm007045 | days_1st_flower&days_10st_flower | days_1st_flower&days_10st_flower | upstream | . | . | PF00282:Pyridoxal-dependent decarboxylase conserved domain |
| Chr2_21144493 | 2 | Pm007061 | days_75pct | days_75pct | intergenic | . | . | PF02519:Auxin responsive protein |
| Chr2_21177243 | 2 | Pm007066 | days_1st_flower&days_10st_flower | days_1st_flower&days_10st_flower | intergenic | . | . | PF00069:Protein kinase domain |
| Chr2_21183825 | 2 | Pm007067 | days_1st_flower&days_10st_flower | days_1st_flower&days_10st_flower | downstream | . | . | PF03083:Sugar efflux transporter for intercellular exchange |
| Chr2_21200879 | 2 | Pm007069 | days_10st_flower&days_5pct | days_10st_flower&days_5pct | intergenic | . | . | PF03009:Glycerophosphoryl diester phosphodiesterase family |
| Chr2_22608897 | 2 | Pm007232 | days_10st_flower | days_10st_flower | intronic | . | . | PF05033:Pre-SET motif\|PF00856:SET domain\|PF02182:SAD/SRA domain |
| Chr2_23514162 | 2 | Pm007340 | days_1st_flower&days_10st_flower&days_5pct&days_25pct | days_1st_flower&days_10st_flower&days_5pct&days_25pct | intergenic | . | . | - |
| Chr2_24668713 | 2 | Pm007423 | days_10st_flower | days_10st_flower | downstream | . | . | - |
| Chr2_27104682 | 2 | Pm007662 | days_75pct | days_75pct | upstream | . | . | - |
| Chr2_27109509 | 2 | Pm007663 | days_1st_flower&days_5pct&days_25pct&days_75pct | days_1st_flower&days_5pct&days_25pct&days_75pct | downstream | . | . | - |
| Chr2_2984548 | 2 | Pm004167 | days_1st_flower | days_1st_flower | intronic | . | . | PF01582:TIR domain\|PF00931:NB-ARC domain |
| Chr2_3168338 | 2 | Pm004197 | days_1st_flower&days_10st_flower | days_1st_flower&days_10st_flower | upstream | . | . | - |
| Chr2_3168897 | 2 | Pm004197 | days_1st_flower&days_10st_flower | days_1st_flower&days_10st_flower | upstream | . | . | - |
| Chr2_3173141 | 2 | Pm004198 | days_1st_flower | days_1st_flower | intronic | . | . | PF10250:GDP-fucose protein O-fucosyltransferase |
| Chr2_3173275 | 2 | Pm004198 | days_5pct | days_5pct | intronic | . | . | PF10250:GDP-fucose protein O-fucosyltransferase |
| Chr2_3173281 | 2 | Pm004198 | days_5pct | days_5pct | intronic | . | . | PF10250:GDP-fucose protein O-fucosyltransferase |
| Chr2_3176101 | 2 | Pm004198 | days_1st_flower&days_10st_flower | days_1st_flower&days_10st_flower | intronic | . | . | PF10250:GDP-fucose protein O-fucosyltransferase |
| Chr2_3176114 | 2 | Pm004198 | days_1st_flower&days_10st_flower | days_1st_flower&days_10st_flower | intronic | . | . | PF10250:GDP-fucose protein O-fucosyltransferase |
| Chr2_3176465 | 2 | Pm004198 | days_1st_flower&days_10st_flower | days_1st_flower&days_10st_flower | intronic | . | . | PF10250:GDP-fucose protein O-fucosyltransferase |
| Chr2_3217897 | 2 | Pm004202 | days_1st_flower | days_1st_flower | exonic | nonsynonymous | Pm004202:Pm004202.1:exon1:c.A657T:p.K219N | PF13041:PPR repeat family\|PF01535:PPR repeat |
| Chr2_3217927 | 2 | Pm004202 | days_1st_flower | days_1st_flower | exonic | synonymous | Pm004202:Pm004202.1:exon1:c.A687G:p.V229V | PF13041:PPR repeat family\|PF01535:PPR repeat |
| Chr2_3218071 | 2 | Pm004202 | days_1st_flower | days_1st_flower | exonic | synonymous | Pm004202:Pm004202.1:exon1:c.C831A:p.A277A | PF13041:PPR repeat family\|PF01535:PPR repeat |
| Chr2_32892035 | 2 | Pm008398 | days_1st_flower | days_1st_flower | intergenic | . | . | PF01501:Glycosyl transferase family 8 |
| Chr2_3318203 | 2 | Pm004221 | days_1st_flower&days_10st_flower | days_1st_flower&days_10st_flower | intronic | . | . | PF00647:Elongation factor 1 gamma, conserved domain\|PF00043:Glutathione S-transferase, C-terminal domain\|PF02798:Glutathione S-transferase, N-terminal domain |
| Chr2_3318219 | 2 | Pm004221 | days_1st_flower&days_10st_flower | days_1st_flower&days_10st_flower | intronic | . | . | PF00647:Elongation factor 1 gamma, conserved domain\|PF00043:Glutathione S-transferase, C-terminal domain\|PF02798:Glutathione S-transferase, N-terminal domain |
| Chr2_3318606 | 2 | Pm004221 | days_1st_flower&days_10st_flower&days_5pct | days_1st_flower&days_10st_flower&days_5pct | intronic | . | . | PF00647:Elongation factor 1 gamma, conserved domain\|PF00043:Glutathione S-transferase, C-terminal domain\|PF02798:Glutathione S-transferase, N-terminal domain |
| Chr2_3326885 | 2 | Pm004223 | days_1st_flower&days_10st_flower&days_5pct | days_1st_flower&days_10st_flower&days_5pct | upstream | . | . | - |
| Chr2_3336555 | 2 | Pm004226 | days_1st_flower&days_10st_flower | days_1st_flower&days_10st_flower | intronic | . | . | PF00724:NADH:flavin oxidoreductase / NADH oxidase family |
| Chr2_3337148 | 2 | Pm004226 | days_1st_flower&days_10st_flower | days_1st_flower&days_10st_flower | intronic | . | . | PF00724:NADH:flavin oxidoreductase / NADH oxidase family |
| Chr2_3474906 | 2 | Pm004245 | days_5pct | days_5pct | intronic | . | . | PF00657:GDSL-like Lipase/Acylhydrolase |
| Chr2_3517525 | 2 | Pm004252 | days_1st_flower&days_5pct | days_1st_flower&days_5pct | intronic | . | . | - |
| Chr2_3522127 | 2 | Pm004253 | days_5pct | days_5pct | intronic | . | . | - |
| Chr2_3525837 | 2 | Pm004253 | days_5pct | days_5pct | upstream | . | . | - |
| Chr2_35548889 | 2 | Pm008734 | days_10st_flower | days_10st_flower | upstream | . | . | PF00319:SRF-type transcription factor (DNA-binding and dimerisation domain) |
| Chr2_3590985 | 2 | Pm004259 | days_1st_flower&days_10st_flower | days_1st_flower&days_10st_flower | intronic | . | . | PF02622:Uncharacterized ACR, COG1678 |
| Chr2_3604310 | 2 | Pm004261 | days_1st_flower&days_10st_flower | days_1st_flower&days_10st_flower | exonic | nonsynonymous | Pm004261:Pm004261.1:exon1:c.A26G:p.N9S | PF00520:Ion transport protein |
| Chr2_3638239 | 2 | Pm004267 | days_1st_flower&days_10st_flower&days_5pct&days_25pct&days_75pct | days_1st_flower&days_10st_flower&days_5pct&days_25pct&days_75pct | exonic | synonymous | Pm004267:Pm004267.1:exon4:c.T1071C:p.H357H | PF13962:Domain of unknown function\|PF12796:Ankyrin repeats (3 copies) |
| Chr2_3641580 | 2 | Pm004267 | days_1st_flower&days_10st_flower&days_5pct | days_1st_flower&days_10st_flower&days_5pct | intergenic | . | . | PF13962:Domain of unknown function\|PF12796:Ankyrin repeats (3 copies) |
| Chr2_3642097 | 2 | Pm004268 | days_10st_flower | days_10st_flower | intergenic | . | . | PF12680:SnoaL-like domain |
| Chr2_3649806 | 2 | Pm004268 | days_1st_flower&days_10st_flower | days_1st_flower&days_10st_flower | intergenic | . | . | PF12680:SnoaL-like domain |
| Chr2_3665394 | 2 | Pm004271 | days_1st_flower | days_1st_flower | downstream | . | . | PF01554:MatE |
| Chr2_3667145 | 2 | Pm004271 | days_10st_flower | days_10st_flower | intronic | . | . | PF01554:MatE |
| Chr2_3668473 | 2 | Pm004271 | days_10st_flower | days_10st_flower | exonic | synonymous | Pm004271:Pm004271.1:exon1:c.A54T:p.G18G | PF01554:MatE |
| Chr2_3691538 | 2 | Pm004274 | days_1st_flower&days_10st_flower&days_5pct&days_25pct&days_75pct | days_1st_flower&days_10st_flower&days_5pct&days_25pct&days_75pct | intronic | . | . | PF00931:NB-ARC domain\|PF13855:Leucine rich repeat |
| Chr2_3798513 | 2 | Pm004290 | days_1st_flower&days_10st_flower&days_5pct | days_1st_flower&days_10st_flower&days_5pct | intergenic | . | . | PF06219:Protein of unknown function (DUF1005) |
| Chr2_3832292 | 2 | Pm004299 | days_10st_flower | days_10st_flower | upstream | . | . | PF05770:Inositol 1, 3, 4-trisphosphate 5/6-kinase |
| Chr2_3835774 | 2 | Pm004299 | days_5pct | days_5pct | intergenic | . | . | PF05770:Inositol 1, 3, 4-trisphosphate 5/6-kinase |
| Chr2_38413477 | 2 | Pm009081 | days_10st_flower | days_10st_flower | intergenic | . | . | PF02736:Myosin N-terminal SH3-like domain\|PF01843:DIL domain\|PF00063:Myosin head (motor domain) |
| Chr2_38439047 | 2 | Pm009082 | days_10st_flower | days_10st_flower | intergenic | . | . | PF00743:Flavin-binding monooxygenase-like |
| Chr2_3878000 | 2 | Pm004303 | days_1st_flower&days_10st_flower | days_1st_flower&days_10st_flower | intronic | . | . | PF12394:Protein of unknown function (DUF3657)\|PF05057:Putative serine esterase (DUF676) |
| Chr2_3880817 | 2 | Pm004303 | days_1st_flower&days_10st_flower | days_1st_flower&days_10st_flower | exonic | synonymous | Pm004303:Pm004303.1:exon11:c.G1572A:p.K524K | PF12394:Protein of unknown function (DUF3657)\|PF05057:Putative serine esterase (DUF676) |
| Chr2_3881025 | 2 | Pm004303 | days_1st_flower | days_1st_flower | intronic | . | . | PF12394:Protein of unknown function (DUF3657)\|PF05057:Putative serine esterase (DUF676) |
| Chr2_3881032 | 2 | Pm004303 | days_1st_flower | days_1st_flower | intronic | . | . | PF12394:Protein of unknown function (DUF3657)\|PF05057:Putative serine esterase (DUF676) |
| Chr2_3881068 | 2 | Pm004303 | days_1st_flower | days_1st_flower | intronic | . | . | PF12394:Protein of unknown function (DUF3657)\|PF05057:Putative serine esterase (DUF676) |
| Chr2_3884040 | 2 | Pm004303 | days_1st_flower | days_1st_flower | downstream | . | . | PF12394:Protein of unknown function (DUF3657)\|PF05057:Putative serine esterase (DUF676) |
| Chr2_3884179 | 2 | Pm004303 | days_5pct | days_5pct | downstream | . | . | PF12394:Protein of unknown function (DUF3657)\|PF05057:Putative serine esterase (DUF676) |
| Chr2_38955730 | 2 | Pm009134 | days_1st_flower&days_10st_flower&days_5pct&days_25pct&days_75pct | days_1st_flower&days_10st_flower&days_5pct&days_25pct&days_75pct | intergenic | . | . | - |
| Chr2_3907124 | 2 | Pm004307 | days_1st_flower | days_1st_flower | upstream | . | . | PF03953:Tubulin C-terminal domain\|PF00091:Tubulin/FtsZ family, GTPase domain |
| Chr2_3910451 | 2 | Pm004308 | days_1st_flower | days_1st_flower | intergenic | . | . | - |
| Chr2_3915491 | 2 | Pm004309 | days_10st_flower&days_5pct&days_25pct | days_10st_flower&days_5pct&days_25pct | upstream | . | . | - |
| Chr2_3916215 | 2 | Pm004309 | days_5pct | days_5pct | upstream | . | . | - |
| Chr2_3923691 | 2 | Pm004310 | days_10st_flower | days_10st_flower | exonic | synonymous | Pm004310:Pm004310.1:exon1:c.C2409T:p.A803A | PF08263:Leucine rich repeat N-terminal domain\|PF00069:Protein kinase domain\|PF13855:Leucine rich repeat |
| Chr2_3993005 | 2 | Pm004318 | days_10st_flower | days_10st_flower | intergenic | . | . | PF14303:No apical meristem-associated C-terminal domain |
| Chr2_4000133 | 2 | Pm004319 | days_10st_flower | days_10st_flower | upstream | . | . | PF03360:Glycosyltransferase family 43 |
| Chr2_4000475 | 2 | Pm004319 | days_10st_flower | days_10st_flower | exonic | synonymous | Pm004319:Pm004319.1:exon1:c.A282G:p.L94L | PF03360:Glycosyltransferase family 43 |
| Chr2_4000861 | 2 | Pm004319 | days_10st_flower | days_10st_flower | exonic | nonsynonymous | Pm004319:Pm004319.1:exon1:c.T668C:p.L223S | PF03360:Glycosyltransferase family 43 |
| Chr2_4001102 | 2 | Pm004319 | days_10st_flower | days_10st_flower | intronic | . | . | PF03360:Glycosyltransferase family 43 |
| Chr2_4001435 | 2 | Pm004319 | days_10st_flower | days_10st_flower | intronic | . | . | PF03360:Glycosyltransferase family 43 |
| Chr2_4001494 | 2 | Pm004319 | days_10st_flower | days_10st_flower | intronic | . | . | PF03360:Glycosyltransferase family 43 |
| Chr2_4001802 | 2 | Pm004319 | days_10st_flower | days_10st_flower | intronic | . | . | PF03360:Glycosyltransferase family 43 |
| Chr2_4004078 | 2 | Pm004320 | days_10st_flower | days_10st_flower | intergenic | . | . | PF12295:Symplekin tight junction protein C terminal\|PF11935:Domain of unknown function (DUF3453) |
| Chr2_4006749 | 2 | Pm004320 | days_10st_flower | days_10st_flower | intronic | . | . | PF12295:Symplekin tight junction protein C terminal\|PF11935:Domain of unknown function (DUF3453) |
| Chr2_4007772 | 2 | Pm004320 | days_10st_flower | days_10st_flower | intronic | . | . | PF12295:Symplekin tight junction protein C terminal\|PF11935:Domain of unknown function (DUF3453) |
| Chr2_4009385 | 2 | Pm004320 | days_10st_flower | days_10st_flower | intronic | . | . | PF12295:Symplekin tight junction protein C terminal\|PF11935:Domain of unknown function (DUF3453) |
| Chr2_4009469 | 2 | Pm004320 | days_10st_flower | days_10st_flower | intronic | . | . | PF12295:Symplekin tight junction protein C terminal\|PF11935:Domain of unknown function (DUF3453) |
| Chr2_4012385 | 2 | Pm004320 | days_10st_flower | days_10st_flower | intronic | . | . | PF12295:Symplekin tight junction protein C terminal\|PF11935:Domain of unknown function (DUF3453) |
| Chr2_4014850 | 2 | Pm004320 | days_10st_flower | days_10st_flower | exonic | nonsynonymous | Pm004320:Pm004320.1:exon4:c.A395C:p.N132T | PF12295:Symplekin tight junction protein C terminal\|PF11935:Domain of unknown function (DUF3453) |
| Chr2_4015894 | 2 | Pm004320 | days_10st_flower | days_10st_flower | intronic | . | . | PF12295:Symplekin tight junction protein C terminal\|PF11935:Domain of unknown function (DUF3453) |
| Chr2_4017958 | 2 | Pm004320 | days_10st_flower | days_10st_flower | intergenic | . | . | PF12295:Symplekin tight junction protein C terminal\|PF11935:Domain of unknown function (DUF3453) |
| Chr2_4019811 | 2 | Pm004321 | days_10st_flower | days_10st_flower | downstream | . | . | PF11935:Domain of unknown function (DUF3453) |
| Chr2_4020670 | 2 | Pm004321 | days_10st_flower | days_10st_flower | downstream | . | . | PF11935:Domain of unknown function (DUF3453) |
| Chr2_4022482 | 2 | Pm004321 | days_10st_flower | days_10st_flower | exonic | synonymous | Pm004321:Pm004321.1:exon4:c.C481T:p.L161L | PF11935:Domain of unknown function (DUF3453) |
| Chr2_4022632 | 2 | Pm004321 | days_10st_flower | days_10st_flower | intronic | . | . | PF11935:Domain of unknown function (DUF3453) |
| Chr2_4024538 | 2 | Pm004321 | days_10st_flower | days_10st_flower | intronic | . | . | PF11935:Domain of unknown function (DUF3453) |
| Chr2_4024632 | 2 | Pm004321 | days_10st_flower | days_10st_flower | intronic | . | . | PF11935:Domain of unknown function (DUF3453) |
| Chr2_4025701 | 2 | Pm004321 | days_1st_flower&days_10st_flower | days_1st_flower&days_10st_flower | upstream | . | . | PF11935:Domain of unknown function (DUF3453) |
| Chr2_4026055 | 2 | Pm004322 | days_10st_flower | days_10st_flower | intronic | . | . | PF02847:MA3 domain\|PF02854:MIF4G domain |
| Chr2_4026444 | 2 | Pm004322 | days_10st_flower | days_10st_flower | exonic | synonymous | Pm004322:Pm004322.1:exon3:c.A414G:p.E138E | PF02847:MA3 domain\|PF02854:MIF4G domain |
| Chr2_4026705 | 2 | Pm004322 | days_10st_flower | days_10st_flower | exonic | nonsynonymous | Pm004322:Pm004322.1:exon3:c.C675G:p.S225R | PF02847:MA3 domain\|PF02854:MIF4G domain |
| Chr2_4026711 | 2 | Pm004322 | days_10st_flower | days_10st_flower | exonic | synonymous | Pm004322:Pm004322.1:exon3:c.G681A:p.K227K | PF02847:MA3 domain\|PF02854:MIF4G domain |
| Chr2_4026997 | 2 | Pm004322 | days_10st_flower | days_10st_flower | exonic | nonsynonymous | Pm004322:Pm004322.1:exon3:c.A967G:p.S323G | PF02847:MA3 domain\|PF02854:MIF4G domain |
| Chr2_4027560 | 2 | Pm004322 | days_10st_flower | days_10st_flower | intronic | . | . | PF02847:MA3 domain\|PF02854:MIF4G domain |
| Chr2_4028599 | 2 | Pm004322 | days_10st_flower | days_10st_flower | intronic | . | . | PF02847:MA3 domain\|PF02854:MIF4G domain |
| Chr2_4029716 | 2 | Pm004322 | days_10st_flower | days_10st_flower | intronic | . | . | PF02847:MA3 domain\|PF02854:MIF4G domain |
| Chr2_4033102 | 2 | Pm004322 | days_10st_flower | days_10st_flower | intronic | . | . | PF02847:MA3 domain\|PF02854:MIF4G domain |
| Chr2_4033454 | 2 | Pm004322 | days_10st_flower | days_10st_flower | exonic | nonsynonymous | Pm004322:Pm004322.1:exon11:c.A2285T:p.Y762F | PF02847:MA3 domain\|PF02854:MIF4G domain |
| Chr2_4061477 | 2 | Pm004327 | days_5pct | days_5pct | intronic | . | . | PF17123:RING-like zinc finger\|PF00092:von Willebrand factor type A domain |
| Chr2_4066187 | 2 | Pm004327 | days_10st_flower | days_10st_flower | intergenic | . | . | PF17123:RING-like zinc finger\|PF00092:von Willebrand factor type A domain |
| Chr2_4075822 | 2 | Pm004328 | days_1st_flower | days_1st_flower | downstream | . | . | PF02309:AUX/IAA family |
| Chr2_4078235 | 2 | Pm004328 | days_1st_flower&days_10st_flower | days_1st_flower&days_10st_flower | exonic | synonymous | Pm004328:Pm004328.1:exon1:c.A591G:p.E197E | PF02309:AUX/IAA family |
| Chr2_4097180 | 2 | Pm004330 | days_25pct | days_25pct | intergenic | . | . | PF00010:Helix-loop-helix DNA-binding domain |
| Chr2_4103151 | 2 | Pm004330 | days_1st_flower&days_10st_flower | days_1st_flower&days_10st_flower | upstream | . | . | PF00010:Helix-loop-helix DNA-binding domain |
| Chr2_4107745 | 2 | Pm004330 | days_1st_flower | days_1st_flower | intergenic | . | . | PF00010:Helix-loop-helix DNA-binding domain |
| Chr2_4114812 | 2 | Pm004331 | days_10st_flower | days_10st_flower | intergenic | . | . | - |
| Chr2_4145859 | 2 | Pm004335 | days_1st_flower&days_5pct&days_25pct&days_75pct | days_1st_flower&days_5pct&days_25pct&days_75pct | intergenic | . | . | PF08263:Leucine rich repeat N-terminal domain\|PF13855:Leucine rich repeat |
| Chr2_41555047 | 2 | Pm009534 | days_1st_flower&days_5pct&days_25pct | days_1st_flower&days_5pct&days_25pct | intergenic | . | . | PF00012:Hsp70 protein |
| Chr2_4197834 | 2 | Pm004346 | days_1st_flower&days_10st_flower | days_1st_flower&days_10st_flower | downstream | . | . | PF12854:PPR repeat\|PF13041:PPR repeat family |
| Chr2_4199958 | 2 | Pm004346 | days_1st_flower | days_1st_flower | exonic | nonsynonymous | Pm004346:Pm004346.1:exon1:c.T94G:p.W32G | PF12854:PPR repeat\|PF13041:PPR repeat family |
| Chr2_4212605 | 2 | Pm004350 | days_1st_flower | days_1st_flower | exonic | synonymous | Pm004350:Pm004350.1:exon4:c.C390T:p.L130L | PF01486:K-box region |
| Chr2_4214019 | 2 | Pm004349 | days_1st_flower | days_1st_flower | intronic | . | . | PF00319:SRF-type transcription factor (DNA-binding and dimerisation domain)\|PF01486:K-box region |
| Chr2_4228912 | 2 | Pm004349 | days_10st_flower | days_10st_flower | intronic | . | . | PF00319:SRF-type transcription factor (DNA-binding and dimerisation domain)\|PF01486:K-box region |
| Chr2_4264898 | 2 | Pm004353 | days_1st_flower&days_10st_flower | days_1st_flower&days_10st_flower | intronic | . | . | PF00067:Cytochrome P450 |
| Chr2_4264900 | 2 | Pm004353 | days_1st_flower&days_10st_flower | days_1st_flower&days_10st_flower | intronic | . | . | PF00067:Cytochrome P450 |
| Chr2_4286263 | 2 | Pm004356 | days_1st_flower&days_10st_flower | days_1st_flower&days_10st_flower | exonic | synonymous | Pm004356:Pm004356.1:exon3:c.G927A:p.E309E | PF00067:Cytochrome P450 |
| Chr2_4318646 | 2 | Pm004358 | days_10st_flower | days_10st_flower | upstream | . | . | PF00067:Cytochrome P450 |
| Chr2_4322961 | 2 | Pm004359 | days_1st_flower | days_1st_flower | exonic | nonsynonymous | Pm004359:Pm004359.1:exon1:c.C47T:p.A16V | PF14683:Polysaccharide lyase family 4, domain III\|PF06045:Rhamnogalacturonate lyase family\|PF14686:Polysaccharide lyase family 4, domain II |
| Chr2_4419385 | 2 | Pm004368 | days_1st_flower&days_5pct | days_1st_flower&days_5pct | upstream | . | . | PF02751:Transcription initiation factor IIA, gamma subunit\|PF02268:Transcription initiation factor IIA, gamma subunit, helical domain |
| Chr2_4439858 | 2 | Pm004370 | days_1st_flower | days_1st_flower | exonic | nonsynonymous | Pm004370:Pm004370.1:exon1:c.G187T:p.A63S | PF01582:TIR domain |
| Chr2_4495379 | 2 | Pm004378 | days_1st_flower&days_10st_flower | days_1st_flower&days_10st_flower | downstream | . | . | PF04000:Sas10/Utp3/C1D family |
| Chr2_4513789 | 2 | Pm004381 | days_1st_flower | days_1st_flower | intergenic | . | . | PF13456:Reverse transcriptase-like\|PF03134:TB2/DP1, HVA22 family |
| Chr2_4682517 | 2 | Pm004403 | days_1st_flower | days_1st_flower | upstream | . | . | PF02458:Transferase family |
| Chr2_4811832 | 2 | Pm004415 | days_1st_flower | days_1st_flower | exonic | nonsynonymous | Pm004415:Pm004415.1:exon7:c.A608G:p.D203G | PF01486:K-box region\|PF00319:SRF-type transcription factor (DNA-binding and dimerisation domain) |
| Chr2_4833781 | 2 | Pm004417 | days_1st_flower | days_1st_flower | intronic | . | . | PF01486:K-box region\|PF00319:SRF-type transcription factor (DNA-binding and dimerisation domain) |
| Chr2_4847015 | 2 | Pm004418 | days_1st_flower | days_1st_flower | intronic | . | . | PF01486:K-box region\|PF00319:SRF-type transcription factor (DNA-binding and dimerisation domain) |
| Chr2_4852782 | 2 | Pm004419 | days_10st_flower | days_10st_flower | intergenic | . | . | PF00319:SRF-type transcription factor (DNA-binding and dimerisation domain)\|PF01486:K-box region |
| Chr2_4868493 | 2 | Pm004420 | days_1st_flower | days_1st_flower | intronic | . | . | PF01486:K-box region\|PF00319:SRF-type transcription factor (DNA-binding and dimerisation domain) |
| Chr2_4944971 | 2 | Pm004428 | days_1st_flower | days_1st_flower | intergenic | . | . | PF08241:Methyltransferase domain |
| Chr2_4966012 | 2 | Pm004430 | days_1st_flower | days_1st_flower | upstream | . | . | PF03479:Domain of unknown function (DUF296) |
| Chr2_4966409 | 2 | Pm004430 | days_1st_flower | days_1st_flower | exonic | synonymous | Pm004430:Pm004430.1:exon1:c.T132C:p.F44F | PF03479:Domain of unknown function (DUF296) |
| Chr2_4975251 | 2 | Pm004431 | days_1st_flower | days_1st_flower | intergenic | . | . | PF00443:Ubiquitin carboxyl-terminal hydrolase\|PF01753:MYND finger |
| Chr2_5094129 | 2 | Pm004451 | days_1st_flower&days_10st_flower&days_5pct&days_25pct&days_75pct | days_1st_flower&days_10st_flower&days_5pct&days_25pct&days_75pct | intronic | . | . | PF00201:UDP-glucoronosyl and UDP-glucosyl transferase |
| Chr2_5094132 | 2 | Pm004451 | days_1st_flower&days_10st_flower&days_5pct&days_25pct&days_75pct | days_1st_flower&days_10st_flower&days_5pct&days_25pct&days_75pct | intronic | . | . | PF00201:UDP-glucoronosyl and UDP-glucosyl transferase |
| Chr2_5209676 | 2 | Pm004468 | days_1st_flower | days_1st_flower | upstream | . | . | PF05678:VQ motif |
| Chr2_5235999 | 2 | Pm004473 | days_1st_flower | days_1st_flower | intronic | . | . | PF10250:GDP-fucose protein O-fucosyltransferase |
| Chr2_5293320 | 2 | Pm004480 | days_1st_flower&days_10st_flower&days_5pct | days_1st_flower&days_10st_flower&days_5pct | intergenic | . | . | PF02922:Carbohydrate-binding module 48 (Isoamylase N-terminal domain)\|PF00128:Alpha amylase, catalytic domain |
| Chr2_5293729 | 2 | Pm004480 | days_1st_flower | days_1st_flower | upstream | . | . | PF02922:Carbohydrate-binding module 48 (Isoamylase N-terminal domain)\|PF00128:Alpha amylase, catalytic domain |
| Chr2_5293772 | 2 | Pm004480 | days_1st_flower | days_1st_flower | upstream | . | . | PF02922:Carbohydrate-binding module 48 (Isoamylase N-terminal domain)\|PF00128:Alpha amylase, catalytic domain |
| Chr2_5315365 | 2 | Pm004481 | days_1st_flower&days_10st_flower&days_5pct | days_1st_flower&days_10st_flower&days_5pct | intergenic | . | . | PF02298:Plastocyanin-like domain |
| Chr2_5386295 | 2 | Pm004491 | days_1st_flower | days_1st_flower | upstream | . | . | PF07651:ANTH domain |
| Chr2_5402772 | 2 | Pm004497 | days_75pct | days_75pct | intronic | . | . | - |
| Chr2_5445489 | 2 | Pm004502 | days_1st_flower | days_1st_flower | downstream | . | . | PF00232:Glycosyl hydrolase family 1 |
| Chr2_5523888 | 2 | Pm004519 | days_1st_flower | days_1st_flower | upstream | . | . | PF01243:Pyridoxamine 5'-phosphate oxidase\|PF10590:Pyridoxine 5'-phosphate oxidase C-terminal dimerisation region\|PF03853:YjeF-related protein N-terminus |
| Chr2_5625389 | 2 | Pm004529 | days_1st_flower | days_1st_flower | intergenic | . | . | PF02984:Cyclin, C-terminal domain\|PF00134:Cyclin, N-terminal domain |
| Chr2_5630248 | 2 | Pm004530 | days_1st_flower&days_5pct&days_25pct&days_75pct | days_1st_flower&days_5pct&days_25pct&days_75pct | intergenic | . | . | PF03091:CutA1 divalent ion tolerance protein |
| Chr2_5637500 | 2 | Pm004530 | days_5pct&days_25pct&days_75pct | days_5pct&days_25pct&days_75pct | downstream | . | . | PF03091:CutA1 divalent ion tolerance protein |
| Chr2_5645127 | 2 | Pm004533 | days_1st_flower&days_10st_flower&days_5pct&days_25pct&days_75pct | days_1st_flower&days_10st_flower&days_5pct&days_25pct&days_75pct | downstream | . | . | PF00226:DnaJ domain |
| Chr2_5648793 | 2 | Pm004533 | days_5pct | days_5pct | intronic | . | . | PF00226:DnaJ domain |
| Chr2_5648974 | 2 | Pm004533 | days_1st_flower&days_10st_flower&days_5pct&days_25pct&days_75pct | days_1st_flower&days_10st_flower&days_5pct&days_25pct&days_75pct | exonic | synonymous | Pm004533:Pm004533.1:exon1:c.G60C:p.P20P | PF00226:DnaJ domain |
| Chr2_5653188 | 2 | Pm004534 | days_10st_flower&days_5pct&days_25pct&days_75pct | days_10st_flower&days_5pct&days_25pct&days_75pct | intronic | . | . | PF00342:Phosphoglucose isomerase |
| Chr2_5656059 | 2 | Pm004534 | days_1st_flower&days_10st_flower&days_5pct&days_25pct | days_1st_flower&days_10st_flower&days_5pct&days_25pct | intronic | . | . | PF00342:Phosphoglucose isomerase |
| Chr2_5656449 | 2 | Pm004534 | days_1st_flower&days_5pct&days_25pct&days_75pct | days_1st_flower&days_5pct&days_25pct&days_75pct | exonic | synonymous | Pm004534:Pm004534.1:exon8:c.G1176A:p.T392T | PF00342:Phosphoglucose isomerase |
| Chr2_5657271 | 2 | Pm004534 | days_5pct&days_25pct | days_5pct&days_25pct | exonic | synonymous | Pm004534:Pm004534.1:exon12:c.C1602T:p.I534I | PF00342:Phosphoglucose isomerase |
| Chr2_5657274 | 2 | Pm004534 | days_5pct&days_25pct | days_5pct&days_25pct | exonic | nonsynonymous | Pm004534:Pm004534.1:exon12:c.C1605G:p.H535Q | PF00342:Phosphoglucose isomerase |
| Chr2_5659125 | 2 | Pm004534 | days_5pct&days_25pct | days_5pct&days_25pct | intergenic | . | . | PF00342:Phosphoglucose isomerase |
| Chr2_5664582 | 2 | Pm004535 | days_10st_flower&days_5pct&days_25pct | days_10st_flower&days_5pct&days_25pct | intronic | . | . | - |
| Chr2_5665185 | 2 | Pm004535 | days_1st_flower&days_10st_flower&days_5pct&days_25pct&days_75pct | days_1st_flower&days_10st_flower&days_5pct&days_25pct&days_75pct | intronic | . | . | - |
| Chr2_5665740 | 2 | Pm004535 | days_1st_flower&days_10st_flower&days_5pct&days_25pct&days_75pct | days_1st_flower&days_10st_flower&days_5pct&days_25pct&days_75pct | intronic | . | . | - |
| Chr2_5666809 | 2 | Pm004535 | days_1st_flower&days_10st_flower&days_5pct&days_25pct | days_1st_flower&days_10st_flower&days_5pct&days_25pct | intronic | . | . | - |
| Chr2_5666843 | 2 | Pm004535 | days_1st_flower&days_10st_flower&days_5pct&days_25pct&days_75pct | days_1st_flower&days_10st_flower&days_5pct&days_25pct&days_75pct | intronic | . | . | - |
| Chr2_5666891 | 2 | Pm004535 | days_1st_flower&days_10st_flower&days_5pct&days_25pct&days_75pct | days_1st_flower&days_10st_flower&days_5pct&days_25pct&days_75pct | intronic | . | . | - |
| Chr2_5671387 | 2 | Pm004536 | days_1st_flower&days_10st_flower&days_5pct&days_25pct&days_75pct | days_1st_flower&days_10st_flower&days_5pct&days_25pct&days_75pct | intronic | . | . | PF00586:AIR synthase related protein, N-terminal domain\|PF02769:AIR synthase related protein, C-terminal domain |
| Chr2_5671740 | 2 | Pm004536 | days_1st_flower&days_10st_flower&days_5pct&days_25pct&days_75pct | days_1st_flower&days_10st_flower&days_5pct&days_25pct&days_75pct | intronic | . | . | PF00586:AIR synthase related protein, N-terminal domain\|PF02769:AIR synthase related protein, C-terminal domain |
| Chr2_5671753 | 2 | Pm004536 | days_1st_flower&days_10st_flower&days_5pct&days_25pct&days_75pct | days_1st_flower&days_10st_flower&days_5pct&days_25pct&days_75pct | intronic | . | . | PF00586:AIR synthase related protein, N-terminal domain\|PF02769:AIR synthase related protein, C-terminal domain |
| Chr2_5673572 | 2 | Pm004537 | days_1st_flower&days_10st_flower&days_5pct&days_25pct&days_75pct | days_1st_flower&days_10st_flower&days_5pct&days_25pct&days_75pct | upstream;downstream | . | . | - |
| Chr2_5711604 | 2 | Pm004544 | days_1st_flower&days_10st_flower&days_5pct | days_1st_flower&days_10st_flower&days_5pct | downstream | . | . | - |
| Chr2_5721031 | 2 | Pm004545 | days_10st_flower | days_10st_flower | intergenic | . | . | PF03372:Endonuclease/Exonuclease/phosphatase family\|PF15801:zf-MYND-like zinc finger, mRNA-binding |
| Chr2_5760648 | 2 | Pm004554 | days_1st_flower&days_10st_flower | days_1st_flower&days_10st_flower | upstream | . | . | PF03966:Trm112p-like protein |
| Chr2_5767970 | 2 | Pm004556 | days_1st_flower&days_10st_flower&days_5pct | days_1st_flower&days_10st_flower&days_5pct | exonic | nonsynonymous | Pm004556:Pm004556.1:exon1:c.G76C:p.E26Q | PF06645:Microsomal signal peptidase 12 kDa subunit (SPC12) |
| Chr2_5799222 | 2 | Pm004561 | days_1st_flower | days_1st_flower | upstream | . | . | PF00481:Protein phosphatase 2C |
| Chr2_5816474 | 2 | Pm004565 | days_1st_flower | days_1st_flower | intronic | . | . | PF00244:14-3-3 protein |
| Chr2_5843546 | 2 | Pm004569 | days_1st_flower | days_1st_flower | downstream | . | . | PF13855:Leucine rich repeat |
| Chr2_5888880 | 2 | Pm004575 | days_1st_flower&days_10st_flower | days_1st_flower&days_10st_flower | upstream | . | . | PF00201:UDP-glucoronosyl and UDP-glucosyl transferase |
| Chr2_6014139 | 2 | Pm004603 | days_1st_flower&days_10st_flower | days_1st_flower&days_10st_flower | upstream | . | . | PF03151:Triose-phosphate Transporter family |
| Chr2_6014158 | 2 | Pm004603 | days_1st_flower&days_10st_flower | days_1st_flower&days_10st_flower | upstream | . | . | PF03151:Triose-phosphate Transporter family |
| Chr2_6070714 | 2 | Pm004614 | days_1st_flower&days_25pct&days_75pct | days_1st_flower&days_25pct&days_75pct | intronic | . | . | PF03105:SPX domain |
| Chr2_6122565 | 2 | Pm004620 | days_1st_flower | days_1st_flower | intronic | . | . | PF00141:Peroxidase |
| Chr2_6122575 | 2 | Pm004620 | days_1st_flower | days_1st_flower | intronic | . | . | PF00141:Peroxidase |
| Chr2_6123371 | 2 | Pm004620 | days_1st_flower&days_10st_flower&days_5pct | days_1st_flower&days_10st_flower&days_5pct | intronic | . | . | PF00141:Peroxidase |
| Chr2_6204072 | 2 | Pm004633 | days_1st_flower | days_1st_flower | intergenic | . | . | - |
| Chr2_6221640 | 2 | Pm004635 | days_1st_flower&days_10st_flower&days_5pct | days_1st_flower&days_10st_flower&days_5pct | intergenic | . | . | PF00787:PX domain |
| Chr2_6320708 | 2 | Pm004655 | days_1st_flower | days_1st_flower | intergenic | . | . | - |
| Chr2_6329249 | 2 | Pm004656 | days_1st_flower&days_10st_flower&days_5pct | days_1st_flower&days_10st_flower&days_5pct | intergenic | . | . | - |
| Chr2_6334768 | 2 | Pm004658 | days_10st_flower | days_10st_flower | upstream | . | . | - |
| Chr2_6555282 | 2 | Pm004687 | days_1st_flower | days_1st_flower | downstream | . | . | - |
| Chr2_6555722 | 2 | Pm004687 | days_1st_flower | days_1st_flower | downstream | . | . | - |
| Chr2_6672854 | 2 | Pm004708 | days_1st_flower | days_1st_flower | downstream | . | . | PF13499:EF-hand domain pair\|PF00036:EF hand |
| Chr2_6888254 | 2 | Pm004737 | days_10st_flower | days_10st_flower | intergenic | . | . | PF00717:Peptidase S24-like |
| Chr2_6987718 | 2 | Pm004756 | days_1st_flower | days_1st_flower | upstream | . | . | PF00227:Proteasome subunit |
| Chr2_6987719 | 2 | Pm004756 | days_1st_flower | days_1st_flower | upstream | . | . | PF00227:Proteasome subunit |
| Chr2_6987721 | 2 | Pm004756 | days_1st_flower | days_1st_flower | upstream | . | . | PF00227:Proteasome subunit |
| Chr2_7044896 | 2 | Pm004764 | days_1st_flower | days_1st_flower | exonic | synonymous | Pm004764:Pm004764.1:exon2:c.T462C:p.P154P | PF01556:DnaJ C terminal domain |
| Chr2_7056837 | 2 | Pm004765 | days_1st_flower | days_1st_flower | upstream | . | . | PF00171:Aldehyde dehydrogenase family |
| Chr2_7110567 | 2 | Pm004774 | days_1st_flower | days_1st_flower | intergenic | . | . | PF00891:O-methyltransferase\|PF08100:Dimerisation domain\|PF12937:F-box-like |
| Chr2_7202599 | 2 | Pm004795 | days_25pct | days_25pct | intergenic | . | . | PF00657:GDSL-like Lipase/Acylhydrolase |
| Chr2_7244089 | 2 | Pm004801 | days_1st_flower&days_10st_flower | days_1st_flower&days_10st_flower | intergenic | . | . | - |
| Chr2_7284103 | 2 | Pm004807 | days_1st_flower&days_10st_flower&days_5pct | days_1st_flower&days_10st_flower&days_5pct | intergenic | . | . | PF14226:non-haem dioxygenase in morphine synthesis N-terminal\|PF03171:2OG-Fe(II) oxygenase superfamily |
| Chr2_7284112 | 2 | Pm004807 | days_1st_flower | days_1st_flower | intergenic | . | . | PF14226:non-haem dioxygenase in morphine synthesis N-terminal\|PF03171:2OG-Fe(II) oxygenase superfamily |
| Chr2_7313277 | 2 | Pm004813 | days_1st_flower&days_10st_flower | days_1st_flower&days_10st_flower | intergenic | . | . | PF03151:Triose-phosphate Transporter family |
| Chr2_7434529 | 2 | Pm004827 | days_1st_flower&days_10st_flower&days_5pct&days_75pct | days_1st_flower&days_10st_flower&days_5pct&days_75pct | exonic | synonymous | Pm004827:Pm004827.1:exon1:c.T39C:p.P13P | PF03168:Late embryogenesis abundant protein |
| Chr2_7450871 | 2 | Pm004832 | days_5pct | days_5pct | upstream | . | . | - |
| Chr2_7465918 | 2 | Pm004834 | days_1st_flower | days_1st_flower | downstream | . | . | - |
| Chr2_7481490 | 2 | Pm004836 | days_5pct | days_5pct | upstream | . | . | PF07690:Major Facilitator Superfamily |
| Chr2_7595218 | 2 | Pm004852 | days_1st_flower | days_1st_flower | downstream | . | . | PF13855:Leucine rich repeat\|PF00069:Protein kinase domain |
| Chr2_7643744 | 2 | Pm004858 | days_5pct&days_25pct&days_75pct | days_5pct&days_25pct&days_75pct | intergenic | . | . | PF00177:Ribosomal protein S7p/S5e |
| Chr2_7706418 | 2 | Pm004868 | days_75pct | days_75pct | intergenic | . | . | PF14369:zinc-ribbon\|PF13639:Ring finger domain |
| Chr2_7706420 | 2 | Pm004868 | days_75pct | days_75pct | intergenic | . | . | PF14369:zinc-ribbon\|PF13639:Ring finger domain |
| Chr2_7923396 | 2 | Pm004896 | days_1st_flower | days_1st_flower | downstream | . | . | PF08276:PAN-like domain\|PF01453:D-mannose binding lectin |
| Chr2_8105946 | 2 | Pm004921 | days_10st_flower | days_10st_flower | upstream;downstream | . | . | PF13041:PPR repeat family\|PF12854:PPR repeat |
| Chr2_8146220 | 2 | Pm004929 | days_1st_flower | days_1st_flower | intronic | . | . | PF05185:PRMT5 arginine-N-methyltransferase |
| Chr2_820087 | 2 | Pm003867 | days_10st_flower | days_10st_flower | upstream | . | . | - |
| Chr2_8225325 | 2 | Pm004940 | days_1st_flower&days_10st_flower | days_1st_flower&days_10st_flower | exonic | synonymous | Pm004940:Pm004940.1:exon14:c.A3570C:p.V1190V | - |
| Chr2_8248298 | 2 | Pm004942 | days_1st_flower&days_10st_flower&days_5pct&days_25pct | days_1st_flower&days_10st_flower&days_5pct&days_25pct | intergenic | . | . | - |
| Chr2_8273806 | 2 | Pm004947 | days_1st_flower | days_1st_flower | intronic | . | . | - |
| Chr2_8312178 | 2 | Pm004953 | days_1st_flower | days_1st_flower | intronic | . | . | - |
| Chr2_8405828 | 2 | Pm004969 | days_1st_flower&days_10st_flower | days_1st_flower&days_10st_flower | intergenic | . | . | - |
| Chr2_8449735 | 2 | Pm004972 | days_5pct | days_5pct | intergenic | . | . | PF00743:Flavin-binding monooxygenase-like |
| Chr2_8847380 | 2 | Pm005035 | days_1st_flower&days_10st_flower | days_1st_flower&days_10st_flower | upstream | . | . | PF00141:Peroxidase |
| Chr2_8890283 | 2 | Pm005044 | days_1st_flower | days_1st_flower | exonic | synonymous | Pm005044:Pm005044.1:exon1:c.G1142A:p.X381X | - |
| Chr2_8918924 | 2 | Pm005052 | days_1st_flower&days_10st_flower&days_5pct | days_1st_flower&days_10st_flower&days_5pct | intergenic | . | . | PF03106:WRKY DNA -binding domain\|PF10533:Plant zinc cluster domain |
| Chr2_8966920 | 2 | Pm005060 | days_1st_flower&days_10st_flower&days_5pct | days_1st_flower&days_10st_flower&days_5pct | exonic | nonsynonymous | Pm005060:Pm005060.1:exon1:c.T329G:p.I110S | PF00201:UDP-glucoronosyl and UDP-glucosyl transferase |
| Chr2_8998390 | 2 | Pm005065 | days_1st_flower | days_1st_flower | upstream | . | . | PF04818:RNA polymerase II-binding domain. |
| Chr2_9054327 | 2 | Pm005075 | days_1st_flower&days_10st_flower&days_5pct&days_75pct | days_1st_flower&days_10st_flower&days_5pct&days_75pct | intronic | . | . | PF13837:Myb/SANT-like DNA-binding domain |
| Chr2_9063718 | 2 | Pm005076 | days_1st_flower | days_1st_flower | intronic | . | . | PF04752:ChaC-like protein |
| Chr2_9181063 | 2 | Pm005101 | days_1st_flower&days_10st_flower | days_1st_flower&days_10st_flower | intronic | . | . | PF00271:Helicase conserved C-terminal domain\|PF00270:DEAD/DEAH box helicase\|PF00476:DNA polymerase family A |
| Chr2_9238527 | 2 | Pm005108 | days_1st_flower&days_10st_flower | days_1st_flower&days_10st_flower | intergenic | . | . | - |
| Chr2_9241880 | 2 | Pm005109 | days_1st_flower | days_1st_flower | intronic | . | . | - |
| Chr2_9290369 | 2 | Pm005116 | days_1st_flower | days_1st_flower | exonic | nonsynonymous | Pm005116:Pm005116.1:exon1:c.G256C:p.V86L | PF00069:Protein kinase domain |
| Chr2_9305028 | 2 | Pm005120 | days_10st_flower | days_10st_flower | intronic | . | . | PF04258:Signal peptide peptidase |
| Chr2_9373083 | 2 | Pm005132 | days_10st_flower | days_10st_flower | exonic | synonymous | Pm005132:Pm005132.1:exon10:c.T990C:p.N330N | PF12695:Alpha/beta hydrolase family |
| Chr2_9373174 | 2 | Pm005132 | days_10st_flower | days_10st_flower | intronic | . | . | PF12695:Alpha/beta hydrolase family |
| Chr2_9376122 | 2 | Pm005132 | days_10st_flower | days_10st_flower | upstream | . | . | PF12695:Alpha/beta hydrolase family |
| Chr2_9436741 | 2 | Pm005144 | days_1st_flower&days_10st_flower&days_5pct | days_1st_flower&days_10st_flower&days_5pct | intronic | . | . | PF00069:Protein kinase domain\|PF05703:Auxin canalisation\|PF08458:Plant pleckstrin homology-like region |
| Chr2_9475311 | 2 | Pm005150 | days_10st_flower | days_10st_flower | intronic | . | . | PF03016:Exostosin family |
| Chr2_9535646 | 2 | Pm005157 | days_1st_flower | days_1st_flower | intronic | . | . | PF07575:Nup85 Nucleoporin |
| Chr2_9566020 | 2 | Pm005162 | days_1st_flower | days_1st_flower | intergenic | . | . | - |
| Chr2_9692722 | 2 | Pm005183 | days_1st_flower | days_1st_flower | intergenic | . | . | PF01490:Transmembrane amino acid transporter protein |
| Chr2_9734596 | 2 | Pm005192 | days_75pct | days_75pct | intronic | . | . | - |
| Chr2_9745683 | 2 | Pm005197 | days_25pct&days_75pct | days_25pct&days_75pct | downstream | . | . | PF00235:Profilin |
| Chr2_9831470 | 2 | Pm005212 | days_10st_flower | days_10st_flower | upstream | . | . | PF07645:Calcium-binding EGF domain\|PF13947:Wall-associated receptor kinase galacturonan-binding\|PF00069:Protein kinase domain |
| Chr2_9855809 | 2 | Pm005216 | days_75pct | days_75pct | downstream | . | . | - |
| Chr2_9914977 | 2 | Pm005227 | days_10st_flower&days_5pct | days_10st_flower&days_5pct | intergenic | . | . | PF00450:Serine carboxypeptidase |
| Chr2_9983230 | 2 | Pm005239 | days_1st_flower | days_1st_flower | intronic | . | . | PF01734:Patatin-like phospholipase |
| Chr3_10583967 | 3 | Pm011363 | days_10st_flower | days_10st_flower | intergenic | . | . | - |
| Chr3_11705402 | 3 | Pm011522 | days_5pct | days_5pct | downstream | . | . | PF01554:MatE |
| Chr3_11736714 | 3 | Pm011527 | days_5pct | days_5pct | intronic | . | . | PF01554:MatE |
| Chr3_11736772 | 3 | Pm011527 | days_5pct&days_75pct | days_5pct&days_75pct | intronic | . | . | PF01554:MatE |
| Chr3_11869444 | 3 | Pm011544 | days_10st_flower | days_10st_flower | intergenic | . | . | PF00139:Legume lectin domain\|PF00069:Protein kinase domain |
| Chr3_11968120 | 3 | Pm011565 | days_5pct&days_25pct&days_75pct | days_5pct&days_25pct&days_75pct | intergenic | . | . | PF13923:Zinc finger, C3HC4 type (RING finger) |
| Chr3_14480798 | 3 | Pm011904 | days_75pct | days_75pct | intronic | . | . | PF11976:Ubiquitin-2 like Rad60 SUMO-like |
| Chr3_17467131 | 3 | Pm012211 | days_10st_flower | days_10st_flower | intergenic | . | . | - |
| Chr3_17471249 | 3 | Pm012211 | days_10st_flower | days_10st_flower | intergenic | . | . | - |
| Chr3_17488914 | 3 | Pm012213 | days_1st_flower&days_10st_flower&days_25pct&days_75pct | days_1st_flower&days_10st_flower&days_25pct&days_75pct | intergenic | . | . | - |
| Chr3_18794925 | 3 | Pm012333 | days_5pct&days_25pct&days_75pct | days_5pct&days_25pct&days_75pct | intergenic | . | . | - |
| Chr3_2448505 | 3 | Pm010084 | days_1st_flower | days_1st_flower | intergenic | . | . | PF03226:Yippee zinc-binding/DNA-binding /Mis18, centromere assembly |
| Chr3_2646965 | 3 | Pm010116 | days_5pct | days_5pct | downstream | . | . | PF00271:Helicase conserved C-terminal domain\|PF07717:Oligonucleotide/oligosaccharide-binding (OB)-fold\|PF04408:Helicase associated domain (HA2) |
| Chr3_4106100 | 3 | Pm010369 | days_1st_flower&days_5pct | days_1st_flower&days_5pct | exonic | nonsynonymous | Pm010369:Pm010369.1:exon1:c.A183T:p.K61N | PF03195:Protein of unknown function DUF260 |
| Chr3_4106166 | 3 | Pm010369 | days_1st_flower&days_5pct | days_1st_flower&days_5pct | exonic | nonsynonymous | Pm010369:Pm010369.1:exon1:c.G117C:p.K39N | PF03195:Protein of unknown function DUF260 |
| Chr3_697285 | 3 | Pm009736 | days_75pct | days_75pct | exonic | nonsynonymous | Pm009736:Pm009736.1:exon2:c.T850C:p.S284P | PF01657:Salt stress response/antifungal\|PF07714:Protein tyrosine kinase |
| Chr3_8246194 | 3 | Pm010996 | days_1st_flower&days_5pct&days_25pct&days_75pct | days_1st_flower&days_5pct&days_25pct&days_75pct | intergenic | . | . | PF02458:Transferase family |
| Chr3_9103601 | 3 | Pm011117 | days_10st_flower | days_10st_flower | exonic | synonymous | Pm011117:Pm011117.1:exon7:c.T918A:p.A306A | PF04194:Programmed cell death protein 2, C-terminal putative domain |
| Chr4_11495754 | 4 | Pm014307 | days_25pct | days_25pct | intergenic | . | . | PF13637:Ankyrin repeats (many copies)\|PF13962:Domain of unknown function\|PF12796:Ankyrin repeats (3 copies) |
| Chr4_14059996 | 4 | Pm014592 | days_25pct | days_25pct | upstream | . | . | - |
| Chr4_1731651 | 4 | Pm013104 | days_10st_flower | days_10st_flower | intergenic | . | . | PF13855:Leucine rich repeat |
| Chr4_19096773 | 4 | Pm015379 | days_25pct | days_25pct | intergenic | . | . | PF08100:Dimerisation domain\|PF00891:O-methyltransferase |
| Chr4_22889717 | 4 | Pm016057 | days_10st_flower | days_10st_flower | exonic | nonsynonymous | Pm016057:Pm016057.1:exon1:c.G835T:p.D279Y | PF12819:Carbohydrate-binding protein of the ER |
| Chr4_6386983 | 4 | Pm013749 | days_1st_flower&days_75pct | days_1st_flower&days_75pct | downstream | . | . | PF14709:double strand RNA binding domain from DEAD END PROTEIN 1 |
| Chr5_11491322 | 5 | Pm017471 | days_25pct | days_25pct | upstream | . | . | PF00854:POT family |
| Chr5_13352054 | 5 | Pm017697 | days_1st_flower | days_1st_flower | intergenic | . | . | PF00847:AP2 domain |
| Chr5_13565187 | 5 | Pm017719 | days_10st_flower | days_10st_flower | exonic | synonymous | Pm017719:Pm017719.1:exon1:c.G2352T:p.L784L | PF13855:Leucine rich repeat\|PF08263:Leucine rich repeat N-terminal domain |
| Chr5_13693049 | 5 | Pm017737 | days_10st_flower | days_10st_flower | intergenic | . | . | - |
| Chr5_13693164 | 5 | Pm017737 | days_10st_flower | days_10st_flower | intergenic | . | . | - |
| Chr5_14191195 | 5 | Pm017800 | days_25pct | days_25pct | intergenic | . | . | PF00010:Helix-loop-helix DNA-binding domain |
| Chr5_14968510 | 5 | Pm017912 | days_25pct | days_25pct | upstream | . | . | PF00646:F-box domain |
| Chr5_1529942 | 5 | Pm016446 | days_75pct | days_75pct | intergenic | . | . | - |
| Chr5_171178 | 5 | Pm016279 | days_75pct | days_75pct | upstream | . | . | PF14009:Domain of unknown function (DUF4228) |
| Chr5_18877920 | 5 | Pm018511 | days_25pct | days_25pct | downstream | . | . | - |
| Chr5_21999234 | 5 | Pm019078 | days_1st_flower&days_10st_flower&days_5pct | days_1st_flower&days_10st_flower&days_5pct | upstream;downstream | . | . | PF01453:D-mannose binding lectin\|PF00954:S-locus glycoprotein domain\|PF00069:Protein kinase domain |
| Chr5_21999579 | 5 | Pm019078 | days_1st_flower | days_1st_flower | exonic | nonsynonymous | Pm019078:Pm019078.1:exon1:c.A286G:p.S96G | PF01453:D-mannose binding lectin\|PF00954:S-locus glycoprotein domain\|PF00069:Protein kinase domain |
| Chr5_22037290 | 5 | Pm019085 | days_1st_flower&days_10st_flower | days_1st_flower&days_10st_flower | intronic | . | . | PF02537:CrcB-like protein, Camphor Resistance (CrcB) |
| Chr5_22103351 | 5 | Pm019096 | days_1st_flower | days_1st_flower | intergenic | . | . | PF02237:Biotin protein ligase C terminal domain\|PF03099:Biotin/lipoate A/B protein ligase family |
| Chr5_22158046 | 5 | Pm019103 | days_10st_flower | days_10st_flower | intergenic | . | . | PF04059:RNA recognition motif 2 |
| Chr5_22205472 | 5 | Pm019113 | days_1st_flower&days_10st_flower | days_1st_flower&days_10st_flower | intronic | . | . | PF00069:Protein kinase domain |
| Chr5_22205489 | 5 | Pm019113 | days_1st_flower&days_10st_flower | days_1st_flower&days_10st_flower | intronic | . | . | PF00069:Protein kinase domain |
| Chr5_22227959 | 5 | Pm019116 | days_1st_flower&days_10st_flower&days_5pct | days_1st_flower&days_10st_flower&days_5pct | intergenic | . | . | - |
| Chr5_22284126 | 5 | Pm019125 | days_1st_flower&days_10st_flower | days_1st_flower&days_10st_flower | upstream | . | . | PF02020:eIF4-gamma/eIF5/eIF2-epsilon\|PF01873:Domain found in IF2B/IF5 |
| Chr5_22386076 | 5 | Pm019146 | days_1st_flower | days_1st_flower | exonic | nonsynonymous | Pm019146:Pm019146.1:exon1:c.G419T:p.G140V | PF01553:Acyltransferase |
| Chr5_22392285 | 5 | Pm019147 | days_10st_flower | days_10st_flower | intronic | . | . | PF08241:Methyltransferase domain |
| Chr5_22402025 | 5 | Pm019148 | days_1st_flower | days_1st_flower | intergenic | . | . | PF00155:Aminotransferase class I and II |
| Chr5_22446426 | 5 | Pm019155 | days_1st_flower | days_1st_flower | downstream | . | . | - |
| Chr5_22467770 | 5 | Pm019159 | days_1st_flower&days_10st_flower | days_1st_flower&days_10st_flower | intergenic | . | . | PF01344:Kelch motif |
| Chr5_22583214 | 5 | Pm019180 | days_1st_flower | days_1st_flower | upstream | . | . | - |
| Chr5_22620301 | 5 | Pm019185 | days_1st_flower | days_1st_flower | intergenic | . | . | PF00916:Sulfate permease family\|PF01740:STAS domain |
| Chr5_22655509 | 5 | Pm019188 | days_1st_flower | days_1st_flower | upstream | . | . | PF14368:Probable lipid transfer |
| Chr5_22656714 | 5 | Pm019188 | days_1st_flower | days_1st_flower | intergenic | . | . | PF14368:Probable lipid transfer |
| Chr5_22669692 | 5 | Pm019190 | days_1st_flower&days_10st_flower | days_1st_flower&days_10st_flower | downstream | . | . | PF00150:Cellulase (glycosyl hydrolase family 5) |
| Chr5_3565552 | 5 | Pm016660 | days_25pct | days_25pct | intergenic | . | . | - |
| Chr5_4085489 | 5 | Pm016717 | days_5pct&days_25pct&days_75pct | days_5pct&days_25pct&days_75pct | intergenic | . | . | - |
| Chr5_527717 | 5 | Pm016319 | days_25pct | days_25pct | intergenic | . | . | - |
| Chr5_9936281 | 5 | Pm017298 | days_25pct | days_25pct | intergenic | . | . | PF01095:Pectinesterase\|PF04043:Plant invertase/pectin methylesterase inhibitor |
| Chr6_10998272 | 6 | Pm021741 | days_10st_flower&days_5pct&days_25pct&days_75pct | days_10st_flower&days_5pct&days_25pct&days_75pct | intronic | . | . | PF00520:Ion transport protein\|PF01213:Adenylate cyclase associated (CAP) N terminal\|PF08603:Adenylate cyclase associated (CAP) C terminal |
| Chr6_11654215 | 6 | Pm021832 | days_75pct | days_75pct | intronic | . | . | PF14008:Iron/zinc purple acid phosphatase-like protein C\|PF00149:Calcineurin-like phosphoesterase\|PF16656:Purple acid Phosphatase, N-terminal domain |
| Chr6_12137893 | 6 | Pm021894 | days_75pct | days_75pct | intergenic | . | . | - |
| Chr6_12138296 | 6 | Pm021894 | days_1st_flower&days_10st_flower&days_25pct&days_75pct | days_1st_flower&days_10st_flower&days_25pct&days_75pct | intergenic | . | . | - |
| Chr6_12427822 | 6 | Pm021926 | days_10st_flower | days_10st_flower | upstream | . | . | PF02902:Ulp1 protease family, C-terminal catalytic domain |
| Chr6_14931552 | 6 | Pm022187 | days_10st_flower&days_5pct&days_25pct&days_75pct | days_10st_flower&days_5pct&days_25pct&days_75pct | intergenic | . | . | PF09335:SNARE associated Golgi protein |
| Chr6_14932790 | 6 | Pm022187 | days_5pct&days_25pct&days_75pct | days_5pct&days_25pct&days_75pct | intergenic | . | . | PF09335:SNARE associated Golgi protein |
| Chr6_14933113 | 6 | Pm022187 | days_10st_flower&days_5pct&days_25pct&days_75pct | days_10st_flower&days_5pct&days_25pct&days_75pct | intergenic | . | . | PF09335:SNARE associated Golgi protein |
| Chr6_14934298 | 6 | Pm022188 | days_25pct&days_75pct | days_25pct&days_75pct | intergenic | . | . | - |
| Chr6_14934838 | 6 | Pm022188 | days_75pct | days_75pct | intergenic | . | . | - |
| Chr6_14934900 | 6 | Pm022187 | days_1st_flower&days_10st_flower&days_5pct&days_25pct&days_75pct | days_1st_flower&days_10st_flower&days_5pct&days_25pct&days_75pct | intergenic | . | . | PF09335:SNARE associated Golgi protein |
| Chr6_14934915 | 6 | Pm022187 | days_1st_flower&days_10st_flower&days_5pct&days_25pct&days_75pct | days_1st_flower&days_10st_flower&days_5pct&days_25pct&days_75pct | intergenic | . | . | PF09335:SNARE associated Golgi protein |
| Chr6_15844810 | 6 | Pm022288 | days_5pct&days_25pct&days_75pct | days_5pct&days_25pct&days_75pct | downstream | . | . | PF00931:NB-ARC domain\|PF01582:TIR domain |
| Chr6_17620817 | 6 | Pm022451 | days_10st_flower | days_10st_flower | intergenic | . | . | - |
| Chr6_18432922 | 6 | Pm022517 | days_10st_flower | days_10st_flower | intergenic | . | . | PF01918:Alba |
| Chr6_19492688 | 6 | Pm022634 | days_75pct | days_75pct | intronic | . | . | PF01582:TIR domain\|PF00931:NB-ARC domain |
| Chr6_20165105 | 6 | Pm022721 | days_75pct | days_75pct | upstream | . | . | PF00071:Ras family |
| Chr6_20576909 | 6 | Pm022778 | days_75pct | days_75pct | downstream | . | . | PF09768:Peptidase M76 family |
| Chr6_21248639 | 6 | Pm022861 | days_75pct | days_75pct | intergenic | . | . | PF01582:TIR domain\|PF00931:NB-ARC domain |
| Chr6_2279722 | 6 | Pm020300 | days_75pct | days_75pct | downstream | . | . | PF00067:Cytochrome P450 |
| Chr6_303685 | 6 | Pm019927 | days_25pct&days_75pct | days_25pct&days_75pct | intergenic | . | . | - |
| Chr6_7013795 | 6 | Pm021141 | days_75pct | days_75pct | intergenic | . | . | PF00412:LIM domain |
| Chr6_7616067 | 6 | Pm021253 | days_25pct | days_25pct | exonic | nonsynonymous | Pm021253:Pm021253.1:exon2:c.A197C:p.H66P | - |
| Chr7_10312630 | 7 | Pm024087 | days_1st_flower | days_1st_flower | exonic | nonsynonymous | Pm024087:Pm024087.1:exon2:c.G397A:p.D133N | - |
| Chr7_14803462 | 7 | Pm024875 | days_1st_flower | days_1st_flower | exonic | nonsynonymous | Pm024875:Pm024875.1:exon2:c.G859A:p.E287K | - |
| Chr7_15273604 | 7 | Pm024963 | days_1st_flower&days_10st_flower&days_5pct&days_25pct | days_1st_flower&days_10st_flower&days_5pct&days_25pct | intronic | . | . | PF00850:Histone deacetylase domain |
| Chr7_1946780 | 7 | Pm023079 | days_25pct | days_25pct | intergenic | . | . | PF03637:Mob1/phocein family |
| Chr7_3944702 | 7 | Pm023298 | days_75pct | days_75pct | upstream | . | . | - |
| Chr7_698351 | 7 | Pm022952 | days_75pct | days_75pct | intronic | . | . | PF00742:Homoserine dehydrogenase |
| Chr7_699427 | 7 | Pm022952 | days_75pct | days_75pct | intronic | . | . | PF00742:Homoserine dehydrogenase |
| Chr7_703206 | 7 | Pm022952 | days_75pct | days_75pct | intronic | . | . | PF00742:Homoserine dehydrogenase |
| Chr7_704223 | 7 | Pm022952 | days_75pct | days_75pct | exonic | synonymous | Pm022952:Pm022952.1:exon2:c.T153C:p.G51G | PF00742:Homoserine dehydrogenase |
| Chr7_7537926 | 7 | Pm023716 | days_75pct | days_75pct | upstream | . | . | - |
| Chr7_9016312 | 7 | Pm023880 | days_10st_flower | days_10st_flower | intronic | . | . | PF01429:Methyl-CpG binding domain |
| Chr8_10670923 | 8 | Pm026583 | days_75pct | days_75pct | exonic | nonsynonymous | Pm026583:Pm026583.1:exon2:c.G1228A:p.V410I | PF03140:Plant protein of unknown function |
| Chr8_14267798 | 8 | Pm027246 | days_75pct | days_75pct | intronic | . | . | - |
| Chr8_14416391 | 8 | Pm027276 | days_25pct&days_75pct | days_25pct&days_75pct | intergenic | . | . | PF01019:Gamma-glutamyltranspeptidase |
| Chr8_15019488 | 8 | Pm027391 | days_10st_flower | days_10st_flower | intergenic | . | . | - |
| Chr8_2669186 | 8 | Pm025547 | days_1st_flower | days_1st_flower | exonic | nonsynonymous | Pm025547:Pm025547.1:exon1:c.C221T:p.A74V | - |
| Chr8_3429824 | 8 |  | days_10st_flower | days_10st_flower | intergenic | . | . |  |
| Chr8_4992267 | 8 | Pm025770 | days_10st_flower&days_5pct&days_25pct | days_10st_flower&days_5pct&days_25pct | upstream | . | . | - |
| Chr8_6721995 | 8 | Pm025960 | days_25pct&days_75pct | days_25pct&days_75pct | intergenic | . | . | PF11960:Domain of unknown function (DUF3474)\|PF00487:Fatty acid desaturase |
| Chr8_7664971 | 8 | Pm026088 | days_10st_flower&days_5pct | days_10st_flower&days_5pct | intergenic | . | . | - |
| Chr8_8048127 | 8 | Pm026136 | days_25pct | days_25pct | downstream | . | . | PF00232:Glycosyl hydrolase family 1 |
| Chr8_8352121 | 8 | Pm026194 | days_25pct | days_25pct | downstream | . | . | PF14008:Iron/zinc purple acid phosphatase-like protein C\|PF00149:Calcineurin-like phosphoesterase\|PF16656:Purple acid Phosphatase, N-terminal domain |
| Chr8_8376764 | 8 | Pm026199 | days_1st_flower&days_10st_flower&days_5pct&days_25pct | days_1st_flower&days_10st_flower&days_5pct&days_25pct | upstream | . | . | PF01156:Inosine-uridine preferring nucleoside hydrolase |
| Chr8_8659741 | 8 | Pm026237 | days_1st_flower | days_1st_flower | intergenic | . | . | PF06974:Protein of unknown function (DUF1298)\|PF03007:Wax ester synthase-like Acyl-CoA acyltransferase domain |
| Chr8_9457301 | 8 | Pm026365 | days_10st_flower | days_10st_flower | downstream | . | . | PF06405:Red chlorophyll catabolite reductase (RCC reductase) |
| Chr8_9457307 | 8 | Pm026365 | days_10st_flower | days_10st_flower | downstream | . | . | PF06405:Red chlorophyll catabolite reductase (RCC reductase) |
| Chr8_9766524 | 8 | Pm026423 | days_10st_flower | days_10st_flower | upstream | . | . | PF01734:Patatin-like phospholipase |
| Chr8_9802197 | 8 | Pm026430 | days_10st_flower | days_10st_flower | intergenic | . | . | PF12371:Transmembrane protein 131-like |

**Table S4**. Selected top candidates associated with blooming-time related traits from gene-based association tests. Some candidate genes were found related to multiple traits.

| Gene | Year | Trait | Chr | Start | Stop | Pfam_description |
| --- | --- | --- | --- | --- | --- | --- |
| Pm000028 | 2019 | 5pct flowering&25pct flowering&75pct flowering | 1 | 162495 | 168946 | PF13520:Amino acid permease |
| Pm000029 | 2019 | 5pct flowering&25pct flowering&75pct flowering | 1 | 166614 | 173044 | PF13520:Amino acid permease |
| Pm000030 | 2019 | 5pct flowering&25pct flowering&75pct flowering | 1 | 168866 | 175317 | PF13520:Amino acid permease |
| Pm000036 | 2019 | first ten flower&5pct flowering&75pct flowering | 1 | 210467 | 218439 | PF01344:Kelch motif |
| Pm000038 | 2019 | first ten flower&5pct flowering&75pct flowering | 1 | 217344 | 222859 | PF04398:Protein of unknown function, DUF538 |
| Pm000053 | 2019 | 25pct flowering&75pct flowering | 1 | 377154 | 383827 | PF01535:PPR repeat\|PF13041:PPR repeat family |
| Pm000096 | 2019 | 5pct flowering&25pct flowering&75pct flowering | 1 | 712602 | 719803 | PF13855:Leucine rich repeat |
| Pm000098 | 2019 | 75pct flowering | 1 | 721711 | 727539 | PF14368:Probable lipid transfer |
| Pm000158 | 2017 | first flower&75pct flowering | 1 | 1041514 | 1047822 | PF00067:Cytochrome P450 |
| Pm000163 | 2019 | 25pct flowering | 1 | 1067780 | 1075453 | PF03106:WRKY DNA -binding domain |
| Pm000212 | 2019 | 25pct flowering | 1 | 1343287 | 1351896 | PF00067:Cytochrome P450 |
| Pm000213 | 2019 | 25pct flowering | 1 | 1356772 | 1364473 | PF00067:Cytochrome P450 |
| Pm000246 | 2019 | 25pct flowering&75pct flowering | 1 | 1532679 | 1539214 | PF00072:Response regulator receiver domain |
| Pm000251 | 2019 | 25pct flowering | 1 | 1558218 | 1565637 | PF00170:bZIP transcription factor |
| Pm000305 | 2019 | 5pct flowering&25pct flowering&75pct flowering | 1 | 1918726 | 1927358 | PF13855:Leucine rich repeat\|PF08263:Leucine rich repeat N-terminal domain\|PF00069:Protein kinase domain |
| Pm000312 | 2019 | 25pct flowering&75pct flowering | 1 | 1956475 | 1971389 | PF13414:TPR repeat\|PF00515:Tetratricopeptide repeat |
| Pm000370 | 2019 | 5pct flowering | 1 | 2306488 | 2314895 | PF08263:Leucine rich repeat N-terminal domain\|PF00069:Protein kinase domain\|PF13855:Leucine rich repeat |
| Pm000390 | 2019 | first ten flower&5pct flowering | 1 | 2415771 | 2421243 | PF00403:Heavy-metal-associated domain |
| Pm000407 | 2019 | first ten flower&5pct flowering&75pct flowering | 1 | 2516465 | 2522139 | PF00013:KH domain |
| Pm000420 | 2019 | first ten flower&5pct flowering | 1 | 2593264 | 2599507 | PF00249:Myb-like DNA-binding domain |
| Pm000537 | 2019 | 5pct flowering | 1 | 3323681 | 3330730 | PF00067:Cytochrome P450 |
| Pm000543 | 2017&2019 | first ten flower&25pct flowering | 1 | 3371785 | 3380586 | PF09079:CDC6, C terminal\|PF13401:AAA domain |
| Pm000550 | 2019 | first flower&first ten flower | 1 | 3408921 | 3416137 | PF13041:PPR repeat family\|PF01535:PPR repeat\|PF14432:DYW family of nucleic acid deaminases |
| Pm000551 | 2019 | first flower&first ten flower | 1 | 3411709 | 3418411 | PF00686:Starch binding domain |
| Pm000555 | 2019 | first flower&first ten flower | 1 | 3426655 | 3434590 | PF04185:Phosphoesterase family |
| Pm000633 | 2017 | 75pct flowering | 1 | 3888305 | 3894821 | PF00201:UDP-glucoronosyl and UDP-glucosyl transferase |
| Pm000637 | 2019 | first ten flower | 1 | 3917235 | 3923724 | PF00201:UDP-glucoronosyl and UDP-glucosyl transferase |
| Pm000692 | 2017 | first flower&5pct flowering&25pct flowering&75pct flowering | 1 | 4209023 | 4215669 | PF13041:PPR repeat family |
| Pm000774 | 2017&2019 | first ten flower&25pct flowering&75pct flowering | 1 | 4699436 | 4708461 | - |
| Pm000800 | 2017&2019 | first ten flower&25pct flowering | 1 | 4888148 | 4895128 | PF00190:Cupin |
| Pm000892 | 2017 | first flower&75pct flowering | 1 | 5375249 | 5383170 | PF01429:Methyl-CpG binding domain |
| Pm000895 | 2017&2019 | first ten flower&75pct flowering | 1 | 5392838 | 5400590 | PF00121:Triosephosphate isomerase |
| Pm000994 | 2019 | 25pct flowering&75pct flowering | 1 | 6191722 | 6202589 | PF02362:B3 DNA binding domain\|PF06507:Auxin response factor |
| Pm001010 | 2017 | 25pct flowering | 1 | 6325441 | 6330932 | PF00808:Histone-like transcription factor (CBF/NF-Y) and archaeal histone |
| Pm001034 | 2017 | 25pct flowering | 1 | 6487409 | 6492909 | PF04520:Senescence regulator |
| Pm001136 | 2017 | 25pct flowering | 1 | 7374091 | 7379987 | PF02365:No apical meristem (NAM) protein |
| Pm001462 | 2017 | 25pct flowering&75pct flowering | 1 | 10332980 | 10341690 | PF13962:Domain of unknown function |
| Pm002200 | 2017 | 75pct flowering | 1 | 17648560 | 17654898 | PF00249:Myb-like DNA-binding domain |
| Pm002227 | 2017 | 75pct flowering | 1 | 17832998 | 17841292 | PF00069:Protein kinase domain |
| Pm002237 | 2017 | 75pct flowering | 1 | 17934184 | 17941319 | PF13041:PPR repeat family\|PF14432:DYW family of nucleic acid deaminases |
| Pm004169 | 2017 | first flower&5pct flowering&25pct flowering | 2 | 2996398 | 3004508 | PF08372:Plant phosphoribosyltransferase C-terminal\|PF00168:C2 domain |
| Pm004198 | 2017 | first flower&5pct flowering&25pct flowering | 2 | 3169659 | 3181086 | PF10250:GDP-fucose protein O-fucosyltransferase |
| Pm004199 | 2017 | 5pct flowering | 2 | 3178460 | 3184828 | PF00249:Myb-like DNA-binding domain |
| Pm004220 | 2017 | first flower&5pct flowering&25pct flowering | 2 | 3311256 | 3318578 | PF01738:Dienelactone hydrolase family |
| Pm004226 | 2017 | first flower&5pct flowering&25pct flowering | 2 | 3332174 | 3340336 | PF00724:NADH:flavin oxidoreductase / NADH oxidase family |
| Pm004227 | 2017 | first flower&5pct flowering&25pct flowering | 2 | 3337227 | 3345834 | PF00724:NADH:flavin oxidoreductase / NADH oxidase family |
| Pm004228 | 2017 | first flower&5pct flowering&25pct flowering | 2 | 3342079 | 3349349 | PF00724:NADH:flavin oxidoreductase / NADH oxidase family |
| Pm004245 | 2017 | first flower&5pct flowering | 2 | 3470782 | 3478101 | PF00657:GDSL-like Lipase/Acylhydrolase |
| Pm004248 | 2017 | first flower&5pct flowering | 2 | 3481838 | 3491287 | PF00657:GDSL-like Lipase/Acylhydrolase |
| Pm004256 | 2017 | first flower&5pct flowering | 2 | 3568690 | 3577078 | PF01490:Transmembrane amino acid transporter protein |
| Pm004260 | 2017 | first flower&5pct flowering | 2 | 3594705 | 3604605 | PF00069:Protein kinase domain |
| Pm004261 | 2017 | first flower&5pct flowering | 2 | 3601785 | 3609786 | PF00520:Ion transport protein |
| Pm004272 | 2017&2019 | first flower&5pct flowering | 2 | 3667403 | 3673002 | PF00847:AP2 domain |
| Pm004303 | 2017 | first ten flower&5pct flowering | 2 | 3872746 | 3885741 | PF12394:Protein of unknown function (DUF3657)\|PF05057:Putative serine esterase (DUF676) |
| Pm004349 | 2017&2019 | first flower&first ten flower&5pct flowering&25pct flowering&75pct flowering | 2 | 4210007 | 4232433 | PF00319:SRF-type transcription factor (DNA-binding and dimerisation domain)\|PF01486:K-box region |
| Pm004350 | 2017 | first ten flower | 2 | 4210048 | 4215802 | PF01486:K-box region |
| Pm004368 | 2017&2019 | first flower&first ten flower&5pct flowering&25pct flowering | 2 | 4417224 | 4425196 | PF02751:Transcription initiation factor IIA, gamma subunit\|PF02268:Transcription initiation factor IIA, gamma subunit, helical domain |
| Pm004371 | 2017&2019 | first flower&first ten flower&5pct flowering | 2 | 4439081 | 4445685 | PF03171:2OG-Fe(II) oxygenase superfamily |
| Pm004376 | 2017&2019 | first flower&first ten flower&5pct flowering&25pct flowering | 2 | 4480417 | 4486932 | PF03171:2OG-Fe(II) oxygenase superfamily\|PF14226:non-haem dioxygenase in morphine synthesis N-terminal |
| Pm004415 | 2017&2019 | first flower&5pct flowering | 2 | 4808470 | 4821934 | PF01486:K-box region\|PF00319:SRF-type transcription factor (DNA-binding and dimerisation domain) |
| Pm004416 | 2017&2019 | first flower&5pct flowering | 2 | 4818032 | 4831077 | PF01486:K-box region\|PF00319:SRF-type transcription factor (DNA-binding and dimerisation domain) |
| Pm004417 | 2019 | first flower&first ten flower | 2 | 4827351 | 4839929 | PF01486:K-box region\|PF00319:SRF-type transcription factor (DNA-binding and dimerisation domain) |
| Pm004418 | 2017&2019 | first flower&first ten flower&5pct flowering | 2 | 4836095 | 4850009 | PF01486:K-box region\|PF00319:SRF-type transcription factor (DNA-binding and dimerisation domain) |
| Pm004419 | 2019 | first ten flower | 2 | 4851303 | 4867823 | PF00319:SRF-type transcription factor (DNA-binding and dimerisation domain)\|PF01486:K-box region |
| Pm004529 | 2017&2019 | first flower&first ten flower&5pct flowering&25pct flowering | 2 | 5618254 | 5625322 | PF02984:Cyclin, C-terminal domain\|PF00134:Cyclin, N-terminal domain |
| Pm004616 | 2017&2019 | first flower&first ten flower&5pct flowering&25pct flowering | 2 | 6084807 | 6090692 | PF00847:AP2 domain |
| Pm004718 | 2017&2019 | first flower&first ten flower&5pct flowering&25pct flowering | 2 | 6742828 | 6748019 | PF01486:K-box region\|PF00319:SRF-type transcription factor (DNA-binding and dimerisation domain) |
| Pm004741 | 2017&2019 | first flower&first ten flower&5pct flowering | 2 | 6910237 | 6919619 | PF02984:Cyclin, C-terminal domain\|PF00134:Cyclin, N-terminal domain |
| Pm004870 | 2017&2019 | first flower&first ten flower&5pct flowering&25pct flowering&75pct flowering | 2 | 7715291 | 7721031 | PF00847:AP2 domain |
| Pm004913 | 2017&2019 | first flower&first ten flower&5pct flowering&25pct flowering | 2 | 8071059 | 8076376 | - |
| Pm004966 | 2017&2019 | first flower&5pct flowering | 2 | 8374488 | 8380862 | PF03171:2OG-Fe(II) oxygenase superfamily\|PF14226:non-haem dioxygenase in morphine synthesis N-terminal |
| Pm005134 | 2017&2019 | first flower&first ten flower&5pct flowering&25pct flowering | 2 | 9388028 | 9398490 | - |
| Pm005214 | 2017&2019 | first flower&first ten flower&5pct flowering&25pct flowering&75pct flowering | 2 | 9839397 | 9845684 | PF14226:non-haem dioxygenase in morphine synthesis N-terminal\|PF03171:2OG-Fe(II) oxygenase superfamily |
| Pm005288 | 2017&2019 | first flower&first ten flower&5pct flowering | 2 | 10206939 | 10215083 | PF00170:bZIP transcription factor |
| Pm005326 | 2017&2019 | first flower&first ten flower&5pct flowering&25pct flowering | 2 | 10444217 | 10450690 | PF02984:Cyclin, C-terminal domain\|PF00134:Cyclin, N-terminal domain |
| Pm005349 | 2017&2019 | first flower&first ten flower&5pct flowering&25pct flowering | 2 | 10557542 | 10564626 | PF00134:Cyclin, N-terminal domain\|PF02984:Cyclin, C-terminal domain |
| Pm005440 | 2017&2019 | first flower&first ten flower&5pct flowering&25pct flowering | 2 | 11031536 | 11039603 | PF00847:AP2 domain |
| Pm005708 | 2017&2019 | first flower&first ten flower&5pct flowering&25pct flowering | 2 | 12507011 | 12524204 | PF00271:Helicase conserved C-terminal domain\|PF00385:Chromo (CHRromatin Organisation MOdifier) domain\|PF06461:Domain of Unknown Function (DUF1086)\|PF06465:Domain of Unknown Function (DUF1087)\|PF00176:SNF2 family N-terminal domain\|PF00628:PHD-finger |
| Pm006266 | 2017&2019 | first flower&first ten flower&5pct flowering&25pct flowering | 2 | 15793811 | 15800737 | PF01486:K-box region\|PF00319:SRF-type transcription factor (DNA-binding and dimerisation domain) |
| Pm006304 | 2017 | first flower&first ten flower&5pct flowering&25pct flowering&75pct flowering | 2 | 16003107 | 16010008 | PF03514:GRAS domain family\|PF12041:Transcriptional regulator DELLA protein N terminal |
| Pm006485 | 2017 | first flower&first ten flower&5pct flowering&25pct flowering&75pct flowering | 2 | 17091397 | 17099082 | PF02701:Dof domain, zinc finger |
| Pm006520 | 2017 | first ten flower | 2 | 17288733 | 17300098 | PF00415:Regulator of chromosome condensation (RCC1) repeat\|PF13713:Transcription factor BRX N-terminal domain\|PF01363:FYVE zinc finger\|PF08381:Transcription factor regulating root and shoot growth via Pin3 |
| Pm006530 | 2017 | first ten flower | 2 | 17357170 | 17363643 | PF00046:Homeobox domain\|PF02183:Homeobox associated leucine zipper |
| Pm006615 | 2017 | first ten flower | 2 | 17933364 | 17943723 | PF00931:NB-ARC domain\|PF01582:TIR domain |
| Pm006670 | 2017 | first ten flower | 2 | 18324430 | 18331951 | PF04055:Radical SAM superfamily |
| Pm006700 | 2017 | first ten flower | 2 | 18524084 | 18536127 | PF00295:Glycosyl hydrolases family 28\|PF00378:Enoyl-CoA hydratase/isomerase |
| Pm010412 | 2019 | first ten flower&25pct flowering&75pct flowering | 3 | 4305916 | 4312413 | PF14226:non-haem dioxygenase in morphine synthesis N-terminal\|PF03171:2OG-Fe(II) oxygenase superfamily |
| Pm011391 | 2017 | 5pct flowering | 3 | 10774347 | 10780003 | PF00847:AP2 domain |
| Pm014207 | 2019 | first flower | 4 | 10534594 | 10540849 | PF00891:O-methyltransferase\|PF08100:Dimerisation domain |
| Pm018083 | 2019 | first flower&25pct flowering&75pct flowering | 5 | 16078597 | 16085100 | PF03171:2OG-Fe(II) oxygenase superfamily\|PF14226:non-haem dioxygenase in morphine synthesis N-terminal |
| Pm018089 | 2017&2019 | 75pct flowering | 5 | 16112977 | 16122279 | PF01486:K-box region\|PF00319:SRF-type transcription factor (DNA-binding and dimerisation domain) |
| Pm019119 | 2017&2019 | first flower&first ten flower&5pct flowering | 5 | 22248000 | 22257968 | PF01853:MOZ/SAS family\|PF11717:RNA binding activity-knot of a chromodomain |
| Pm019832 | 2019 | first flower | 5 | 25984120 | 25989999 | PF03106:WRKY DNA -binding domain |
| Pm019848 | 2019 | first flower | 5 | 26047809 | 26055272 | PF00295:Glycosyl hydrolases family 28 |
| Pm022777 | 2019 | first flower | 6 | 20556369 | 20564258 | PF03171:2OG-Fe(II) oxygenase superfamily\|PF14226:non-haem dioxygenase in morphine synthesis N-terminal |
| Pm024524 | 2017 | first flower | 7 | 12844400 | 12850802 | PF01486:K-box region\|PF00319:SRF-type transcription factor (DNA-binding and dimerisation domain) |
| Pm026136 | 2019 | first flower | 8 | 8042884 | 8050109 | PF00232:Glycosyl hydrolase family 1 |

**Table S5**. Summary statistics of transcriptome sequencing on twenty-seven floral bud samples from two *P. mume* cultivars.

| Sample | Raw  reads | Clean  reads | Q20 | Q30 | GC% | Total  mapping rate | Unique  Mapping rate |
| --- | --- | --- | --- | --- | --- | --- | --- |
| FZ_S11 | 56279524 | 55243116 | 97.76 | 93.54 | 45.03 | 94.07% | 91.63% |
| FZ_S12 | 58097318 | 57183792 | 97.83 | 93.73 | 44.79 | 93.65% | 91.22% |
| FZ_S13 | 50820706 | 50045594 | 97.81 | 93.67 | 45.34 | 94.05% | 91.49% |
| FZ_S21 | 51750002 | 51070306 | 97.73 | 93.54 | 45.02 | 93.51% | 90.90% |
| FZ_S22 | 50094942 | 49272990 | 97.77 | 93.58 | 44.77 | 93.57% | 91.07% |
| FZ_S23 | 59874906 | 58805868 | 97.77 | 93.6 | 44.9 | 93.98% | 91.34% |
| FZ_S31 | 55255706 | 54209392 | 97.59 | 93.23 | 44.7 | 93.12% | 90.59% |
| FZ_S32 | 57694952 | 56670062 | 97.68 | 93.39 | 44.82 | 93.67% | 91.05% |
| FZ_S33 | 50616576 | 49731082 | 97.81 | 93.73 | 44.63 | 93.18% | 90.65% |
| FZ_S41 | 59927682 | 58939194 | 97.72 | 93.49 | 44.61 | 82.27% | 80.00% |
| FZ_S42 | 65424292 | 64409680 | 97.6 | 93.22 | 44.49 | 67.35% | 65.54% |
| FZ_S43 | 49900158 | 49170664 | 97.79 | 93.71 | 44.76 | 79.71% | 77.49% |
| ST_S01 | 46441912 | 45734370 | 97.89 | 93.87 | 45.41 | 92.52% | 90.06% |
| ST_S02 | 43409266 | 42447174 | 97.86 | 93.78 | 45.45 | 92.45% | 90.01% |
| ST_S03 | 53466854 | 52603726 | 97.67 | 93.37 | 45.25 | 91.91% | 89.46% |
| ST_S11 | 45215848 | 44556838 | 97.86 | 93.82 | 44.95 | 91.67% | 89.21% |
| ST_S12 | 48694718 | 47992056 | 97.76 | 93.56 | 44.52 | 87.95% | 85.65% |
| ST_S13 | 51807126 | 50878246 | 97.84 | 93.77 | 44.88 | 91.68% | 89.22% |
| ST_S21 | 53430458 | 52272096 | 97.36 | 92.61 | 45.42 | 92.36% | 89.60% |
| ST_S22 | 45621364 | 44888196 | 97.71 | 93.44 | 44.9 | 91.30% | 88.80% |
| ST_S23 | 55234770 | 54465776 | 97.62 | 93.26 | 45 | 91.58% | 88.97% |
| ST_S31 | 43774766 | 43095944 | 97.75 | 93.57 | 45.04 | 90.18% | 87.34% |
| ST_S32 | 56526916 | 55633822 | 97.78 | 93.61 | 45.15 | 92.87% | 89.89% |
| ST_S33 | 43553158 | 42874526 | 97.7 | 93.44 | 45.31 | 92.93% | 89.97% |
| ST_S41 | 50715062 | 49871602 | 97.77 | 93.64 | 45.26 | 83.97% | 81.59% |
| ST_S42 | 46903146 | 46189818 | 97.74 | 93.57 | 45.24 | 83.77% | 81.34% |
| ST_S43 | 63483978 | 62585772 | 97.84 | 93.75 | 45.15 | 78.64% | 76.28% |

**Table S6**. The GO enrichment analysis of the 191 associated candidate genes.

| Category | GOID | Description | Gene Ratio | P-value | Adjusted-Pvalue |
| --- | --- | --- | --- | --- | --- |
| BP | GO:0044281 | small molecule metabolic process | 8/73 | 0.033 | 0.607 |
| BP | GO:0019752 | carboxylic acid metabolic process | 6/73 | 0.034 | 0.607 |
| BP | GO:0043436 | oxoacid metabolic process | 6/73 | 0.036 | 0.607 |
| BP | GO:0006082 | organic acid metabolic process | 6/73 | 0.036 | 0.607 |
| BP | GO:0016485 | protein processing | 1/73 | 0.083 | 0.607 |
| BP | GO:0015696 | ammonium transport | 1/73 | 0.091 | 0.607 |
| BP | GO:0009073 | aromatic amino acid family biosynthetic process | 1/73 | 0.099 | 0.607 |
| BP | GO:0009719 | response to endogenous stimulus | 2/73 | 0.102 | 0.607 |
| BP | GO:0009725 | response to hormone | 2/73 | 0.102 | 0.607 |
| BP | GO:0010033 | response to organic substance | 2/73 | 0.102 | 0.607 |
| BP | GO:0006006 | glucose metabolic process | 1/73 | 0.106 | 0.607 |
| BP | GO:0019751 | polyol metabolic process | 1/73 | 0.122 | 0.607 |
| BP | GO:0051604 | protein maturation | 1/73 | 0.122 | 0.607 |
| BP | GO:0006066 | alcohol metabolic process | 1/73 | 0.129 | 0.607 |
| BP | GO:0006334 | nucleosome assembly | 1/73 | 0.137 | 0.607 |
| BP | GO:0031497 | chromatin assembly | 1/73 | 0.137 | 0.607 |
| BP | GO:0034728 | nucleosome organization | 1/73 | 0.137 | 0.607 |
| BP | GO:0008272 | sulfate transport | 1/73 | 0.144 | 0.607 |
| BP | GO:0072348 | sulfur compound transport | 1/73 | 0.144 | 0.607 |
| BP | GO:0006323 | DNA packaging | 1/73 | 0.152 | 0.607 |
| BP | GO:0006333 | chromatin assembly or disassembly | 1/73 | 0.152 | 0.607 |
| BP | GO:0065004 | protein-DNA complex assembly | 1/73 | 0.152 | 0.607 |
| BP | GO:0071824 | protein-DNA complex subunit organization | 1/73 | 0.152 | 0.607 |
| BP | GO:0019318 | hexose metabolic process | 1/73 | 0.159 | 0.607 |
| BP | GO:0044282 | small molecule catabolic process | 1/73 | 0.166 | 0.607 |
| BP | GO:1901615 | organic hydroxy compound metabolic process | 1/73 | 0.166 | 0.607 |
| BP | GO:0009072 | aromatic amino acid family metabolic process | 1/73 | 0.173 | 0.610 |
| BP | GO:0005996 | monosaccharide metabolic process | 1/73 | 0.188 | 0.637 |
| BP | GO:0009308 | amine metabolic process | 1/73 | 0.202 | 0.660 |
| BP | GO:0071103 | DNA conformation change | 1/73 | 0.215 | 0.682 |
| BP | GO:0045017 | glycerolipid biosynthetic process | 1/73 | 0.242 | 0.735 |
| BP | GO:0006950 | response to stress | 4/73 | 0.254 | 0.735 |
| BP | GO:0015698 | inorganic anion transport | 1/73 | 0.255 | 0.735 |
| BP | GO:0044262 | cellular carbohydrate metabolic process | 2/73 | 0.280 | 0.751 |
| BP | GO:0006325 | chromatin organization | 1/73 | 0.293 | 0.751 |
| BP | GO:0006486 | protein glycosylation | 1/73 | 0.293 | 0.751 |
| BP | GO:0043413 | macromolecule glycosylation | 1/73 | 0.293 | 0.751 |
| BP | GO:0009100 | glycoprotein metabolic process | 1/73 | 0.317 | 0.751 |
| BP | GO:0009101 | glycoprotein biosynthetic process | 1/73 | 0.317 | 0.751 |
| BP | GO:0070085 | glycosylation | 1/73 | 0.317 | 0.751 |
| BP | GO:0006520 | cellular amino acid metabolic process | 2/73 | 0.331 | 0.751 |
| BP | GO:0009733 | response to auxin | 1/73 | 0.335 | 0.751 |
| BP | GO:0008652 | cellular amino acid biosynthetic process | 1/73 | 0.340 | 0.751 |
| BP | GO:0042221 | response to chemical | 2/73 | 0.356 | 0.751 |
| BP | GO:0046486 | glycerolipid metabolic process | 1/73 | 0.357 | 0.751 |
| BP | GO:0016052 | carbohydrate catabolic process | 1/73 | 0.374 | 0.751 |
| BP | GO:0034622 | cellular protein-containing complex assembly | 1/73 | 0.374 | 0.751 |
| BP | GO:0065003 | protein-containing complex assembly | 1/73 | 0.406 | 0.751 |
| BP | GO:0051276 | chromosome organization | 1/73 | 0.411 | 0.751 |
| BP | GO:0006928 | movement of cell or subcellular component | 1/73 | 0.416 | 0.751 |
| BP | GO:0007018 | microtubule-based movement | 1/73 | 0.416 | 0.751 |
| BP | GO:0044255 | cellular lipid metabolic process | 2/73 | 0.420 | 0.751 |
| BP | GO:0006631 | fatty acid metabolic process | 1/73 | 0.421 | 0.751 |
| BP | GO:0006952 | defense response | 1/73 | 0.455 | 0.751 |
| BP | GO:0043933 | protein-containing complex subunit organization | 1/73 | 0.460 | 0.751 |
| BP | GO:0006281 | DNA repair | 1/73 | 0.465 | 0.751 |
| BP | GO:0006974 | cellular response to DNA damage stimulus | 1/73 | 0.474 | 0.751 |
| BP | GO:0033554 | cellular response to stress | 1/73 | 0.474 | 0.751 |
| BP | GO:0044283 | small molecule biosynthetic process | 2/73 | 0.484 | 0.751 |
| BP | GO:0007017 | microtubule-based process | 1/73 | 0.501 | 0.751 |
| BP | GO:0006073 | cellular glucan metabolic process | 1/73 | 0.510 | 0.751 |
| BP | GO:0044042 | glucan metabolic process | 1/73 | 0.510 | 0.751 |
| BP | GO:0044264 | cellular polysaccharide metabolic process | 1/73 | 0.510 | 0.751 |
| BP | GO:0006820 | anion transport | 1/73 | 0.514 | 0.751 |
| BP | GO:0016051 | carbohydrate biosynthetic process | 1/73 | 0.514 | 0.751 |
| BP | GO:0022607 | cellular component assembly | 1/73 | 0.543 | 0.775 |
| BP | GO:0005976 | polysaccharide metabolic process | 1/73 | 0.547 | 0.775 |
| BP | GO:0006457 | protein folding | 1/73 | 0.555 | 0.775 |
| BP | GO:0006811 | ion transport | 3/73 | 0.601 | 0.822 |
| BP | GO:0006996 | organelle organization | 1/73 | 0.606 | 0.822 |
| BP | GO:0032787 | monocarboxylic acid metabolic process | 1/73 | 0.616 | 0.824 |
| BP | GO:0065008 | regulation of biological quality | 1/73 | 0.648 | 0.855 |
| BP | GO:0016192 | vesicle-mediated transport | 1/73 | 0.666 | 0.867 |
| BP | GO:0044085 | cellular component biogenesis | 1/73 | 0.700 | 0.883 |
| BP | GO:0008610 | lipid biosynthetic process | 1/73 | 0.705 | 0.883 |
| BP | GO:1901137 | carbohydrate derivative biosynthetic process | 1/73 | 0.708 | 0.883 |
| BP | GO:0006259 | DNA metabolic process | 1/73 | 0.735 | 0.883 |
| BP | GO:0016053 | organic acid biosynthetic process | 1/73 | 0.735 | 0.883 |
| BP | GO:0046394 | carboxylic acid biosynthetic process | 1/73 | 0.735 | 0.883 |
| BP | GO:0071705 | nitrogen compound transport | 1/73 | 0.753 | 0.894 |
| BP | GO:0006629 | lipid metabolic process | 2/73 | 0.768 | 0.900 |
| BP | GO:0051716 | cellular response to stimulus | 3/73 | 0.777 | 0.900 |
| BP | GO:0044248 | cellular catabolic process | 1/73 | 0.796 | 0.911 |
| BP | GO:1901575 | organic substance catabolic process | 1/73 | 0.822 | 0.918 |
| BP | GO:1901135 | carbohydrate derivative metabolic process | 1/73 | 0.831 | 0.918 |
| BP | GO:0009056 | catabolic process | 1/73 | 0.834 | 0.918 |
| BP | GO:0006812 | cation transport | 1/73 | 0.860 | 0.918 |
| BP | GO:0007165 | signal transduction | 2/73 | 0.860 | 0.918 |
| BP | GO:0023052 | signaling | 2/73 | 0.860 | 0.918 |
| BP | GO:0016043 | cellular component organization | 1/73 | 0.878 | 0.927 |
| BP | GO:0006508 | proteolysis | 2/73 | 0.888 | 0.927 |
| BP | GO:0007154 | cell communication | 2/73 | 0.919 | 0.940 |
| BP | GO:0071840 | cellular component organization or biogenesis | 1/73 | 0.921 | 0.940 |
| BP | GO:0006518 | peptide metabolic process | 1/73 | 0.952 | 0.960 |
| BP | GO:0043603 | cellular amide metabolic process | 1/73 | 0.960 | 0.960 |

**Table S7**. Primers used in qRT-PCR analysis.

| Gene | Primer name | Sequence |
| --- | --- | --- |
| PmPP2A | PP2A-F | AGGGTTCGGCTCGCAATAATAGA |
|  | PP2A-R | TGTTAGCAGCAGCATCACGAAT |
| PmDAM4 | DAM4-F | ACCCTTGTCCGTGTGATGGAA |
|  | DAM4-R | ATCACCATCTGATTGTTGCCT |
| PmDAM5 | DAM5-F | CGCAGACATGAATGGTGTCG |
|  | DAM5-R | CGTCTTCTCAAGTTCCTTACTCAG |
| PmDAM6 | DAM6-F | AACCAACAACCAGTTAAGGCATA |
|  | DAM6-R | CAATTACGGCAGATTCAGATGA |
| Pm002475 | Pm002475-F | TCATTGCCAGCCTACGGTTT |
|  | Pm002475-R | GGCAGCCAAAGACACACAAG |
| Pm004574 | Pm004574-F | GGTTCTGTTGCGTTGACAAAGA |
|  | Pm004574-R | TTCAGCATTGGGGTTATGTGAC |
| Pm004575 | Pm004575-F | CCTTCAAGGCGCTTTTGACC |
|  | Pm004575-R | CTCTGCTGCATCAAGGGTGA |
| Pm004966 | Pm004966-F | AGGCCTTACGATCTCCAGGT |
|  | Pm004966-R | GAGGCCAAAGTTGCCTCGTA |
| Pm005276 | Pm005276-F | TGCTTTTGCCTCTGCGTTTC |
|  | Pm005276-R | GGGAATGGTGGTCTTGAGGG |
| Pm005396 | Pm005396-F | TGCCAAGAATCTCCACCAGG |
|  | Pm005396-R | AGCTGCTATGGCAGATGGTC |
| Pm006215 | Pm006215-F | GATGCAATTGCGTTCCACCA |
|  | Pm006215-R | ATTTGTGTAGGCCCCTGTGG |
| Pm018083 | Pm018083-F | GTACCCGGACTTTACCTGGC |
|  | Pm018083-R | AGAAGGCATCCAGCGTCTTC |
| Pm021881 | Pm021881-F | TCCGGTTGCCTGGAATTGTT |
|  | Pm021881-R | GCTTCATTTGGCTCTCCCCA |
| Pm024587 | Pm024587-F | GTTGTGGAGAGCAAGGACGA |
|  | Pm024587-R | GCGAGAAGGCTATGTCTCGG |

**Table S8.** The 191 candidate genes displaying consistent expression pattern during blooming across different *P. mume* cultivars.

| ID | Module 'FZ' | Module 'ST' | Pfam_description | Arabidopsis homolog | Gene_name |
| --- | --- | --- | --- | --- | --- |
| Pm000225 | MEblue | MEblue | PF00651:BTB/POZ domain\|PF03000:NPH3 family | AT2G30520.1 | RPT2 (ROOT PHOTOTROPISM 2) |
| Pm004456 | MEblue | MEblue | PF04674:Phosphate-induced protein 1 conserved region | AT4G08950.1 | EXO (EXORDIUM) |
| Pm004461 | MEblue | MEblue | PF01474:Class-II DAHP synthetase family | AT1G22410.1 | Class-II DAHP synthetase family protein |
| Pm004773 | MEblue | MEblue | PF01546:Peptidase family M20/M25/M40 | AT1G44350.1 | ILL6 (IAA-LEUCINE RESISTANT-LIKE GENE 6) |
| Pm005112 | MEblue | MEblue | PF00743:Flavin-binding monooxygenase-like | AT5G25620.1 | YUC6 (YUCCA6) |
| Pm005143 | MEblue | MEblue | PF07250:Glyoxal oxidase N-terminus\|PF09118:Domain of unknown function (DUF1929) | AT5G19580.1 | Glyoxal oxidase-related protein |
| Pm005359 | MEblue | MEblue | PF00067:Cytochrome P450 | AT1G66540.1 | Cytochrome P450 superfamily protein |
| Pm005664 | MEblue | MEblue | - | AT2G24860.1 | EIJ1 (EDS1 -INTERACTING J PROTEIN 1) |
| Pm006112 | MEblue | MEblue | PF01490:Transmembrane amino acid transporter protein | AT1G47670.1 | Transmembrane amino acid transporter family protein |
| Pm006149 | MEblue | MEblue | - | - | - |
| Pm006492 | MEblue | MEblue | PF00445:Ribonuclease T2 family | AT1G14220.1 | Ribonuclease T2 family protein |
| Pm006510 | MEblue | MEblue | - | AT1G14280.1 | PKS2 (PHYTOCHROME KINASE SUBSTRATE 2) |
| Pm006909 | MEblue | MEblue | PF12056:Protein of unknown function (DUF3537) | AT1G67570.1 | - |
| Pm009766 | MEblue | MEblue | PF00069:Protein kinase domain | AT3G20530.1 | PBL23 (PBS1-LIKE 23) |
| Pm009954 | MEblue | MEblue | - | - | - |
| Pm010242 | MEblue | MEblue | PF03822:NAF domain\|PF00069:Protein kinase domain | AT5G45820.1 | CIPK20 (CBL-INTERACTING PROTEIN KINASE 20) |
| Pm010421 | MEblue | MEblue | - | - | - |
| Pm010425 | MEblue | MEblue | PF03055:Retinal pigment epithelial membrane protein | AT1G30100.1 | NCED5 (NINE-CIS-EPOXYCAROTENOID DIOXYGENASE 5) |
| Pm014361 | MEblue | MEblue | - | AT3G03440.1 | ARM repeat superfamily protein |
| Pm017952 | MEblue | MEblue | PF00067:Cytochrome P450 | AT2G45510.1 | CYP704A2 |
| Pm019810 | MEblue | MEblue | - | - | - |
| Pm020574 | MEblue | MEblue | PF00005:ABC transporter | AT1G03905.1 | ABCI19 (ATP-BINDING CASSETTE I19) |
| Pm021881 | MEblue | MEblue | PF02519:Auxin responsive protein | AT4G38840.1 | SAUR14(SMALL AUXIN UPREGULATED RNA 14) |
| Pm021894 | MEblue | MEblue | - | AT4G38840.1 | SAUR14(SMALL AUXIN UPREGULATED RNA 14) |
| Pm021946 | MEblue | MEblue | - | - | - |
| Pm022756 | MEblue | MEblue | PF08263:Leucine rich repeat N-terminal domain\|PF13855:Leucine rich repeat | AT1G33590.1 | Leucine-rich repeat (LRR) family protein |
| Pm004227 | MEbrown | MEblue | PF00724:NADH:flavin oxidoreductase / NADH oxidase family | AT1G76690.1 | OPR2 (12-OXOPHYTODIENOATE REDUCTASE 2) |
| Pm004325 | MEbrown | MEblue | PF01293:Phosphoenolpyruvate carboxykinase | AT4G37870.1 | PCK1 (PHOSPHOENOLPYRUVATE CARBOXYKINASE 1) |
| Pm004874 | MEbrown | MEblue | PF00892:EamA-like transporter family | AT4G08290.1 | UMAMIT20 (USUALLY MULTIPLE ACIDS MOVE IN AND OUT TRANSPORTERS 20) |
| Pm005158 | MEbrown | MEblue | PF00415:Regulator of chromosome condensation (RCC1) repeat | AT5G11580.1 | Regulator of chromosome condensation (RCC1) family protein |
| Pm005276 | MEbrown | MEblue | PF07821:Alpha-amylase C-terminal beta-sheet domain\|PF00128:Alpha amylase, catalytic domain | AT4G25000.1 | AMY1 (ALPHA-AMYLASE-LIKE) |
| Pm006215 | MEbrown | MEblue | PF02704:Gibberellin regulated protein | AT2G18420.1 | Gibberellin-regulated family protein |
| Pm006576 | MEbrown | MEblue | PF00854:POT family | AT1G69850.1 | AIT1 (ABA-IMPORTING TRANSPORTER 1) |
| Pm007059 | MEbrown | MEblue | PF02179:BAG domain | AT1G12060.1 | BAG5 (BCL-2-ASSOCIATED ATHANOGENE 5) |
| Pm009471 | MEbrown | MEblue | PF00173:Cytochrome b5-like Heme/Steroid binding domain | AT2G32720.1 | - |
| Pm010009 | MEbrown | MEblue | - | AT5G53750.1 | CBS domain-containing protein |
| Pm010422 | MEbrown | MEblue | PF01344:Kelch motif | AT1G30090.1 | Galactose oxidase/kelch repeat superfamily protein;(source:Araport11) |
| Pm010474 | MEbrown | MEblue | PF00282:Pyridoxal-dependent decarboxylase conserved domain | AT2G20340.1 | Pyridoxal phosphate (PLP)-dependent transferases superfamily protein |
| Pm010480 | MEbrown | MEblue | PF00282:Pyridoxal-dependent decarboxylase conserved domain | AT2G20340.1 | Pyridoxal phosphate (PLP)-dependent transferases superfamily protein |
| Pm010482 | MEbrown | MEblue | PF00282:Pyridoxal-dependent decarboxylase conserved domain | AT4G28680.1 | Decarboxylase |
| Pm010603 | MEbrown | MEblue | PF08031:Berberine and berberine like\|PF01565:FAD binding domain | AT1G30700.1 | FAD-binding Berberine family protein |
| Pm011547 | MEbrown | MEblue | - | AT3G15352.1 | Cytochrome c oxidase 17 |
| Pm018998 | MEbrown | MEblue | PF03208:PRA1 family protein | AT3G56110.1 | Prenylated RAB acceptor 1.B1 |
| Pm019156 | MEbrown | MEblue | - | - | - |
| Pm019185 | MEbrown | MEblue | PF00916:Sulfate permease family\|PF01740:STAS domain | AT1G22150.1 | Sulfate transporter 1;3 |
| Pm019845 | MEbrown | MEblue | PF00183:Hsp90 protein\|PF02518:Histidine kinase-, DNA gyrase B-, and HSP90-like ATPase | AT5G52640.1 | HSP90.1 (HEAT SHOCK PROTEIN 90-1) |
| Pm020594 | MEbrown | MEblue | PF05553:Cotton fibre expressed protein | - | - |
| Pm022658 | MEbrown | MEblue | - | AT1G78780.1 | Pathogenesis-related family protein |
| Pm025999 | MEbrown | MEblue | PF08263:Leucine rich repeat N-terminal domain | AT5G06860.1 | PGIP1 (POLYGALACTURONASE INHIBITING PROTEIN 1) |
| Pm026119 | MEbrown | MEblue | PF10433:Mono-functional DNA-alkylating methyl methanesulfonate N-term\|PF03178:CPSF A subunit region | AT3G11960.1 | Cleavage and polyadenylation specificity factor (CPSF) A subunit protein |
| Pm000163 | MEturquoise | MEblue | PF03106:WRKY DNA -binding domain | AT2G38470.1 | WRKY33 (WRKY DNA-BINDING PROTEIN 33) |
| Pm000649 | MEturquoise | MEblue | PF00534:Glycosyl transferases group 1\|PF13439:Glycosyltransferase Family 4 | AT5G59070.1 | UDP-Glycosyltransferase superfamily protein |
| Pm000724 | MEturquoise | MEblue | - | AT5G59400.1 | PGR5-like A protein |
| Pm000923 | MEturquoise | MEblue | PF12697:Alpha/beta hydrolase family | AT5G02970.1 | - |
| Pm004042 | MEturquoise | MEblue | - | - | - |
| Pm004418 | MEturquoise | MEblue | PF01486:K-box region\|PF00319:SRF-type transcription factor (DNA-binding and dimerisation domain) |  | DAM3 (DORMANCY ASSOCIATED MADS-BOX 3) |
| Pm004476 | MEturquoise | MEblue | PF02183:Homeobox associated leucine zipper\|PF00046:Homeobox domain | AT2G22430.1 | HB6 (HOMEOBOX PROTEIN 6) |
| Pm004557 | MEturquoise | MEblue | PF00249:Myb-like DNA-binding domain | AT1G34670.1 | MYB93 (MYB DOMAIN PROTEIN 93) |
| Pm004574 | MEturquoise | MEblue | PF00201:UDP-glucoronosyl and UDP-glucosyl transferase | AT1G22360.1 | UDP-glucosyl transferase 85A2 (cytokinin-O-glucosyltransferase 2) |
| Pm004680 | MEturquoise | MEblue | - | - | - |
| Pm004855 | MEturquoise | MEblue | PF00083:Sugar (and other) transporter | AT1G34580.1 | Major facilitator superfamily protein |
| Pm004995 | MEturquoise | MEblue | - | AT2G25625.1 | CV (CHLOROPLAST VESICULATION) |
| Pm005221 | MEturquoise | MEblue | PF04759:Protein of unknown function, DUF617 | AT5G42680.1 | Protein of unknown function, DUF617 |
| Pm005277 | MEturquoise | MEblue | PF02036:SCP-2 sterol transfer family | AT5G42890.1 | SCP2 (STEROL CARRIER PROTEIN 2) |
| Pm005411 | MEturquoise | MEblue | PF00412:LIM domain | AT1G10200.1 | WLIM1 |
| Pm005431 | MEturquoise | MEblue | PF00170:bZIP transcription factor | AT1G59530.1 | BZIP4 (BASIC LEUCINE-ZIPPER 4) |
| Pm005589 | MEturquoise | MEblue | - | AT5G21940.1 | Hybrid sig-l transduction histidine kinase M-like protein |
| Pm005697 | MEturquoise | MEblue | PF03106:WRKY DNA -binding domain | AT1G80840.1 | WRKY40 (WRKY DNA-BINDING PROTEIN 40) |
| Pm005905 | MEturquoise | MEblue | PF03188:Eukaryotic cytochrome b561 | AT1G14730.1 | Cytochrome b561/ferric reductase transmembrane protein family |
| Pm006287 | MEturquoise | MEblue | PF09265:Cytokinin dehydrogenase 1, FAD and cytokinin binding\|PF01565:FAD binding domain | AT1G75450.1 | CKX5 (CYTOKININ OXIDASE 5) |
| Pm006343 | MEturquoise | MEblue | PF03171:2OG-Fe(II) oxygenase superfamily\|PF14226:non-haem dioxygenase in morphine synthesis N-terminal | AT1G06620.1 | 2-oxoglutarate (2OG) and Fe(II)-dependent oxyge-se superfamily protein |
| Pm006651 | MEturquoise | MEblue | PF00722:Glycosyl hydrolases family 16\|PF06955:Xyloglucan endo-transglycosylase (XET) C-terminus | AT1G10550.1 | XTH33 (XYLOGLUCAN:XYLOGLUCOSYL TRANSFERASE 33) |
| Pm006675 | MEturquoise | MEblue | PF03181:BURP domain | AT1G70370.1 | PG2 (POLYGALACTURONASE 2) |
| Pm006686 | MEturquoise | MEblue | PF14215:bHLH-MYC and R2R3-MYB transcription factors N-terminal | AT1G10610.1 | Basic helix-loop-helix (bHLH) DNA-binding superfamily protein |
| Pm007231 | MEturquoise | MEblue | PF13837:Myb/SANT-like DNA-binding domain | AT2G33550.1 | ASR3 (ARABIDOPSIS SH4-RELATED3) |
| Pm008039 | MEturquoise | MEblue | - | AT5G18980.1 | ARM repeat superfamily protein |
| Pm009953 | MEturquoise | MEblue | PF00450:Serine carboxypeptidase | AT1G11080.1 | SCPL31 (SERINE CARBOXYPEPTIDASE-LIKE 31) |
| Pm012358 | MEturquoise | MEblue | PF05368:NmrA-like family | AT4G39230.1 | PCBER1 (PHENYLCOUMARAN BENZYLIC ETHER REDUCTASE 1) |
| Pm013365 | MEturquoise | MEblue | PF00005:ABC transporter\|PF00664:ABC transporter transmembrane region | AT3G21250.1 | - |
| Pm015079 | MEturquoise | MEblue | PF13359:DDE superfamily endonuclease | AT5G41980.1 | - |
| Pm018226 | MEturquoise | MEblue | PF00249:Myb-like DNA-binding domain | AT1G01520.1 | RVE3 (REVEILLE 3) |
| Pm019732 | MEturquoise | MEblue | PF00520:Ion transport protein | AT5G53130.1 | CNGC1 (CYCLIC NUCLEOTIDE GATED CHANNEL 1) |
| Pm019844 | MEturquoise | MEblue | - | - | - |
| Pm022553 | MEturquoise | MEblue | PF14299:Phloem protein 2 | AT4G19840.1 | PP2-A1 (PHLOEM PROTEIN 2-A1) |
| Pm022554 | MEturquoise | MEblue | PF14299:Phloem protein 2 | AT4G19840.1 | PP2-A1 (PHLOEM PROTEIN 2-A1) |
| Pm023448 | MEturquoise | MEblue | PF02812:Glu/Leu/Phe/Val dehydrogenase, dimerisation domain\|PF00208:Glutamate/Leucine/Phenylalanine/Valine dehydrogenase | AT5G07440.1 | GDH2 (GLUTAMATE DEHYDROGENASE 2) |
| Pm023468 | MEturquoise | MEblue | PF13912:C2H2-type zinc finger | AT1G02030.1 | C2H2-like zinc finger protein |
| Pm025056 | MEturquoise | MEblue | PF00069:Protein kinase domain\|PF08263:Leucine rich repeat N-terminal domain | AT1G48480.1 | RKL1 (RECEPTOR-LIKE KINASE 1) |
| Pm025930 | MEturquoise | MEblue | PF08327:Activator of Hsp90 ATPase homolog 1-like protein | AT3G12050.1 | Aha1 domain-containing protein |
| Pm026774 | MEturquoise | MEblue | PF03094:Mlo family | AT5G53760.1 | MLO11 (MILDEW RESISTANCE LOCUS O 11) |
| Pm017800 | MEturquoise | MEblue | PF00010:Helix-loop-helix DNA-binding domain | AT4G00050.1 | basic helix-loop-helix (bHLH) DNA-binding superfamily protein |
| Pm000880 | MEyellow | MEbrown | PF02466:Tim17/Tim22/Tim23/Pmp24 family | AT2G28900.1 | OEP16 (OUTER ENVELOPE PROTEIN 16) |
| Pm004544 | MEyellow | MEbrown | - | AT1G78170.1 | E3 ubiquitin-protein ligase |
| Pm006324 | MEyellow | MEbrown | PF06200:tify domain\|PF09425:Divergent CCT motif | AT3G17860.1 | - |
| Pm006589 | MEyellow | MEbrown | PF00201:UDP-glucoronosyl and UDP-glucosyl transferase | AT1G22360.1 | UDP-glucosyl transferase 85A2 (cytokinin-O-glucosyltransferase 2) |
| Pm010133 | MEyellow | MEbrown | PF00657:GDSL-like Lipase/Acylhydrolase | AT5G45910.1 | GDSL-motif esterase/acyltransferase/lipase |
| Pm024587 | MEyellow | MEbrown | PF00010:Helix-loop-helix DNA-binding domain | AT3G26744.1 | ICE1 (INDUCER OF CBF EXPRESSION 1) |
| Pm026120 | MEyellow | MEbrown | PF00561:alpha/beta hydrolase fold | AT3G03990.1 | D14 (DWARF 14) |
| Pm004488 | MEblue | MEturquoise | PF13639:Ring finger domain | AT1G72220.1 | RING/U-box superfamily protein |
| Pm005973 | MEblue | MEturquoise | PF05153:Myo-inositol oxygenase | AT1G14520.1 | MIOX1 (MYO-INOSITOL OXYGENASE 1) |
| Pm010322 | MEblue | MEturquoise | PF00664:ABC transporter transmembrane region\|PF00005:ABC transporter | AT4G18050.1 | PGP9 (P-GLYCOPROTEIN 9) |
| Pm011542 | MEblue | MEturquoise | PF02485:Core-2/I-Branching enzyme | AT3G15350.1 | Core-2/I-branching beta-1,6-N-acetylglucosaminyltransferase family protein |
| Pm014367 | MEblue | MEturquoise | - | - | - |
| Pm016358 | MEblue | MEturquoise | PF10250:GDP-fucose protein O-fucosyltransferase | AT2G44500.1 | O-fucosyltransferase family protein |
| Pm024844 | MEblue | MEturquoise | - | - | - |
| Pm000375 | MEturquoise | MEturquoise | PF00225:Kinesin motor domain\|PF00307:Calponin homology (CH) domain | AT3G44730.1 | Kinesin-like protein 1 |
| Pm000513 | MEturquoise | MEturquoise | PF04859:Plant protein of unknown function (DUF641) | AT5G58960.1 | - |
| Pm000556 | MEturquoise | MEturquoise | PF00909:Ammonium Transporter Family | AT1G64780.1 | AMT1;2 (AMMONIUM TRANSPORTER 1;2) |
| Pm000832 | MEturquoise | MEturquoise | PF16211:C-terminus of histone H2A\|PF00125:Core histone H2A/H2B/H3/H4 | AT5G02560.1 | - |
| Pm000908 | MEturquoise | MEturquoise | PF01357:Pollen allergen\|PF03330:Rare lipoprotein A (RlpA)-like double-psi beta-barrel | AT2G39700.1 | EXP4 (EXPANSIN A4) |
| Pm000955 | MEturquoise | MEturquoise | - | AT2G37080.1 | ICR2 (INTERACTOR OF CONSTITUTIVELY ACTIVE ROP2) |
| Pm000956 | MEturquoise | MEturquoise | - | AT5G60210.1 | RIP5 (ROP INTERACTIVE PARTNER 5) |
| Pm000958 | MEturquoise | MEturquoise | PF02701:Dof domain, zinc finger | AT2G28510.1 | DOF2.1 (DOF PROTEIN 2.1) |
| Pm001067 | MEturquoise | MEturquoise | - | - | - |
| Pm001085 | MEturquoise | MEturquoise | PF00005:ABC transporter\|PF01061:ABC-2 type transporter | AT2G28070.1 | ABC-2 type transporter family protein |
| Pm004242 | MEturquoise | MEturquoise | PF00201:UDP-glucoronosyl and UDP-glucosyl transferase | AT3G16520.1 | UGT88A1 (UDP-GLUCOSYL TRANSFERASE 88A1) |
| Pm004330 | MEturquoise | MEturquoise | PF00010:Helix-loop-helix DNA-binding domain | AT5G65640.1 | BHLH093 (BETA HLH PROTEIN 93) |
| Pm004353 | MEturquoise | MEturquoise | PF00067:Cytochrome P450 | AT4G31950.1 | CYP82C3 (CYTOCHROME P450, FAMILY 82, SUBFAMILY C, POLYPEPTIDE 3) |
| Pm004415 | MEturquoise | MEturquoise | PF01486:K-box region\|PF00319:SRF-type transcription factor (DNA-binding and dimerisation domain) |  | DAM6 (DORMANCY ASSOCIATED MADS-BOX 6) |
| Pm004416 | MEturquoise | MEturquoise | PF01486:K-box region\|PF00319:SRF-type transcription factor (DNA-binding and dimerisation domain) |  | DAM5 (DORMANCY ASSOCIATED MADS-BOX 5) |
| Pm004417 | MEturquoise | MEturquoise | PF01486:K-box region\|PF00319:SRF-type transcription factor (DNA-binding and dimerisation domain) |  | DAM4 (DORMANCY ASSOCIATED MADS-BOX 4) |
| Pm004481 | MEturquoise | MEturquoise | PF02298:Plastocyanin-like domain | AT1G72230.1 | Cupredoxin superfamily protein |
| Pm004523 | MEturquoise | MEturquoise | PF04770:ZF-HD protein dimerisation region | AT4G24660.1 | HB22 (HOMEOBOX PROTEIN 22) |
| Pm004555 | MEturquoise | MEturquoise | PF02362:B3 DNA binding domain | - | - |
| Pm004556 | MEturquoise | MEturquoise | PF06645:Microsomal signal peptidase 12 kDa subunit (SPC12) | AT1G34640.1 | Peptidase |
| Pm004575 | MEturquoise | MEturquoise | PF00201:UDP-glucoronosyl and UDP-glucosyl transferase | AT1G22360.1 | UDP-glucosyl transferase 85A2 (cytokinin-O-glucosyltransferase 2) |
| Pm004740 | MEturquoise | MEturquoise | PF12819:Carbohydrate-binding protein of the ER\|PF07714:Protein tyrosine kinase\|PF00134:Cyclin, N-terminal domain\|PF02984:Cyclin, C-terminal domain | AT4G29990.1 | Leucine-rich repeat transmembrane protein ki-se protein |
| Pm004898 | MEturquoise | MEturquoise | PF00641:Zn-finger in Ran binding protein and others | AT3G15680.1 | Ran BP2/NZF zinc finger-like superfamily protein |
| Pm004987 | MEturquoise | MEturquoise | - | - | - |
| Pm005079 | MEturquoise | MEturquoise | PF00288:GHMP kinases N terminal domain\|PF13528:Glycosyl transferase family 1\|PF10509:Galactokinase galactose-binding signature | AT4G16130.1 | ARA1 (ARABINOSE KINASE) |
| Pm005099 | MEturquoise | MEturquoise | PF04782:Protein of unknown function (DUF632)\|PF04783:Protein of unknown function (DUF630) | AT5G25590.1 | Protein of unknown function (DUF630) |
| Pm005218 | MEturquoise | MEturquoise | - | - | - |
| Pm005274 | MEturquoise | MEturquoise | PF08553:VID27 cytoplasmic protein | AT4G33400.1 | Vid27-related protein |
| Pm005326 | MEturquoise | MEturquoise | PF02984:Cyclin, C-terminal domain\|PF00134:Cyclin, N-terminal domain | AT4G34160.1 | CYCD3 (CYCLIN D3) |
| Pm005440 | MEturquoise | MEturquoise | PF00847:AP2 domain | AT4G37750.1 | ANT (AINTEGUMENTA) |
| Pm006186 | MEturquoise | MEturquoise | PF00069:Protein kinase domain | AT1G75820.1 | CLV1 (CLAVATA 1) |
| Pm006237 | MEturquoise | MEturquoise | PF06507:Auxin response factor\|PF02309:AUX/IAA family\|PF02362:B3 DNA binding domain | AT1G19850.1 | ARF5 (AUXIN RESPONSE FACTOR 5) |
| Pm006466 | MEturquoise | MEturquoise | PF13414:TPR repeat\|PF00856:SET domain | AT1G26760.1 | SDG35 (SET DOMAIN PROTEIN 35) |
| Pm006483 | MEturquoise | MEturquoise | PF00249:Myb-like DNA-binding domain | AT1G69560.1 | MYB105 (MYB DOMAIN PROTEIN 105) |
| Pm006520 | MEturquoise | MEturquoise | PF00415:Regulator of chromosome condensation (RCC1) repeat\|PF13713:Transcription factor BRX N-terminal domain\|PF01363:FYVE zinc finger\|PF08381:Transcription factor regulating root and shoot growth via Pin3 | AT5G19420.1 | Regulator of chromosome condensation (RCC1) family with FYVE zinc finger domain |
| Pm006597 | MEturquoise | MEturquoise | PF00069:Protein kinase domain | AT1G69910.1 | Protein kinase superfamily protein |
| Pm006603 | MEturquoise | MEturquoise | PF01762:Galactosyltransferase\|PF00337:Galactoside-binding lectin | AT5G62620.1 | GALT6 |
| Pm006672 | MEturquoise | MEturquoise | PF06075:Plant protein of unknown function (DUF936) | AT1G23790.1 | Dicer-like protein |
| Pm006826 | MEturquoise | MEturquoise | PF00106:short chain dehydrogenase | AT1G24470.1 | KCR2 (BETA-KETOACYL REDUCTASE 2) |
| Pm006907 | MEturquoise | MEturquoise | PF03763:Remorin, C-terminal region | AT1G67590.1 | Remorin family protein |
| Pm007095 | MEturquoise | MEturquoise | PF02182:SAD/SRA domain\|PF00097:Zinc finger, C3HC4 type (RING finger) | AT1G57820.1 | VIM1 (VARIANT IN METHYLATION 1) |
| Pm007217 | MEturquoise | MEturquoise | PF00069:Protein kinase domain | AT5G13290.1 | SOL2 (SUPPRESSOR OF LLP1 2) |
| Pm008977 | MEturquoise | MEturquoise | PF14868:Domain of unknown function (DUF4487)\|PF05938:Plant self-incompatibility protein S1 | AT1G04650.1 | FLIP (FIDGETIN-LIKE-1 INTERACTING PROTEIN) |
| Pm008979 | MEturquoise | MEturquoise | - | - |  |
| Pm009669 | MEturquoise | MEturquoise | PF07732:Multicopper oxidase\|PF07731:Multicopper oxidase\|PF00394:Multicopper oxidase | AT4G22010.1 | SKS4 (SKU5 SIMILAR 4) |
| Pm010135 | MEturquoise | MEturquoise | PF09585:Conserved hypothetical protein (Lin0512_fam) | AT4G18570.1 | IPGA1 (INCREASED PETAL GROWTH ANISOTROPY 1) |
| Pm010363 | MEturquoise | MEturquoise | PF02362:B3 DNA binding domain | AT2G33860.1 | ARF3 (AUXIN RESPONSE TRANSCRIPTION FACTOR 3) |
| Pm011556 | MEturquoise | MEturquoise | - | - | - |
| Pm013628 | MEturquoise | MEturquoise | - | - | - |
| Pm014159 | MEturquoise | MEturquoise | PF06886:Targeting protein for Xklp2 (TPX2)\|PF12214:Cell cycle regulated microtubule associated protein | AT1G03780.1 | TPX2 (TARGETING PROTEIN FOR XKLP2) |
| Pm014341 | MEturquoise | MEturquoise | PF00538:linker histone H1 and H5 family | AT1G14900.1 | HMGA (HIGH MOBILITY GROUP A) |
| Pm014750 | MEturquoise | MEturquoise | PF03188:Eukaryotic cytochrome b561 | AT2G30890.1 | Cytochrome b561/ferric reductase transmembrane protein family |
| Pm016424 | MEturquoise | MEturquoise | PF05678:VQ motif | AT2G44340.1 | VQ18 (VQ PROTEIN 18) |
| Pm016754 | MEturquoise | MEturquoise | PF03999:Microtubule associated protein (MAP65/ASE1 family) | AT2G38720.1 | MAP65-5 (MICROTUBULE-ASSOCIATED PROTEIN 65-5) |
| Pm016914 | MEturquoise | MEturquoise | - | AT3G52490.1 | SMXL3 (SMAX1-LIKE 3) |
| Pm017199 | MEturquoise | MEturquoise | PF13641:Glycosyltransferase like family 2 | AT5G03760.1 | CSLA9 (CELLULOSE SYNTHASE LIKE A9) |
| Pm018092 | MEturquoise | MEturquoise | PF02826:D-isomer specific 2-hydroxyacid dehydrogenase, NAD binding domain\|PF00389:D-isomer specific 2-hydroxyacid dehydrogenase, catalytic domain | AT2G45630.1 | HPPR4 (HYDROXYPHENYLPYRUVATE REDUCTASE 4) |
| Pm018625 | MEturquoise | MEturquoise | PF06232:Embryo-specific protein 3, (ATS3) | AT2G41475.1 | ATS3A (EMBRYO-SPECIFIC PROTEIN 3A) |
| Pm018822 | MEturquoise | MEturquoise | PF03514:GRAS domain family | AT2G37650.1 | GRAS family transcription factor |
| Pm018857 | MEturquoise | MEturquoise | PF00201:UDP-glucoronosyl and UDP-glucosyl transferase | AT3G55700.1 | UDP-GLYCOSYLTRANSFERASE 76F1 (cytokinin-N-glucosyltransferase 1) |
| Pm019129 | MEturquoise | MEturquoise | PF12854:PPR repeat\|PF13041:PPR repeat family | AT1G19720.1 | Pentatricopeptide repeat (PPR-like) superfamily protein |
| Pm019146 | MEturquoise | MEturquoise | PF01553:Acyltransferase | AT4G01950.1 | Glycerol-3-phosphate acyltransferase 3 |
| Pm019358 | MEturquoise | MEturquoise | PF13855:Leucine rich repeat\|PF08263:Leucine rich repeat N-terminal domain\|PF07714:Protein tyrosine kinase | AT5G07280.1 | EMS1 (EXCESS MICROSPOROCYTES1) |
| Pm019758 | MEturquoise | MEturquoise | - | AT4G00990.1 | JMJ27 (JMJC DOMAIN-CONTAINING PROTEIN 27) |
| Pm019806 | MEturquoise | MEturquoise | PF04821:Timeless protein\|PF13855:Leucine rich repeat\|PF05029:Timeless protein C terminal region | AT5G52910.1 | Timeless family protein |
| Pm019824 | MEturquoise | MEturquoise | PF03168:Late embryogenesis abundant protein | - | - |
| Pm019825 | MEturquoise | MEturquoise | PF00005:ABC transporter\|PF01061:ABC-2 type transporter | AT5G52860.1 | ATP-BINDING CASSETTE G8 (ABCG8) |
| Pm019857 | MEturquoise | MEturquoise | PF05684:Protein of unknown function (DUF819) | AT5G52540.1 | - |
| Pm021351 | MEturquoise | MEturquoise | PF02362:B3 DNA binding domain | AT3G19184.1 | AP2/B3-like transcriptio-l factor family protein |
| Pm021773 | MEturquoise | MEturquoise | PF05739:SNARE domain\|PF00804:Syntaxin | AT2G18260.1 | SYP112 (SYNTAXIN OF PLANTS 112) |
| Pm022681 | MEturquoise | MEturquoise | PF03405:Fatty acid desaturase | AT1G43800.1 | FTM1 (FLORAL TRANSITION AT THE MERISTEM1) |
| Pm022714 | MEturquoise | MEturquoise | - | - | - |
| Pm022819 | MEturquoise | MEturquoise | PF10539:Development and cell death domain | AT3G27090.1 | DCD (Development and Cell Death) domain protein |
| Pm023460 | MEturquoise | MEturquoise | PF14389:Leucine-zipper of ternary complex factor MIP1\|PF04784:Protein of unknown function, DUF547 | AT5G47380.1 | Electron transporter |
| Pm024077 | MEturquoise | MEturquoise | PF00505:HMG (high mobility group) box | AT4G23800.1 | 3XHMG-BOX2 (3XHIGH MOBILITY GROUP-BOX2) |
| Pm024257 | MEturquoise | MEturquoise | PF08880:QLQ\|PF08879:WRC | AT4G24150.1 | GRF8 (GROWTH-REGULATING FACTOR 8) |
| Pm024725 | MEturquoise | MEturquoise | PF00249:Myb-like DNA-binding domain | AT5G14750.1 | MYB66 (MYB DOMAIN PROTEIN 66) |
| Pm025107 | MEturquoise | MEturquoise | PF00867:XPG I-region\|PF00752:XPG N-terminal domain | AT1G18090.1 | 5-3 exonuclease family protein |
| Pm025275 | MEturquoise | MEturquoise | PF04770:ZF-HD protein dimerisation region | AT3G50890.1 | HB28 (HOMEOBOX PROTEIN 28) |
| Pm026186 | MEturquoise | MEturquoise | PF00190:Cupin | AT3G10080.1 | RmlC-like cupins superfamily protein |
| Pm022634 | MEturquoise | MEturquoise | PF01582:TIR domain\|PF00931:NB-ARC domain | AT5G17680.1 | disease resistance protein (TIR-NBS-LRR class), putative |
| Pm004972 | MEturquoise | MEturquoise | PF00743:Flavin-binding monooxygenase-like | AT5G11320.1 | Flavin-binding monooxygenase family protein |
| Pm026237 | MEturquoise | MEturquoise | PF06974:Protein of unknown function (DUF1298)\|PF03007:Wax ester synthase-like Acyl-CoA acyltransferase domain | AT5G53390.1 | O-acyltransferase (WSD1-like) family protein |
| Pm000518 | MEyellow | MEyellow | PF00343:Carbohydrate phosphorylase | AT3G46970.1 | ATPHS2 (ALPHA-GLUCAN PHOSPHORYLASE 2) |
| Pm004655 | MEyellow | MEyellow | - | AT1G80490.1 | TPR1 (TOPLESS-RELATED 1) |
| Pm018083 | MEyellow | MEyellow | PF03171:2OG-Fe(II) oxygenase superfamily\|PF14226:non-haem dioxygenase in morphine synthesis N-terminal | AT4G25420.1 | GA20OX1 (GIBBERELLIN 20 OXIDASE 1) |
